# MICa/b‐dependent activation of natural killer cells by CD64 ^+^ inflammatory type 2 dendritic cells contributes to autoimmunity

**DOI:** 10.15252/embj.2023113714

**Published:** 2023-11-02

**Authors:** Ildefonso Sánchez‐Cerrillo, Diego Calzada‐Fraile, Ana Triguero‐Martínez, Marta Calvet‐Mirabent, Olga Popova, Cristina Delgado‐Arévalo, Mariel Valdivia‐Mazeyra, Marta Ramírez‐Huesca, Enrique Vázquez de Luis, Alberto Benguría, Teresa Aceña‐Gonzalo, Roberto Moreno‐Vellisca, Magdalena Adrados de Llano, Hortensia de la Fuente, Ilya Tsukalov, Pablo Delgado‐Wicke, Elena Fernández‐Ruiz, Emilia Roy‐Vallejo, Reyes Tejedor‐Lázaro, Almudena Ramiro, Salvador Iborra, Francisco Sánchez‐Madrid, Ana Dopazo, Isidoro González Álvaro, Santos Castañeda, Enrique Martin‐Gayo

**Affiliations:** ^1^ Immunology Unit Hospital Universitario La Princesa, Medicine Department, Universidad Autónoma de Madrid, Instituto Investigación Sanitaria‐Princesa IIS‐IP Madrid Spain; ^2^ Vascular Pathophysiology Department Centro Nacional de Investigaciones Cardiovasculares Madrid Spain; ^3^ Rheumatology Unit Hospital Universitario La Princesa, Instituto de Investigación Sanitaria‐Princesa IIS‐IP Madrid Spain; ^4^ Pathology Unit Hospital Universitario La Princesa Madrid Spain; ^5^ Genomic Unit Centro Nacional de Investigaciones Cardiovasculares Madrid Spain; ^6^ CIBER Cardiovascular, Instituto de Salud Carlos III Madrid Spain; ^7^ Cátedra UAM‐Roche, EPID‐Future, Department of Medicine Universidad Autónoma de Madrid (UAM) Madrid Spain; ^8^ CIBER Enfermedades Infecciosas (CIBERINFECC), Instituto de Salud Carlos III Madrid Spain

**Keywords:** dendritic cells, interferon, natural killer cells, RIG‐I, Sjögren's syndrome, Immunology

## Abstract

Primary Sjögren's syndrome (pSS) is an inflammatory autoimmune disorder largely mediated by type I and II interferon (IFN). The potential contribution of innate immune cells, such as natural killer (NK) cells and dendritic cells (DC), to the pSS pathology remains understudied. Here, we identified an enriched CD16^+^ CD56hi NK cell subset associated with higher cytotoxic function, as well as elevated proportions of inflammatory CD64^+^ conventional dendritic cell (cDC2) subtype that expresses increased levels of MICa/b, the ligand for the activating receptor NKG2D, in pSS individuals. Circulating cDC2 from pSS patients efficiently induced activation of cytotoxic NK cells *ex vivo* and were found in proximity to CD56^+^ NK cells in salivary glands (SG) from pSS patients. Interestingly, transcriptional activation of IFN signatures associated with the RIG‐I/DDX60 pathway, IFN I receptor, and its target genes regulate the expression of NKG2D ligands on cDC2 from pSS patients. Finally, increased proportions of CD64hi RAE‐1^+^ cDC2 and NKG2D^+^CD11b^+^CD27^+^ NK cells were present *in vivo* in the SG after poly I:C injection. Our study provides novel insight into the contribution and interplay of NK and cDC2 in pSS pathology and identifies new potential therapy targets.

## Graphical abstract

While Interferons are known to be involved in primary Sjögren's syndrome (pSS), work shown here also supports a role for innate immune cells like natural killer and dendritic cells in disease pathology.

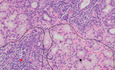

## Introduction

Primary Sjögren's syndrome (pSS) is a complex autoimmune disorder characterized by the activation of type I and II interferon (IFN) responses in the salivary gland (SG) and peripheral blood (PB) (Emamian *et al*, 2009; Hall *et al*, 2012; Bodewes *et al*, 2018, 2019. These responses usually lead to inflammation of salivary and/or lacrimal glands, and in some cases extra‐glandular manifestations involving damage of lung, kidney, systemic vasculitis, or lymphoma development (Skopouli *et al*, 2000). Different innate and adaptive immune cell subsets infiltrate into the salivary glands (SG) during the course of the disease, which appear to be differentially represented depending on the severity level of lesions (Christodoulou *et al*, 2010). Altered adaptive immune responses mediated by Th17 cells (Katsifis *et al*, 2009; Li *et al*, 2015), CD8^+^ T cells (Gao *et al*, 2019; Zhou *et al*, 2020), and autoreactive B cells generating ectopic germinal centers (GC) have been well characterized in the SG from pSS patients and associate with tissue destruction (Hansen *et al*, 2002, 2010). However, the connection between such pathogenic adaptive immune profiles and specific innate immune cell subsets affected by IFN has not been sufficiently explored. Infiltration of innate immune cell types such as natural killer (NK) cells and myeloid cells including monocytes (Mo) and dendritic cells (DC) have been observed in SG from pSS patients even prior to the development of severe lesions containing GC (Christodoulou *et al*, 2010), suggesting an active role of innate immunity during the development and/or progression of the pathology. Although an altered activation state of NK cells from the peripheral blood (PB) and SGs from pSS patients has been suggested (Rusakiewicz *et al*, 2013; Ming *et al*, 2020), little is known about whether these parameters may associate to changes in specific NK cell subpopulations displaying specific functional characteristics. In addition, the mechanisms potentially driving the pathogenic activation of NK cells during the development of pSS, the crosstalk with other immune cell populations, and their contribution to the progression of the disease are not fully understood. Transcriptional and genome‐wide association (GWAS) studies point to alterations in innate signaling pathways, including IFN regulatory factors (Lessard *et al*, 2013). However, the potential cellular and molecular mechanisms responsible for such deregulated IFN response in pSS and their implication in NK cell activation and pathology are currently a matter of active debate. Myeloid cells express innate sensors capable of triggering IFN in response to Pathogen and Damage Associated Molecular Patterns (PAMPs and DAMPs, respectively) and could contribute to this process. Also, recognition of PAMPs and DAMPs such as endogenous RNA and DNA can activate myeloid cells and lead to pathogenic inflammatory states that contribute to autoimmune disorders. Inflammatory CD16^+^ monocytes (Mo), plasmacytoid DCs (pDC), and CD11c^+^ conventional DCs (cDC) are recruited to the SGs in pSS patients, especially at initial stages of the disease (Wildenberg *et al*, 2009). pDCs have been traditionally recognized as type I IFN producers during viral infections (Gilliet *et al*, 2008; Solano‐Galvez *et al*, 2018; Rhodes *et al*, 2019). In the context of pSS, pDCs have been proposed to produce IFNα in SG after stimulation of endosomal Toll like Receptor (TLR) 7 presumably by RNA complexes (Båve *et al*, 2005; Lövgren *et al*, 2006). On the other hand, CD11c^+^ cDC is a heterogeneous group comprised by cDC1 (CD141^+^) and cDC2 (CD1c^+^) subsets, differing at their functional abilities to support T cells and to respond to different types of PAMPs and DAMPs, including viral nucleic acids (Jongbloed *et al*, 2010; O'Keeffe *et al*, 2015; Castenmiller *et al*, 2021). The complexity of cDC subsets has increased recently, after the identification of a new subpopulation of inflammatory CD64^+^ cDC2 with antiviral properties in respiratory and HIV infections (Martin‐Gayo *et al*, 2018; Bosteels *et al*, 2020). However, little is known about the role of inflammatory CD64^+^ cDC2 in autoimmune disorders mediated by IFNs such as pSS. Previous studies reported the depletion of CD11c^+^ cDC from the blood of pSS patients, but it is unknown whether specific cDC subsets are affected. In addition, although altered expression of intracellular RNA sensors has been reported in SG and peripheral blood mononuclear cells (PBMC) from pSS (Hall *et al*, 2012; Maria *et al*, 2017), there is no information about whether activation of these pathways occur on specific cDC subsets during the course of the disease. In this regard, IFN transcriptional signatures have been reported in cDC2 from pSS individuals (Lopes *et al*, 2022), but such activation patterns and functional properties have not been compared with other myeloid subsets such as cDC1 and Mo. Finally, the majority of studies have mainly focused on addressing the pathogenic role of cDC2 directly activating CD4^+^ or CD8^+^ T cell responses in the context of autoimmunity, without considering the potential influence of other innate immune cells such as NK cells. In this regard, cDC1 and cDC2 can significantly impact the activation and maturation of NK cells (Ferlazzo & Morandi, 2014; Pallazola *et al*, 2021) and might contribute to the activation of these cells in pSS (Rusakiewicz *et al*, 2013; Gianchecchi *et al*, 2021). However, which molecules expressed by different DC subsets may determine their ability to activate NK cells in SG, the underlying mechanisms regulating their expression, and the potential impact on subsequent pathogenic adaptive immune responses in pSS remain to be determined. In this regard, murine models inducing IFN responses and recapitulating some aspects of the pSS pathology such as immune cell infiltration and damage in the SG (Nandula *et al*, 2011, 2013) could be a useful tool to address the involvement of specific cDC and NK cell subsets and their potential association with the induction of pathogenic adaptive immune cells such as Th17 (Ciccia *et al*, 2012; Lin *et al*, 2015) and autoreactive B cells induced during the disease.

In the present study, we identified increased frequency of a CD56^hi^ CD16^+^ NKG2D^hi^ cell subset that associates with enhanced cytotoxic function of NK cells from pSS patients in PB and in the SG. We also provide new data showing that increased expression of ligands for NKG2D by inflammatory CD64^+^ cDC2 from pSS patients associates with greater ability to induce maturation of cytotoxic NK cells. In addition, higher expression of specific antiviral transcriptional signatures characterized by intracellular RNA sensing pathways was present in cDC2 compared to cDC1 and Mo from pSS. Specifically, DDX60 and RIG‐I helicases, the signaling through IFN I receptor and the IFN target gene IFIT1 are required for the expression of MICa/b on cDC2. Finally, early infiltration and activation of NK and inflammatory cDC2 can be found *in vivo* in the salivary gland of mice injected with the RIG‐I/TLR3 agonist poly I:C. Therefore, our study provides novel insights on cellular and molecular mechanisms driving pathology in pSS and identifies new potential targets for future therapies.

## Results

### Increased cytotoxic CD56^hi^ CD16 ^+^ NK cell subset is enriched in pSS patients

**Figure 1:**
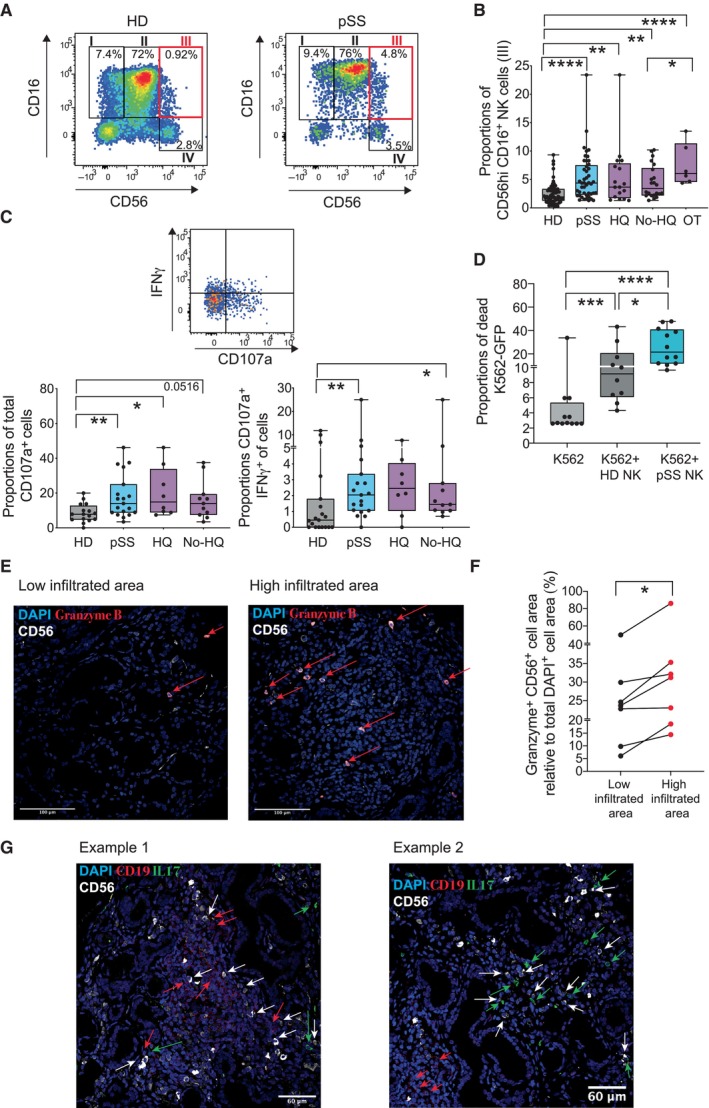
Proportions and phenotypical characteristics of natural killer cell subsets from pSS patients AFlow cytometry dot plots showing analysis of NK cell subsets present in Lineage (CD3, CD19, CD14) negative, HLADR negative lymphocytes, and defined by CD16 vs. CD56 expression in the PB of a representative HD (left) and pSS (right) individuals. Gating defining CD56^hi^ CD16^+^ NK cells (III) is highlighted in red.BSummarized proportions of CD56^hi^ CD16^+^ circulating NK cells in 56 healthy donors (HD; gray) and total 48 primary Sjögren's syndrome (pSS; blue) patients or stratified according to the absence (No HQ, *n* = 25 biological replicates) or the presence of hydroxychloroquine (HQ; *n* = 17 biological replicates) or other (OT; *n* = 6 biological replicates) treatments (purple).CAnalysis of expression of IFNγ and CD107a on NK cells. Representative flow cytometry dot plot is included in the top. Proportions of total CD107a^+^ (bottom, left) and IFNγ^+^ CD107a^+^ (bottom, right) cells within the CD56^hi^ CD16^+^ NK cell subset from *n* = 16 HD (gray) and *n* = 19 total or *n* = 8 HQ and *n* = 11 No‐HQ pSS patients (blue).DFunctional characteristics of NK cells from pSS patients. Summary of proportions of dead target K562‐GFP cells cultured for 16 h in the absence (light gray) or the presence of isolated circulating NK cells from HD (*n* = 10; biological replicates) or pSS (*n* = 12 biological replicates) patients.E–GHistological immunofluorescence analysis of merged expression of CD56 (white) and Granzyme B (red) on low (left) and highly (right) infiltrated glandular areas (E) or with CD19 (red) and IL‐17 (green) (G) from the section of SG tissue from a representative pSS patient. Cell nuclei were stained with DAPI (blue). Cells co‐expressing CD56 and Granzyme B (E) or expressing CD19 (G) are highlighted with red arrows. In panel (G), IL17^+^ and CD56^+^ are also highlighted in green and white arrows, respectively. Original magnification 40×. (F): Image J quantification of proportions of area of CD56^+^ NK cells co‐expressing Granzyme B within the total the mentioned total high and low infiltrated areas from *n* = 7 tested pSS patients. Data are normalized to the number of total DAPI^+^ cells detected on each area considered as 100%. Data information: (B–D) Data are represented as box and whiskers plots with maximum and minimum range and a median value central band. Statistical significance was calculated with a two‐tailed Mann Whitney test. *P < 0.05; **P < 0.01; ***P < 0.001; ****P < 0.0001. Source data are available online for this figure.

We analyzed proportions and phenotype of different NK cell subsets in PB samples from a cohort of 48 pSS individuals compared to PB from 56 healthy donors (HD). Clinical characteristics of participants are shown in Appendix Table S1. Briefly, a high proportion (64%) of pSS individuals displayed mild disease and 21% were considered severe according to clinical ESSDAI guidelines (Seror *et al*, 2015, 2016), which include the presence of extra‐glandular manifestations (Appendix Table S1). Thirty‐five percent of pSS patients received hydroxychloroquine (HQ) treatment, and this group was significantly enriched in severe individuals (35 vs. 16%; *P* = 0.002), the presence of extra‐glandular manifestations (76 vs. 32%; *P* < 0.0001), and a trend to more marked alterations in levels of parameters associated with pathology such as lymphopenia, higher levels of IgG, and significantly lower levels of complement C3 (Appendix Table S1). In contrast, patients that did not receive HQ were more enriched in mild patients (84 vs. 64%; *P* = 0.0013, Appendix Table S1). Finally, some individuals (*n* = 6) from our pSS cohort received other treatments (OT) including biologicals, methotrexate and glucocorticoids, and were all characterized by severe pathology and the presence of extraglandular manifestations (Appendix Table S1). Treatment in the OT pSS group was associated with an improvement of some clinical parameters such as erythrocyte sedimentation rate (ESR) and lymphopenia compared to the HQ group (Appendix Table S1). Although a small percentage of pSS individuals developed comorbidities such as arthritis (33%), no significant alterations in other clinical parameters were found (Appendix Table S1). Proportions of a CD56^hi^ CD16^+^ NK cell subset were significantly increased in the blood of all groups of pSS patients, including those in HQ and OT treatments, suggesting that the increase in this population is a biomarker of the disease rather than disease activity (Fig 1A and B). In contrast, CD56^hi^ CD16^−^ cells, were less significantly affected (Fig 1A and Appendix Fig S1A). Interestingly, frequencies of CD56^hi^ CD16^+^ NK cells were even more enriched in pSS receiving OT (Fig 1B). In contrast, frequencies of CD56^dim^ CD16^+^ and CD56^−^ CD16^+^ NK subsets were not significantly affected in the PB of pSS patients (Appendix Fig S1A and Table S2). Since maturation of NK cells into CD16^+^ cells involves the acquisition of cytotoxic function and polyfunctionality, we addressed the intracellular expression of the degranulation marker CD107a and its co‐expression with effector cytokines such as IFNγ and TNFα on NK cell subsets from pSS patients. For this analysis, we focused on HQ and non‐HQ pSS patients since alterations in CD56^hi^ CD16^+^ NK cells were more representative of the total pSS population than the OT pSS group (Fig 1B). Interestingly, CD56^hi^ CD16^+^, CD56^dim^ CD16^+^, and CD56^−^ CD16^+^ NK cell subsets from pSS expressed significantly higher total levels of CD107a, more significantly on the HQ group, but followed a similar trend in the untreated patients (Fig 1C, and Appendix Fig S1B and C, and Table S2). Proportions of total IFNγ^+^ cells were also significantly higher in CD56^hi^ CD16^+^ and CD56^−^ CD16^+^ NK cells from pSS patients, while IFNγ^+^ cells within CD56^dim^ CD16^+^ and CD56^hi^ CD16^−^ NK cell populations were only significantly increased in untreated pSS patients (Appendix Fig S1B and C, and Table S2). Moreover, all analyzed NK cell subsets from pSS specifically were enriched in significantly higher proportions of polyfunctional IFNγ^+^ CD107a^+^ cells compared to HD mostly in untreated pSS individuals (Fig 1C, and Appendix Fig S1C and Table S2). In addition, CD56^hi^ CD16^+^ and CD56^hi^ CD16^−^ NK cells also tended to express higher levels of TNFα in HQ pSS patients (Appendix Fig S1B and C). Thus, different NK cell subsets from HQ and Non‐HQ pSS patients are characterized by an activated phenotype skewed toward a cytotoxic and/or polyfunctional phenotype. We then evaluated the expression of activating and inhibitory receptors on NK cell subsets from pSS compared to HD (Appendix Fig S2A). Interestingly, increased proportions of cells with high levels of expression of the activating receptor NKG2D were observed in all NK cell subsets from the PB of pSS, suggesting potentially higher susceptibility to activation in these cells (Appendix Fig S2A). Notably, this effect was present in all pSS patients, but it was more marked and differences were more significant in NK cells from individuals receiving HQ (Appendix Fig S2A and Table S2). A similar trend to higher expression of NKp30 was also observed in NK from pSS, although differences in this case were most evident in pSS patients that did not receive HQ, and less significant compared to HD. In the case of the inhibitory receptor NKG2A, significantly higher proportions of NKG2A^hi^ cells were found only in CD56^dim^ CD16^+^ NK cells (Appendix Fig S2A). Therefore, our data indicate that NK cells from pSS are enriched in cell subsets displaying higher cytotoxic potential. Supporting this possibility, total CD56^+^ NK cells isolated *ex vivo* from pSS patients exhibited significantly higher cytotoxic function toward K562‐GFP target cells compared to cells from HD (Fig 1D and Appendix Fig S2B). To explore whether an increased activation and cytotoxic state might also be present in NK cells at pathological sites from pSS patients, we evaluated the expression of granzyme B in NK cells from SGs tissue sections of these patients, as a readout of cytotoxic cells. CD56^+^ NK cells were detected at areas of glandular tissue with both low and high level of infiltration characterized by different degrees of altered tissue structure (Fig 1E and Appendix Fig S2C). However, detection of CD56^+^ NK cells co‐expressing Granzyme B was higher in high infiltrated areas (Fig 1E and F). Notably, NK cells were detected in highly infiltrated areas from SG containing IL‐17^+^ and B cells, which are known to participate in the pathology and damage to glandular tissue (Fig 1G). Together, our data indicate that NK cells from pSS individuals are enriched on a CD56^hi^ CD16^+^ cell subset associated with a more activated and elevated cytotoxic state in PB and SG and some of the identified biomarkers may be preferentially represented in HQ‐treated individuals characterized by higher severity.

### Increased expression of NKG2D ligands in cDC2 from pSS patients associates with enhanced ability to activate cytotoxic NK cells

**Figure 2:**
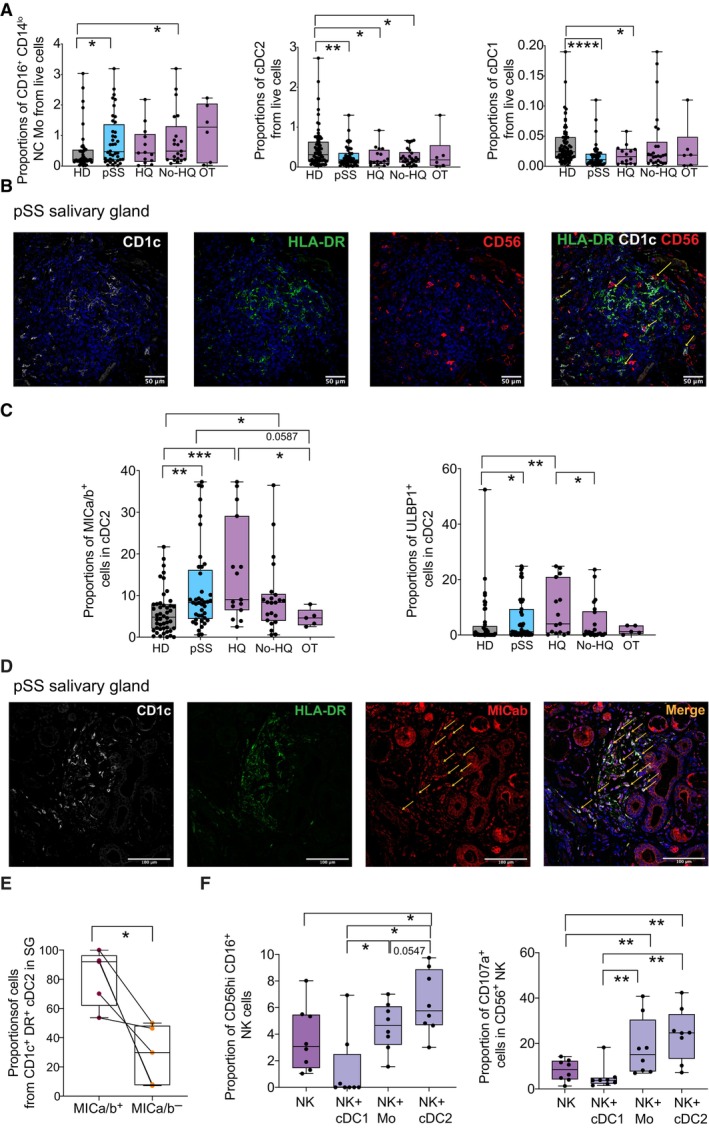
Frequencies, phenotypic and functional characteristics of myeloid cell subsets from pSS patients ABox and whiskers plots showing proportions from live lymphocytes of non‐classical (NC; right) CD16^+^ CD14^lo^ Mo (left), cDC2 (middle), and cDC1 (right) in the PB from HD (gray; *n* = 56 biological replicates) and total pSS patients (blue; *n* = 48: biological replicates) or stratified according to the absence (No HQ, *n* = 25) or the presence of hydroxychloroquine (HQ; *n* = 17) or other (OT; *n* = 6) treatments (purple).BRepresentative confocal microscopy (40× magnification) showing immunofluorescence analysis of CD1c (white), HLADR (green), and CD56 (red) expression on an SG tissue section from a representative pSS patient. Cell nuclei were stained with DAPI (blue). Cells co‐expressing CD1c and HLADR markers with close proximity to CD56 cells are highlighted with a yellow arrow.CProportions of MICa/b^+^ and ULBP1^+^ cells from circulating cDC2 (left plots), cDC1 (middle plots), and CD14^+^ Mo (right plots) in 46 HD (gray) and total *n* = 42, *n* = 15 HQ, *n* = 22 No HQ, and *n* = 5 OT pSS patients (blue and purple).DRepresentative confocal microscopy (40× magnification) showing immunofluorescence analysis of CD1c (white), HLADR (green), and MICab (red) expression on a representative SG tissue section from a pSS patient from a total of *n* = 4 individuals tested. Cell nuclei were stained with DAPI (blue). Cells co‐expressing CD1c, HLADR, and MICAB are highlighted with an orange arrow.EQuantification of proportions of CD1c^+^ HLA‐DR^+^ cDC2 detected in SMSG from *n* = 4 pSS patients that either co‐express or not MICa/b.FProportions of the CD56^hi^ CD16^+^ NK cell subset proportions (left) after 16 h culture of sorted autologous CD56^+^ NK cell in media alone or in the presence of sorted autologous circulating Mo, cDC2, and cDC1 from *n* = 8 pSS patients at ratio 1:2 (myeloid cell:NK) and expression of CD107a (right) on CD56^+^ NK cells in these functional assays is shown. Data information: (A, C, E, F): Data are represented as box and whiskers plots with maximum and minimum range and a median value central band. In panel (E), MICa/b^+^ and MICa/b^−^ cells from the same donor are paired. Statistical significance was calculated with a two‐tailed Mann Whitney (A, C) or a Wilcoxon matched pairs (E, F) tests. *P < 0.05; **P < 0.01; ***P < 0.001; ****P < 0.0001. Source data are available online for this figure.

Myeloid cells such as DCs and Mo might contribute to pSS pathology (Wildenberg *et al*, 2009; Vogelsang *et al*, 2014; Huijser *et al*, 2022), but alterations in their cell subset frequencies and their functional ability to activate NK cells have not been compared in these patients. To address this, we first evaluated by flow cytometry the frequencies of different circulating myeloid cell subsets including Lineage^−^ CD14^−^ CD11c^−^ HLADR^+^ CD123^+^ pDCs, and Lineage^−^ CD14^−^ CD11c^−^ HLADR^+^ cDC2 (CD1c^+^) and cDC1 (CD141^+^) in the PB from pSS individuals recruited to our study and compared with patterns from HD (Appendix Fig S3A). Proportions of circulating CD14^+^ CD16^−^ classical (C), CD14^+^ CD16^int^ transitional (T), and CD14^lo^ CD16^hi^ non‐classical (NC) Mo were also evaluated in individuals from our pSS and HD cohorts, respectively (Fig 2, and Appendix Fig S3A and B). Frequencies of pDCs tended to decrease but were less significantly affected in pSS patients independently of HQ treatment compared to HD (Appendix Fig S3B and Table S3). However, proportions of circulating inflammatory NC Mo were significantly increased in PB of pSS patients (Fig 2A, left). Interestingly, the increase in this population was most noticeable in untreated pSS patients or in individuals receiving OT (Fig 2A). In contrast, T and C Mo were not significantly affected in any group of pSS patients compared to HD (Appendix Fig S3C and Table S3). In contrast, proportions of cDC2 were markedly reduced in the PB of pSS individuals independently of HQ treatment, although they were not affected in the OT group (Fig 2A). Proportions of cDC1 were also lower but only significantly different in pSS patients receiving HQ compared to HD (Fig 2A). Moreover, cDC2 defined as HLADR^+^ cells with myeloid morphology and co‐expressing CD1c and negative for CD19 expression could be detected in infiltrated areas from the SGs of pSS in close proximity to CD56^+^ NK cells in this tissue (Fig 2B and Appendix Fig S3D).

To evaluate the potential of different myeloid cell subsets from pSS patients to activate NK cells, we investigated expression of ligands for activating NK receptor NKG2D such as MICa/b and ULBP1 or for inhibitory receptors such as PCNA on cDC2, cDC1, and in Mo from these individuals and in HD (Fig 2C and Appendix Fig S4A–C). Interestingly, only circulating cDC2 from pSS patients expressed significantly higher levels of ligands for NKG2D such as MICa/b and ULBP1 (Fig 2C). Once again, the upregulation of these ligands was present in cDC2 from HQ and non‐HQ pSS groups, but more significantly increased in the former group (Fig 2C). In contrast, expression of these markers was not affected in the OT group, suggesting that these types of treatments diminished alterations in myeloid cells (Fig 2C). No significant differences in the expression of these ligands were found on cDC1 and Mo from pSS patients (Appendix Fig S4A and B). Proportions of PCNA^+^ cells tended to be lower in cDC2 and cDC1 and increased in Mo (Appendix Fig S4C). In addition, expression of MICa/b was present in the SG tissue from pSS patients and was also confirmed in the majority of infiltrated CD1c^+^ HLA‐DR^+^ cDC2 (Fig 2D and E). In contrast, MICa/b expression was less frequently detected and no infiltrated cDC2 were found in non‐autoimmune salivary gland control samples (Appendix Fig S4D). Next, we tested whether Mo, cDC2, and cDC1 sorted *ex vivo* from the PB samples of pSS patients could differ in their intrinsic abilities to stimulate autologous NK cells. Notably, we observed that NK cultured with cDC2 from pSS patients induced the highest proportions of CD56^hi^ CD16^+^ cells enriched in these patients compared to baseline, in contrast to cells from HD (Fig 2F and Appendix Fig S4E, left). Proportions of CD56^dim^ CD16^+^ were also higher in the presence of cDC2 and to a lesser extent with Mo, but in this case, efficiency was similar in cells from pSS patients from HD (Appendix Fig S4E, middle and right). Moreover, phenotypical increase in CD16^+^ CD56^hi^ NK cells in response to cDC2 was accompanied by the induction of higher expression of CD107a in NK cells, and again this effect was more significant in the presence of cells from pSS (Fig 2F and Appendix Fig S4F). On the other hand, Mo displayed intermediate capacities to induce these NK phenotypical patterns, while cDC1 were unable to increase proportions of CD16^+^ CD56^hi^ NK cells or CD107a^+^ cytotoxic cells (Fig 2F). However, cDC1 from both pSS patients and HD seemed to selectively induce expression of IFNγ on NK cells (Appendix Fig S4G). Collectively, these data indicate that cDC2 from pSS patients are characterized by enhanced capabilities to induce maturation and activation of cytotoxic CD56^hi^ CD16^+^ NK cells.

### Increased inflammatory CD64 ^+^ cDC2 associates with specific innate transcriptional signatures in pSS patients

**Figure 3:**
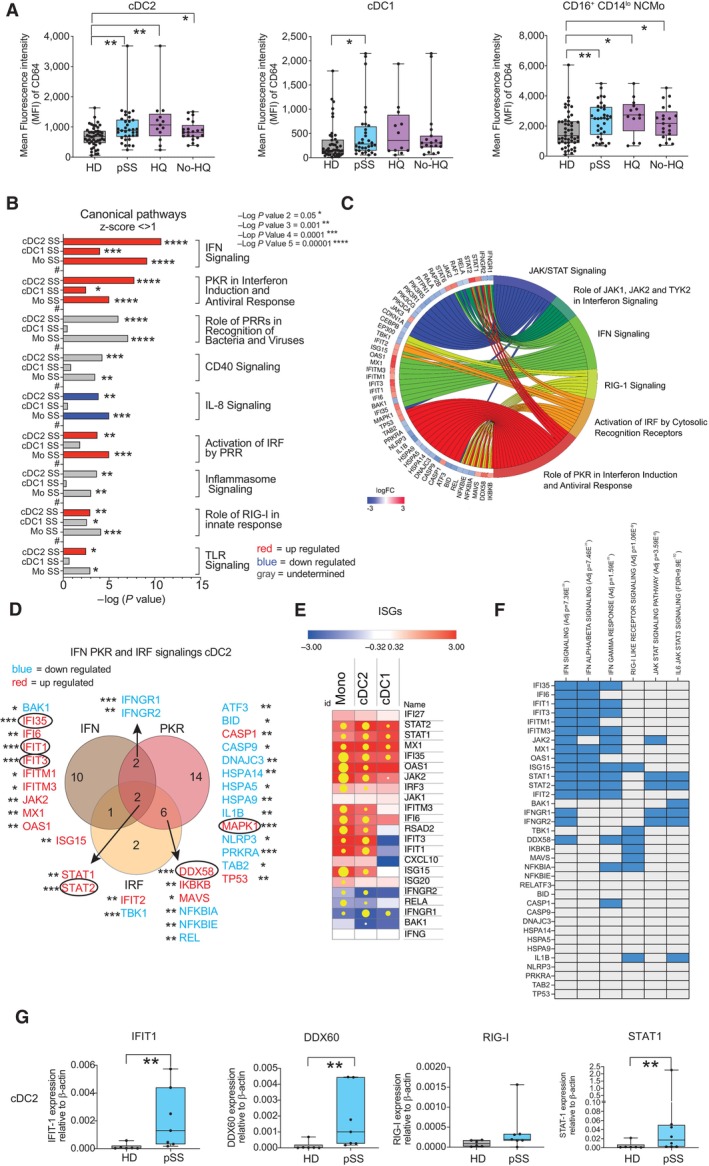
Expression of CD64 and differential transcriptional signatures in circulating Mo, cDC2 and cDC1 from pSS patients Mean of fluorescence intensity of CD64 on cDC2 (left), cDC1, non‐classical (NC) CD16^+^ CD14^lo^ Mo, and Transitional (T) CD16^hi^ CD14^++^ in the PB from HD (gray; *n* = 48) and pSS patients (blue; *n* = 34 total patients, *n* = 13 HQ, and *n* = 21 No‐HQ as biological replicates). Statistical significance was calculated using a two‐tailed Mann Whitney test. *P < 0.05; **P < 0.01.Significance of selected upregulated (positive z‐score > 1; red), downregulated (negative z‐score < −1; blue) canonical pathways predicted by Ingenuity Pathway Analysis (IPA) (full analysis shown in Appendix Table S2) from significant differentially expressed genes (DEG *P* < 0.05 after FDR correction, log2FC > 1.5 and < −1.5) in Mo, cDC2, and cDC1 from *n* = 4 pSS vs. *n* = 4 HD. Pathways that did not have a Z‐score or did not reach these mentioned value cut offs were labeled in gray.Chordplot representing overlap of selected IPA‐predicted canonical pathways associated with the indicated innate immune pathways (right area, in different colors) and the DEG included within each of them in cDC2 from pSS patients. Changes and levels in transcriptional expression of each DEG are highlighted in red (upregulation) and blue (downregulation).Venn's diagram of overlapping significant DEG included in selected IFN, PKR, and IRF canonical pathways in PB cDC2 from pSS patients. FDR‐corrected *P* values of differential transcriptional expression of indicated DEG are highlighted; *P* < 0.05; **P < 0.01; ***P < 0.001.Heatmaps representing log2‐FC in transcription of selected Interferon Stimulated genes (ISG) or transcripts associated with the IFN pathway on each cell subset from the PB from pSS vs. HD (red, upregulated; blue, downregulated). Yellow dots size is proportional to statistical significance of DEG expression (*P* < 0.05 FDR corrected values).Heatmap representing enrichment of selected DEG associated with IFN, RIG‐I, and IFN receptor pathways using Gene Set enrichment Analysis (GSEA). Overlap of each DEG with the indicated sets is highlighted in blue. FDR‐corrected p values for enrichment of each gene set are also included.qPCR validation of the indicated transcript from sorted circulating cDC2 from *n* = 7 pSS patients (blue) vs. *n* = 6 HD (gray). Statistical significance was calculated using a two‐tailed *U* Mann Whitney test. **P < 0.01. Data information: (A, G): Data are represented as box and whiskers plots with maximum and minimum range and a median value central band. Source data are available online for this figure.

We next assessed whether the observed phenotypical and functional alterations in cDC2 may associate with specific activation states and the IFN environment characteristic of pSS patients (Emamian *et al*, 2009; Hall *et al*, 2012; Bodewes *et al*, 2018, 2019). We focused on HQ and untreated pSS patients since we previously observed the most significant differences in cDC2 frequencies and in expression of NKG2D ligands. To this end, we evaluated the expression of markers previously associated with activation or response to IFN in the different myeloid subsets, such as CD64 (Bourgoin *et al*, 2020), which also identifies inflammatory cDC2 characterized by antiviral profiles (Martin‐Gayo *et al*, 2018; Bosteels *et al*, 2020). The expression of other IFN‐inducible molecules such as PD‐L1, CD40, and CD86 was also analyzed. Interestingly, CD64 expression was significantly increased in all myeloid subsets from pSS. However, among DC, cDC2 expressed higher per cell levels of this molecule than cDC1 and pDCs, suggesting an enrichment on inflammatory cDC2 (Fig 3A and Appendix Fig S5). Upregulated CD64 expression was observed in both HQ and non‐HQ pSS patients, but more significantly in the former group (Fig 3A). As expected, CD64 was highly expressed in C Mo and remained upregulated in T and NC Mo (Fig 3A and Appendix Table S3). On the other hand, less significant changes among DC and Mo subsets were observed in the expression of other IFN‐inducible markers such as CD86, CD40, and PD‐L1, although expression of some of these molecules such as CD40 was more significantly altered in pSS patients treated with HQ (Appendix Fig S5). Therefore, differential enrichment on CD64^+^ inflammatory cDC2 might reflect differences in the antiviral states or response to IFN compared to cDC1 and Mo.

To further assess whether cDC2, cDC1, and Mo in pSS patients may differ on the activation of specific antiviral or IFN‐related pathways, we analyzed by RNA‐seq the differential transcriptional patterns of these cell subsets from PB of *n* = 4 pSS patients and *n* = 4 HD. Principal Component Analysis of detected genes indicated that each subset from pSS was transcriptionally different from the corresponding HD controls (Appendix Fig S6A). A comparative gene expression analysis of each cell population between our two study populations identified a total of 988, 1,137, and 754 significant differentially expressed genes (DEG; FDR corrected *P* < 0.05, considering a cutoff log2Fold change in expression in pSS vs. HD > 1.5 and < −1.5) in Mo, cDC2, and cDC1, respectively (Appendix Table S4). Computational prediction of canonical pathway analysis by IPA predicted higher level of differential expression of genes associated with activation of IFN signaling, activation of IFN‐mediated antiviral innate responses including RIG‐I in cDC2 and Mo from pSS in comparison with the cDC1 that presented either lower significance or downregulated expression of these pathways (Fig 3B). Interestingly, most of these pathways were interconnected and we found overlapping and non‐overlapping DEG with the top upregulated IFN‐related pathways in cDC2 and Mo, in which we could identify transcripts involved in IFN receptor signaling and interferon‐stimulated genes (ISG) (Fig 3B–E). In fact, using a Gene Set Enrichment Analysis (GSEA) approach, we were able to identify groups of genes associated with signaling upstream or downstream type I and type II IFNs (Fig 3F). In this regard, while RIG‐I (DDX58) and its positive modulator DDX60 (Oshiumi *et al*, 2015) helicases were highly upregulated on both cDC2 and Mo from pSS patients, we observed that the DEG more significantly upregulated in cDC2 included interferon‐stimulated genes (ISG) such as IFIT1 and IFIT3, BST2, MS4A4A, STAT1, and STAT2, and these molecules were more frequently associated with IFN I and less enriched on IFN II signaling pathways in our GSEA analysis (Fig 3C–F, and Appendix Fig S6B and C). Among these, IFIT1 is a downstream effector of the RIG‐I pathway (Yap *et al*, 2020) and its expression associates with expression of ligands for NK cell receptors (Idso *et al*, 2020). STAT1 and STAT2 are ISG that overlapped with all the IFN‐related pathways analyzed in both cDC2 and Mo and are required for the signaling of IFN I and IFN II receptors (IFNAR and IFNGR, respectively) (Michalska *et al*, 2018) (Fig 3D and E, and Appendix Fig S6B and C). In contrast, IFN‐related or ISG DEG more significantly upregulated in Mo included ISG15, OAS1, IFITM3, IFI6, JAK2, IFI35, MAVS, and IRF3 (Fig 3E). In contrast, activation of IRF by PRR was more upregulated on Mo from pSS. Alternative innate pathways such as the inflammasome were less significantly affected in both cDC2, cDC1, and Mo from pSS (Fig 3B). Importantly, a more consistent and significant increase of IFIT1 transcription was confirmed in cDC2 from pSS compared to HD, while this was more variable in Mo (Fig 3G and Appendix Fig S6D). Remarkably, qPCR analysis indicated a weak induction on the expression of RIG‐I but a significant increase in its positive modulator DDX60 (Fig 3F). In addition, we also validated significant upregulation of STAT1 in both cDC2 and Mo from pSS (Fig 3G and Appendix Fig S6D). Thus, cDC2 from pSS are characterized by an enrichment in inflammatory cDC2 phenotype and the selective activation of ISGs specifically associated with targets and modulators of the antiviral RIG‐I and IFN receptor pathways.

### The RIG‐I/DDX60‐IFNAR axis regulates expression of ligands for NK receptors in primary cDC2

**Figure 4:**
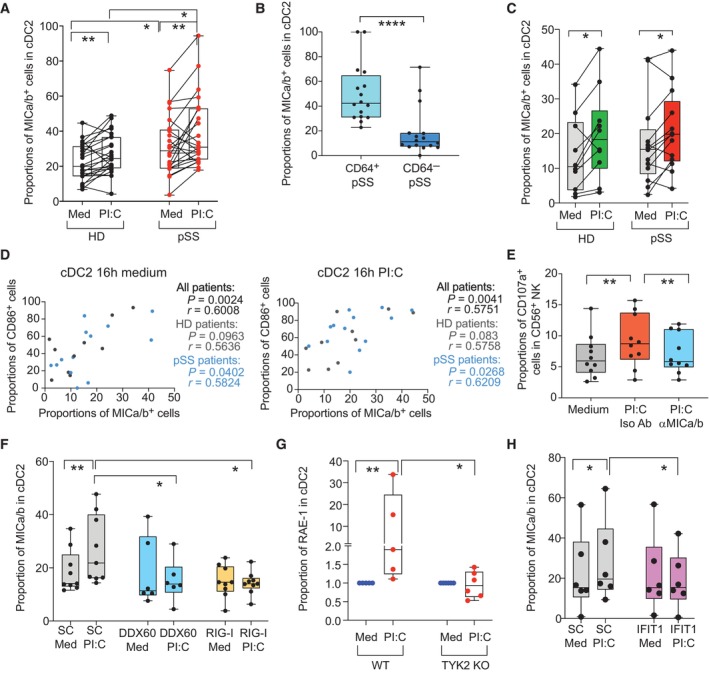
Induction of MICa/b in response to poly I:C and dependency on RIG‐I, DDX60, IFIT1, and IFNAR in primary human cDC2 A–CProportions of MICa/b^+^ cells in cDC2 from HD and pSS in PBMC (A) or preisolated cells (C) after 16 h poly I:C (PI:C) stimulation in cDC2 isolated from the blood of HD (biological replicates *n* = 24, A and *n* = 10, C) and pSS patients (biological replicates *n* = 24 A; *n* = 11 C). (B): Proportions of MICa/b^+^ cells on gated CD64^+^ and CD64^−^ subpopulations included in CD11c^+^ CD14^−^ cDC from PBMC of *n* = 16 pSS patients.DSpearman correlations between proportions of CD86^+^ and MICa/b^+^ cells on cDC2 from HD and pSS after 16 h culture in media alone (left) or in the presence of poly I:C (right). P and R values of all combined cohorts or each individual study group are shown.EProportions of CD107a^+^ NK cells after culture in media alone or with autologous poly I:C stimulated cDC2 in the presence of either anti‐MICa/b or an isotypic control Abs (*n* = 10 biological replicates from pSS patients).F–HSurface expression of MICa/b on isolated primary cDC2 from HD biological nucleofected with either DDX60 (*n* = 6 biological replicates) or RIG‐I (*n* = 9 biological replicates) (F) or IFIT1 (H) specific siRNAs or control scramble (SC; biological replicates *n* = 9 for F and *n* = 6 for H) siRNAs after 16 h in the presence of media or poly I:C (PI:C). (G): Fold change in RAE‐I expression in cDC2 from the spleen of wild type (WT) vs. Tyk2 knock out (KO) mice exposed to poly I:C compared to cells cultured in media. Data information: (A, B, C, E, F, G, H): Data are represented as box and whiskers plots with maximum and minimum range and a median value central band. In panel (A and C), data from the same donor in media or poly I:C treatments have been paired in (A) and (C) panels. Statistical significance (A–H) was calculated using two‐tailed Wilcoxon matched pairs signed rank (A, C, E, F, G, H) and Mann Whitney tests (A, G) to compare changes under different conditions or between independent experimental groups, respectively (*P < 0.05; **P < 0.01; ****P < 0.0001). Source data are available online for this figure.

Our previous transcriptional analyses revealed that activation of RNA sensing pathways including RIG‐I or IFN receptor signaling could be linked with transcriptional IFN signatures present in inflammatory cDC2 from pSS patients. To confirm whether innate sensing of RNA could be involved in the expression of ligands for NK receptor on cDC2, we exposed cells from pSS patients and HD to the RIG‐I/MDA‐5/TLR3 agonist poly I:C. As shown in Fig 4A, MICa/b was significantly induced in cDC2 within PBMC from pSS patients and in HD in response to poly I:C. Notably, inflammatory CD64^+^ cDC2 induced by poly I:C expressed higher levels of MICa/b than CD64^−^ cells (Fig 4B and Appendix Fig S7A). In addition, a modest and less significant increase of MICa/b expression was also observed in Mo from pSS compared to HD (Appendix Fig S7B). Since expression of MICa/b could also be dependent on other cell types or the response to cytokines in PBMC, we further investigated the regulation of this molecule in pre‐isolated cDC2 from HD and pSS. Upregulation of MICa/b was confirmed on isolated cDC2 from pSS and HD after 16 h in the presence of poly I:C compared to cells cultured in media (Fig 4C). In line with a connection between expression of MICa/b and the activation status of cDC2, we observed a significant correlation between expression levels of MICa/b and CD86 both at baseline and after poly I:C stimulation in cDC2 from both pSS patients and HD (Fig 4D). Thus, detection of dsRNA in cDC2 regulates the expression of ligands for activating NK receptors. Importantly, we observed that expression of CD107a on NK cells from pSS patients cocultured with autologous cDC2 stimulated with poly I:C was significantly reduced in the presence of blocking antibodies directed to MICa/b compared to isotypic Abs (Fig 4E). Notably, these interactions were not translated in the selective killing of poly I:C treated cDC2 in the presence of NK cells (Appendix Fig S7C). We also asked whether other factors such as IL‐15, previously involved in DC‐NK cell crosstalk (Lucas *et al*, 2007; Finch *et al*, 2018), could also account for the increased ability of poly I:C treated cDC2 to activate NK cells. However, we did not observe any significant differences in IL‐15 transcripts in cDC2 from pSS or HD exposed to poly I:C compared to baseline (Appendix Fig S7D). Likewise, we did not find significant differences in frequencies of CD107a^+^ NK cells exposed to cDC2 in the presence of neutralizing antibodies against IL‐15 (Appendix Fig S7E). Also, we confirmed that poly I:C alone did not induce an increase in CD107a or IFNγ on pre‐isolated NK cells and cultured individually *in vitro* (Appendix Fig S7F).

Finally, to further ascertain which nucleic acid sensors may be responsible for the observed modulation of MICa/b induced by poly I:C, we first looked at our transcriptional dataset to evaluate whether expression of a broader number of innate RNA sensors might be altered in cDC2 from pSS patients. While we had previously observed upregulation of the RNA helicases RIG‐I and DDX60 in pSS (Appendix Figs S6B and S8A), no significant changes on the transcription of TLR3 and TLR8 or MDA5 or alternative helicases DDX59, DDX18, DDX24, DDX21, and DDX6 were detected in cDC2 from pSS patients (Appendix Fig S8A). In contrast, transcriptional levels of DNA sensors capable of inducing type I IFN responses such as cGAS or STING were weakly or not significantly affected in cDC2, cDC1, and Mo from pSS patients (Appendix Fig S8B).

Consequently, we focused on determining whether induction of NK receptor ligands in response to poly I:C on cDC2 could be dependent on expression of RIG‐I and DDX60, the downstream signaling through IFN receptors or the expression of specific ISG such as IFIT1. To assess this possibility, we performed siRNA‐mediated knockdown of either of these sensors, STAT1 or IFIT1, on cDC2 isolated from the PB of HD. After confirming specific siRNA silencing by RT–qPCR (Appendix Fig S8C, E and F), we compared the expression of MICa/b in siRNA treated cDCs after poly I:C stimulation with control cells nucleofected with scramble siRNAs. As shown in Fig 4F, induction of MICa/b on cDC2 in response to poly I:C was significantly abrogated after DDX60 knock down, while cells defective for RIG‐I displayed the lowest levels of this ligand at baseline and were not able to induce its expression upon poly I:C exposure, confirming that these upstream RNA sensing pathways are involved in the regulation of the ligand. Similarly, upregulation of CD86 and CD40 expression after poly I:C stimulation was less evident on cDC2, transfected with RIG‐I specific siRNAs, while DDX60 knockdown cells were able to acquire maturation markers in response to stimulation (Appendix Fig S8D). In order to determine whether signaling downstream the RIG‐I/DDX60 pathway was required for induction of MICa/b, we also knocked down the expression of STAT1, which is required for both IFNAR and IFNGR signaling (Michalska *et al*, 2018) and IFIT1, a known ISG which potentiates RIG‐I signaling pathway (John *et al*, 2018). We observed that siRNA‐mediated knockdown of STAT1 significantly impaired the upregulation of MICa/b expression on cDC2 exposed to poly I:C, indicating that signaling through either IFNAR or IFNGR is required for this process (Appendix Fig S8G). In order to elucidate whether signaling of IFN I or II receptors was involved in the regulation of NKG2D ligands, we took advantage of TYK2 knockout mice, since this molecule is required for signaling of IFNAR but not IFNGR (Velazquez *et al*, 1992; Platanias, 2005). Therefore, we evaluated changes in expression of RAE‐I, the murine homolog of MICa/b, in isolated cDC2 from Wt or TYK2‐deficient mice at baseline and after poly I:C stimulation. As shown in Fig 4G, cDC2 from TYK2 mice were unable to increase RAE‐I expression after exposure to poly I:C, in contrast to cells from Wt mice, indicating that IFNAR is involved in the regulation of the ligand. Also, si‐RNA‐mediated knockout of the ISG IFIT1 also significantly prevented MICa/b upregulation in cDC2 exposed to poly I:C (Fig 4H), supporting that IFNAR and the RIG‐I pathways are both relevant. Together, our data indicate that expression of MICa/b on cDC2 is regulated by RIG‐I/DDX60 and IFNAR in response to poly I:C, contributing to the ability of these cells to activate cytotoxic NK cells.

### Increased NKG2D^hi^ CD11b ^+^ CD27 ^+^ NK cells and CD64 ^+^ RAE‐I ^+^ cDC2 in the salivary gland *in vivo* after exposure to poly I:C.

**Figure 5:**
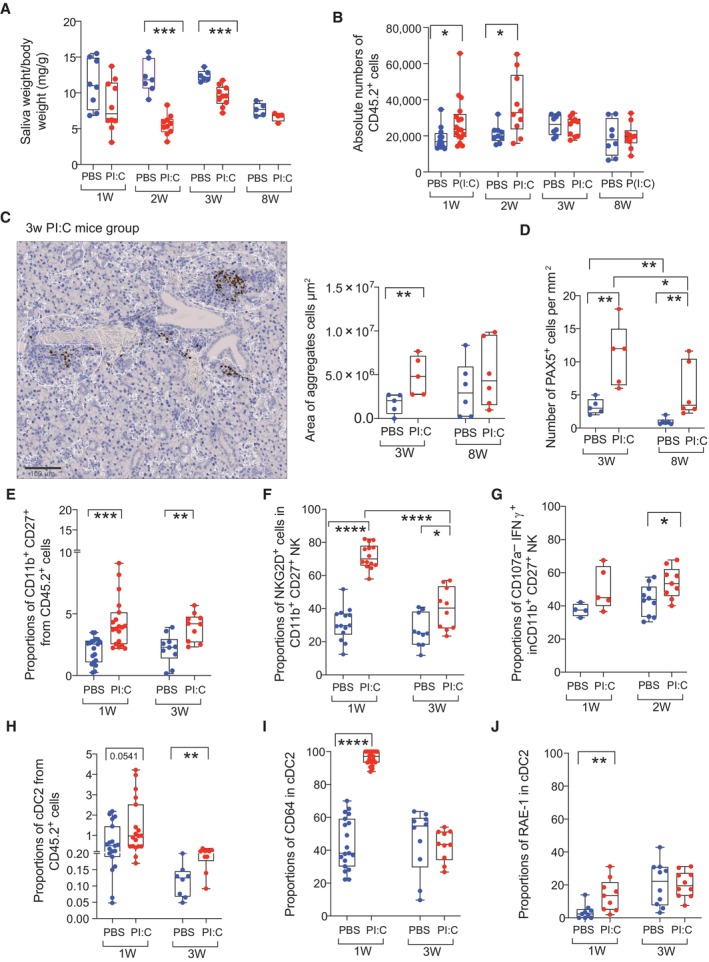
*In vivo* recruitment of activated NK cells and cDC2 to the SMSG after injection of mice with poly I:C AAnalysis of salivary gland function measured as saliva weight/body weight after Pilocarpine injection in mice treated with PBS (blue; biological replicates *n* = 8; *n* = 7; *n* = 6; *n* = 5) or poly I:C (red; biological replicates *n* = 10; *n* = 10; *n* = 10; *n* = 4) at 1, 2, 3, 8 weeks post injection, respectively.BAbsolute numbers of hematopoietic CD45.2 cells detected on homogenized submandibular salivary gland (SMSG) from mice injected with PBS (blue; biological replicates *n* = 19; *n* = 9; *n* = 8) and poly I:C (red; biological replicates *n* = 22, *n* = 10, *n* = 10; *n* = 10).C, DImage of histological analysis of immune cell infiltrates and Pax5^+^ cells identifying B cells in SG from a representative mouse injected with poly I:C (C, left). Scale bar: 100 μm. Quantification of area of total immune infiltrates (C, right) and number of Pax5^+^ cells (D) detected in the SMSG from mice‐treated PBS (blue; biological replicates *n* = 5 and *n* = 6 for 3 and 8 weeks, respectively) or poly I:C (red; biological replicates, *n* = 5 and *n* = 6 for 3 and 8 weeks, respectively) is shown.E–GFrequencies of CD11b^+^CD27^+^ NK1.1^+^ CD3^−^ NK cells (E) and proportions of NKG2D^+^ (F) and CD107a^−^ IFNγ^+^ (G) cells in this population in the SMSG of mice after 1 and 3 weeks after injection with PBS (blue; biological replicates *n* = 19 in E, F and *n* = 4 in G for week 1; *n* = 10 in E, F for week 3 and for week 2 in G) or poly I:C (red; biological replicates *n* = 19 in E, F and *n* = 5 in G for week 1; *n* = 10 in E–G for week 3).H–JProportions of CD11b^+^ CD11c^hi^ cDC2 (H), expression of CD64 (I) and RAE‐I (J) in this population infiltrated in the SMSG from mice after 1 and 3 weeks after injection with PBS (blue; biological replicates *n* = 19 in H, I and *n* = 9 in J for week 1; *n* = 8 in I and *n* = 10 in I, J for 3 weeks) and poly I:C (red; biological replicates *n* = 19 in H, I and *n* = 9 in J for week 1; *n* = 10 in H–J for week 3). Data from three independent experiments are included with biological replicates. Data information: (A–J) Data are represented as box and whiskers plots with maximum and minimum range and a median value central band Statistical significance was calculated with a two‐tailed Mann Whitney test. *P < 0.05; **P < 0.01; ***P < 0.001; ****P < 0.0001. Source data are available online for this figure.

To determine whether *in vivo* activation of RIG‐I/IFN responses could recapitulate some of the phenotypical changes and activation patterns on specific subsets of NK cells and on cDC2 previously observed in pSS and suggest the involvement of these cells in pathologic inflammation in the submandibular SG (SMSG), we used a murine *in vivo* model induced by poly I:C inoculation for 2 weeks which has been shown to induce early immune cell infiltration and subsequent SMSG dysfunction at later time points (Nandula *et al*, 2011, 2013) (Appendix Fig S9A). Using this model, we observed a significant hypofunction of the SG from 1 to 3 weeks post poly I:C injection, and similar trends were observed up to 8 weeks post treatment (Fig 5A). Interestingly, while only a significant increase of a first wave of hematopoietic CD45.2^+^ cell infiltration into the SMSG was observed in mice injected with poly I:C compared to those receiving PBS at early times points (1 and 2 weeks), aggregates of immune cells were still evident at 3 and to some extent 8 weeks p.i. (Fig 5B and C). Interestingly, immune cells infiltrated at late time points (3 and 8 weeks p.i.) were observed by the presence of Pax5^+^ B cells present in aggregates in the SMSG (Fig 5D). Thus, although this animal model was not based on the priming of antigen‐specific autoreactive adaptive immune cells, activation of RIG‐I/IFN *in vivo* leads to potentially pathogenic inflammatory adaptive immune cell profiles in the SMSG that are similar to those present in pSS patients. We then focused on analyzing frequencies and phenotype of murine NK and cDC2 cells in the SG of poly I:C injected mice. NK cell subsets were defined as NK1.1^+^ CD3^−^ cells present in CD45^+^ infiltrates present in the SMSG and differing in expression of CD27 vs. CD11b (Appendix Fig S9B). Interestingly, frequencies and absolute numbers of CD11b^+^ CD27^+^ NK cells, equivalent to human CD56^hi^ CD16^+^ cells enriched in pSS patients, were increased after poly I:C injection in infiltrates present in the SMSG at 1 week after initiating poly I:C injections and remained increased at 3 weeks even when treatment been interrupted (Fig 5E and Appendix Fig S9C). Besides, frequencies of CD11b^+^ CD27^+^ NK cells expressing high levels of NKG2D were increased in the SMSG from poly I:C injected mice at all times analyzed (Fig 5F), also resembling phenotypic characteristics of NK cells from pSS patients previously observed (Appendix Fig S2). In addition, murine CD11b^+^ CD27^+^ NK cells from the SMSG rather than PB from mice injected with poly I:C contained significantly higher proportions of IFNγ^+^ CD107a^−^ cells compared to control animals at 2 weeks post treatment (Fig 5G and Appendix Fig S9D). In contrast, CD107a^+^ IFNγ^+^ cells were significantly increased in CD11b^+^ CD27^+^ NK cells from SMSG compared to cells from PB at baseline, and they only increased in the PB but not in cells from the tissue after poly I:C treatment (Appendix Fig S9E). Thus, these data suggest the preferential recruitment of activated CD11b^+^ CD27^+^ NK cell subset in the SMSG *in vivo* after poly I:C treatment.

In addition, we also observed a significant increase in the proportions and numbers of Ly6C^−^ CD11c^hi^ CD11b^+^ cDC2 in CD45.2^+^ cells in the SMSG from poly I:C group at 1 and 3 weeks after treatment initiation (Fig 5H, and Appendix Fig S10A and B) and a significant increase levels of CD64 and RAE‐I was observed in these cells specifically at 1 week post injection (Fig 5I and J), consistent with the dependence of these markers on the IFN response induced by poly I:C. In contrast, numbers and proportions of XCR1^+^ cDC1 and Ly6C^+^ Mo were either markedly reduced or not significantly changed in the SMSG from poly I:C injected mouse group (Appendix Fig S10B and C). Moreover, no significant differences in expression of CD64 or RAE‐I were found in cDC1 and Mo from poly I:C treated mice compared with PBS treated controls (Appendix Fig S10D and E). In conclusion, induction of RNA sensing/IFN response *in vivo* induces enrichment and specific patterns of activation on NKG2D^+^ CD11b^+^ CD27^+^ NK cell and CD64^+^ RAE‐I^+^ cDC2 in the salivary gland that are similar to phenotypical characteristics present in pSS patients.

## Discussion

Our study identifies a CD56^hi^ CD16^+^ subset enriched on circulating NK cells from pSS patients which associates with higher cytotoxic function *ex vivo* and co‐expression of high levels of granzyme B on NK cells infiltrated in SGs from these patients. Our data support that the CD56^hi^ CD16^+^ NK cells may directly perform a cytotoxic function but also serve as a precursor of more mature CD56^dim^ CD16^+^ NK cells, which also display an activated phenotype in pSS patients. These findings expand current knowledge supporting increased activation of NK cells and increased CD56^hi^ cells in pSS patients and their potential contribution to gland tissue damage (Christodoulou *et al*, 2010; Rusakiewicz *et al*, 2013; Ming *et al*, 2020). Additionally, NK cells from pSS subjects are characterized by increased expression of the activating receptor NKG2D providing new biomarkers for activated NK cells in pSS, which might explain higher susceptibility of these cells to become activated and mediate cytotoxic function. NKp30 also tended to be more expressed in NK from pSS in agreement with previous studies (Rusakiewicz *et al*, 2013), and may also contribute to cytotoxic NK cell activity. Interestingly, type I and II IFN have been suggested to have opposite effects inducing and repressing NKG2D expression, respectively (Zhang *et al*, 2005; Burgess *et al*, 2008). It is therefore conceivable that increased levels of NKG2D might be the result of secondary response to specific IFN types environment present in pSS. Importantly, increased activation of NK cells and the presence of IFN environments have been suggested to have an impact in subsequent autoimmune adaptive immune responses, such as the induction of anti‐RO/SSA and La/SSB autoantibodies (Ivanchenko *et al*, 2021). In line with this possibility, we have observed higher levels of expression of granzyme B on NK cells infiltrated in damaged areas of the SMSG in pSS patients. Also, TRAIL^+^ NK cells have been proposed to eliminate CD4^+^ T cells in a model of CMV‐induced autoimmunity (Schuster *et al*, 2014).

Alternatively, we provide novel evidence of the potential participation of inflammatory CD64^+^ cDC2 as a potential cellular mechanism driving the activation of NK cells in pSS.

In fact, our data indicate that cDC2 from pSS patients are characterized by specific IFN signatures that are associated with high basal abilities to support NK cell maturation *in vitro* and higher levels of the canonical NKG2D ligand MICa/b which is also highly expressed in cells infiltrated in the SG. We also demonstrate that poly I:C stimulation also induces MICa/b expression in inflammatory cDC2 *in vitro*. Importantly, we have provided evidence about the *in vivo* infiltration of inflammatory CD64^+^ cDC2 and activated NK cells into the SMSG in a mouse model of pSS induced by poly I:C, in agreement with previous studies describing the infiltration of CD11c^+^ cDCs in this tissue (Ozaki *et al*, 2010). Moreover, we provided data confirming that phenotypical alterations previously observed in cDC and NK from pSS patients, such as the upregulation of CD64 and NKG2D ligands in CD11b^+^ cDC2 and the enrichment on CD11b^+^ CD27^+^ NK cells displaying high expression of NKG2D, also occur *in vivo* in the salivary gland after stimulating RNA sensors and IFN pathway *in vivo*. Our data and previous studies in our or similar *in vivo* models (Nandula *et al*, 2013; Killian *et al*, 2021) support the importance of the IFN response and the crosstalk between cDCs and NK cells in the SG during the onset of pSS. However, potential alterations of other innate immune subsets such as innate like cells (ILC) was not addressed in our *in vivo* or *in vitro* studies. In particular, ILC1 also co‐express NK1.1, can be found in the salivary gland (Jiao *et al*, 2016), and correlate with disease activity in pSS patients (Blokland *et al*, 2019; Kawka *et al*, 2023). Therefore, future studies of ILC in the poly I:C *in vivo* model and how potential changes in ILC1 in this model are associated to cDC2 and NK should be conducted. In addition, the contribution of NK1‐1^+^ CD3^+^ NKT cells, previously reported to be downregulated in the PB of pSS patients (Zhou *et al*, 2022) were not directly studied *in vivo* in response to poly I:C injection. However, our study and others indicate that these cells represent a minority of the NK1.1^+^ present in the SG at baseline and after poly I:C injection or CMV infection (Tessmer *et al*, 2011), suggesting a limited role of these cells in our *in vivo* model. Therefore, although the *in vivo* model used in our study does not strictly reproduce an antigen‐specific autoimmune response, we have shown that triggering the TLR3/RIG‐I/Pathway *in vivo* recapitulates some of the aberrant activation of innate immune cells characteristic of pSS. Interestingly, we have observed that pSS patients receiving HQ are characterized by more significant differences in the expression of NKG2D on NK cells and CD64, MICab, and other ISG such as PDL‐1, CD86, and CD40 on cDC2. These findings contrast with some studies that suggested that HQ may inhibit IFN signaling (Bodewes *et al*, 2020). Instead, our results may be explained by an enrichment in patients with a higher disease activity in the HQ pSS group. However, HQ treatment has been described to downregulate IL‐1β, IL‐6, and other proinflammatory cytokines that may interfere with IFN signaling (Schrezenmeier & Dörner, 2020). On the one hand, we have showed that OT group characterized by more aggressive treatments may more efficiently restore frequencies and activation profiles of myeloid cells in severe pSS patients, which suggest that it may be more effective than weaker immune‐suppressors such as HQ controlling inflammation in these subjects in line with an improvement in some clinical parameters observed. Nevertheless, future studies should address these possibilities. We have also demonstrated that induction of MICa/b on cDC2 in response to poly I:C seems to be largely dependent on the expression of RIG‐I and DDX60 on these cells, and enables cDCs to activate cytotoxic NK cells *in vitro*, providing a mechanism of aberrant killing. However, to discard the participation of alternative RNA sensors, future studies of MICa/b regulation should also be performed using specific agonists of RIG‐I instead of poly I:C. Nevertheless, our data suggest that MICa/b‐NKG2D interaction might be required for the activation of NK by DC, independent of other mechanisms such as cytokines. In this regard, IL‐15 produced by DC has been involved in maturation of NK cells (Lucas *et al*, 2007; Finch *et al*, 2018). However, our data indicate that IL‐15‐IL‐15R signaling is not a main driver on poly I:C stimulated cDC2‐NK crosstalk. However, the possible involvement of this cytokine should be further investigated in cDC2 from pSS patients, which may be able to differentially induce this cytokine in these patients, or in other subsets such as cDC1, which have been shown to efficiently produce IL‐15 (Ghilas *et al*, 2021). These data are particularly relevant since high MICa/b expression has been observed in SGs from pSS (Schrambach *et al*, 2007) and polymorphisms on MICa/b have been linked to higher susceptibility to develop Sjögren's syndrome and with higher levels of soluble MICa/b in the plasma of these patients (Carapito *et al*, 2017). In addition, we have shown that signaling through IFN receptors mediated by STAT1 is involved in regulation of MICa/b expression on cDC2, and results from TYK2‐deficient mice indicate that signaling though IFNAR is required for the induction of NKG2D ligands. Supporting this view, we have shown that induction of MICa/b also occurs on isolated cDC2 in the absence of other cells. In addition, NK cells are not capable of responding to poly I:C and are not likely to directly contribute to IFN secretion prior to cDC2 activation. Moreover, while increased expression of RIG‐I (DDX58) was also observed in Mo from pSS patients, we have described that IFIT1, which has been reported to collaborate and be a target gene of the RIG‐I pathway, is also more significantly increased in cDC2 compared to Mo (Johnson *et al*, 2018; Yap *et al*, 2020). In fact, IFIT1 has been proposed as a positive regulator of transcriptional complexes inducing the expression of ISGs in myeloid cells (John *et al*, 2018). Therefore, this factor could also contribute to higher signal transduction of the RIG‐I pathway in cDCs from pSS individuals. Consistently, induction of NKG2D ligands by TLR ligands and RNA virus infection has been previously reported (Ebihara *et al*, 2007). In addition, other innate DNA sensing pathways mediated by STING may be contributing to Mo activation in pSS (Huijser *et al*, 2022). A relevant question is the source of the IFN responsible for the transcriptional signatures observed in cDC2 from pSS. We have shown that in the absence of PBMC, cDC2 can respond to poly I:C stimulation and induce expression of MICa/b and some of the ISG identified, suggesting an autocrine signaling after IFN I production. However, although we have shown that NK cells are not able to respond to trigger IFN in response to dsRNA, the IFNγ produced by these cells and CD4^+^ T lymphocytes may also contribute to the IFN environment in pSS and influence cDC2. Therefore, the requirement of IFNGR should be investigated in more detail.

There are some limitations of our study that need to be discussed. On one hand, we have not directly addressed whether higher membrane NKG2D levels in NK cells from pSS patients are due to higher basal gene expression on these cells or could be due to alterations on the membrane trafficking of this receptor. However, further studies directly addressing the regulation of this receptor in NK cells from pSS are needed. In addition, NKG2D^+^ T cells have been recently associated with inflammatory autoimmune disorders (Ghilas *et al*, 2021) and should also be investigated in pSS.

In addition, while our *in vitro* data suggest that NK cell may reprogram cDC2 after becoming activated via NKG2D, the mechanisms by which NK cells affect the induction of adaptive immune cells in mice injected with poly I:C is still lacking and additional future studies are needed to address whether this is a direct or is the result of indirect interactions with other immune cell populations, including cDCs. Depletion of cDC *in vivo* may also help us to better understand their impact on the development of the disease. A remaining question is whether CD64 upregulation on cDC2 might be linked to the differentiation of DC from Mo, which constitutively express this receptor. While we have shown that Mo might differentiate into CD11c^lo^ cells in the murine model of SS, we also observed CD64 upregulation in other DC subtypes, suggesting this effect might actually be linked to response to IFN and the generation of inflammatory cDC2. However, further studies should address these questions in more detail. In addition, Mo from pSS also displayed upregulation of some ISGs associated with RIG‐I, and therefore, further studies are required to determine whether RIG‐I and its related downstream genes also play a role in the functionality of Mo in pSS. In addition, our data support a more effective upregulation of MICa/b on cDC2 than in Mo, which express lower levels of this molecule in response to poly I:C and are less effective inducing activation of autologous NK cells. These findings are compatible with previous studies on MoDC able to induce high levels of MICa/b expression upon poly I:C stimulation (Ebihara *et al*, 2007), although technical differences and the use of primary cDC2 in our study may explain differences in the magnitude of increase in MICa/b surface levels. However, alternative mechanisms regulating MICa/b surface levels in cDC2 should also be explored since we did not find significant different in transcriptional levels of this molecules in our RNA‐seq study that could account for the increased protein membrane levels observed *ex vivo*. Moreover, NC Mo are also enriched in pSS patients and have been previously involved in autoimmunity (Puchner *et al*, 2018), and their contribution to other pathogenic environment should be analyzed in more detail in future studies.

Another question is whether the higher basal expression of these innate sensors in cDC2 is due to intrinsic or response to IFNs, which has not been investigated in our study. In addition, while frequencies of cDC1 are also very significantly depleted from the blood and potentially migrating to SGs in pSS patients, we have not studied IFN‐independent mechanisms by which these cells might also be contributing to this pathology. As we showed previously, some of our data suggest that they might provide additional non‐redundant signals to NK cells other than the ones provided to cDC2. Both cDC subsets are known to collaborate in NK cells activation (Ferlazzo & Morandi, 2014). In fact, our data show that cDC1 might be more effective inducing IFNγ expression on NK cells, in agreement with recent studies (Pallazola *et al*, 2021; Hernández‐García *et al*, 2022). Also, cDC1 are known to activate CD8^+^ T cells, which have been previously involved in pSS pathology (Thom *et al*, 2015; Gao *et al*, 2019). In addition, we have not investigated whether pDCs could be also participating in this process, and whether increased levels of IFN produced by these cells (Båve *et al*, 2005; Lövgren *et al*, 2006) might trigger the activation of cDCs. Previously, it has been suggested that IFN responses might associate with recruitment of pDCs to the SG (Gottenberg *et al*, 2006; van der Sluis *et al*, 2020). Also, it is well established that pDCs regulate maturation of NK cells during viral infections (Golsaz‐Shirazi *et al*, 2018) and the activation of tolerogenic cells such as Treg (Martin‐Gayo *et al*, 2010; van der Sluis *et al*, 2020) that might also impact the course of the disease. On the other hand, NC Mo were significantly increased in PB of pSS patients, in agreement with previous studies (Wildenberg *et al*, 2009), and may also participate in tissue damage as previously suggested in other autoimmune diseases (Puchner *et al*, 2018). Therefore, complex interactions between different DC and Mo subsets contributing to pathology in pSS patients deserve further investigation. An important question that we did not address in our study is the nature of the danger signal or DAMPs causing aberrant activation of cDC2 *in vivo*. Our transcriptional analysis identified RIG‐I as one of the most significant pathways operating in these cells. We also have shown that expression of DDX60 is increased in cDC2 from pSS and that this molecule might play a role modulating the activity of RIG‐I as previously reported (Oshiumi *et al*, 2015). Supporting this possibility, siRNA‐mediated silencing of DDX60 seemed to have a less significant and partial impact on the function of cDCs as compared to cells lacking expression of RIG‐I. On the other hand, we did not rule out the involvement of other innate sensors that could also induce IFN and the expression of NKG2D ligands in these cells, such as ADAR (Samuel, 2001), which represents a limitation of our study. Supporting the involvement of intracellular RNA sensors as critical participants in pSS, previous studies demonstrated that RIG‐I and MDA5 can be detected *in vivo* in SGs from pSS patients (Hall *et al*, 2012; Maria *et al*, 2017). Moreover, pSS‐like syndrome can be induced *in vivo* in murine models by injection of poly I:C, a substrate to TLR3/8 and multiple intracellular RNA sensors, including RIG‐I and DDX60. Although there is a number of different animal models recapitulating some aspects of pSS pathology, including poly I:C injection, genetic mutations (FASL), and CMV infection or stimulation with autoantigens (Fleck *et al*, 1998; Scofield *et al*, 2005; Nandula *et al*, 2013), we decided to use the poly I:C‐based animal model for our study since it has been shown to induce salivary gland dysfunction and tissue damage (Nandula *et al*, 2011, 2013) and is based on the activation of the TLR3‐RIG‐I/IFN pathways, which we primarily observed in cDC2 from pSS patients. While the poly I:C potential systemic effects are not completely recapitulating all aspects of pSS pathology, this model has allowed us to evaluate the associations between the innate activation of cDC2 and the activation of NK cells in the SMSG. It is tempting to speculate that Poly I:C‐induced inflammation and immune infiltration in the tissue may lead to damage and release of DAMPs that could promote to some extent disruption of tolerance in this model (Hu *et al*, 2021). Nevertheless, the contribution of cDC2 to pSS pathology should also be confirmed in additional *in vivo* models.

Despite these limitations, our study provides new cellular and mechanistic data to better understand the immunopathology of pSS and identifies NK cells as key player in the pathology and MICab expressing inflammatory cDC2 as the mechanisms driving their activation. Moreover, our study provides novel and interesting data regarding the communication between NK cell and cDC2 which may be critical for the development of pathogenic adaptive immunity during pSS disease. Therefore, NK cells may contribute by indirect and direct mechanisms to pSS pathology by reprogramming of cDC2 and contributing to inflammation or by directly damaging salivary gland epithelia expressing MICa/b ligands (Schrambach *et al*, 2007), respectively. Thus, these findings may open the door to identify potential new cell targets for future treatments. In fact, TBK‐1, a downstream effector of RIG‐I and other innate sensors, have been identified as a potential therapeutic target for pSS (Bodewes *et al*, 2018). Collectively, future strategies targeting NK cells and RIG‐I downstream effectors on cDC2 might be promising therapeutic candidates to treat pSS.

## Materials and Methods

### Study participants

The current study included *n* = 48 Sjögren's syndrome (pSS) and *n* = 56 healthy donors (HD). Clinical characteristics and treatment regimens are summarized in Appendix Table S1. All pSS patients assessed in a single cross‐sectional visit and all of them fulfilled the 2016 ACR‐EULAR classification criteria (Shiboski *et al*, 2017). Clinical, therapeutic, and laboratory data are recorded and included in an electronic database. Biological samples were collected at the visit and stored at the Instituto de Investigación Sanitaria La Princesa (IIS‐IP) Biobank. Recruited pSS patients were classified according to the European League Against Rheumatism (EULAR) SS disease activity index (ESSDAI) (Seror *et al*, 2015, 2016). HD samples were obtained from Buffy coats obtained from the Centro de Transfusiones Comunidad de Madrid and from the Immunology Unit at Hospital Universitario de la Princesa and were used as controls for comparison purposes. No blinding of patient samples was performed. Results obtained from samples in which technical issues, low cell viability or lack of quality controls occured during the experiments were removed from the analysis.

### Ethics statement

All subjects participating in the study gave written informed consent, and the study was approved by the Institutional Review Board of Hospital Universitario de La Princesa (Protocol Number #3515) and following the Helsinki declaration. For *in vivo* experiments, mice were housed at the animal facility from Centro Nacional de Investigaciones Cardiovasculares in accordance with the institution's animal care standards. Animal experiments were reviewed and approved by the local ethics committee and were in agreement with the EU Directive 86/609/EEC, Recommendation 2007/526/EC, and Real Decreto 53/2013.

### Flow cytometry analysis and cell sorting

*Ex vivo* and cultured PBMC were stained with APC‐H7 (Tonbo Biosciences, San Diego, CA) or Brilliant Violet 405 (Molecular Probes, Eugene, OR) viability dye in the presence of different panels of monoclonal antibodies directed to human CD3, CD19, CD20, CD56, CD14, CD16, CD40, CD86, HLA‐DR, CD11c, CD1c, CD141, CD64, and PD‐L1 (Biolegend, San Diego, CA). Human cDC2 and cDC1 were identified as Lineage (CD3, CD14, CD19) negative, CD11c^+^ HLA‐DR^+^ CD1c^+^, or CD141^+^ cells, respectively. Mo subsets were identified as CD14^hi^ CD16^−^ (C); CD14^+^ CD16^+^ (T), and CD14^lo^ CD16^hi^ (NC) cells. Additional panels using anti‐human CD56, CD16, PCNA, MICa/b, ULBP1, NKG2D, NKp30, CD107a, TNFα, IFNγ (BioLegend, San Diego, CA), and NKG2A (RyD systems, Minneapolis, MN) were used for validation purposes (see Appendix Table S3). In these panels, granulocytes were excluded based on FSC‐A vs. SSC‐A and singlet gating strategies. To address intrinsic spontaneous secretion of IFNγ, TNFα, and degranulation measured as accumulation of CD107a in the membrane in NK cells from the study cohorts, we directly cultured cells *ex vivo* in RPMI 10% FBS during 4 h in the absence of any stimuli and in the presence of 5 μg/ml Brefeldin A (Sigma‐Aldrich), Monesin (Sigma‐Aldrich), and an anti‐CD107a antibody (BioLegend). For *in vivo* experiments, anti‐mouse CD3, CD11b, NK1.1 (Tonbo Biosciences), RAE‐1, CD27, XCR1, CD107a (BioLegend), CD45.2, IFNγ, CD11b (eBioscience), CD64, CD11c, MCH‐II, Ly6C, CD3, CD45.2 (BD Bioscience), and NKG2D (ThermoFisher) (see Appendix Table S5). Samples were analyzed on a Fortessa cytometer (BD Biosciences, San Jose, CA) at Centro Nacional de Investigaciones Cardiovasculares (CNIC, Madrid, Spain). Analysis of individual and multiparametric flow cytometry data was performed using FlowJo software (Tree Star). Human cDC2 and cDC1, Mo, and CD56^+^ NK cells were sorted from PBMCs, using a FACS Aria II sorter (BD Biosciences), from pSS patients (untreated) and HD following the next gating strategy: viable human Lin^−^ (CD3, CD19, CD20, CD56), CD14^−^ CD11c^+^ HLADR^+^ CD1c^+^ cDCs (cDC2); CD14^−^ CD11c^+^ HLADR^+^ CD141^+^ cDCs (cDC1); total Lin^−^ CD14^+^. Samples from *n* = 4 pSS patients and *n* = 4 HD were used for transcriptional analyses and *n* = 8 pSS patients and *n* = 6 HD for *ex vivo* functional analyses. For functional analysis of NK cells, a modified panel (Lineage cocktail containing only anti‐CD3, CD19, CD20 mAbs) was used allowing also the separation of Lin^−^ HLADR^−^ CD56^+^.

For *in vivo* experiments, cells from PB and SMSG were used for phenotypical characterization of myeloid and NK cell subsets by flow cytometry, while others were cultured in the presence of phorbol 12‐myristate 13‐acetate (50 ng/ml) and ionomycin (1 μg/ml; Sigma‐Aldrich, San Luis, MO) and incubated at 1 h at 37°C in 5% CO_2_ and then another 4 h in the presence of 5 μg/ml Brefeldin A (Sigma‐Aldrich), Monesin (Sigma‐Aldrich), and an anti‐CD107a antibody (BioLegend). In all cases, cells were firstly incubated with anti‐FcRII/III and LIVE/DEAD Fixable Yellow Dead Cell Stain (Invitrogen, Waltham, MA) and then stained with specific antibodies (Appendix Table S3). The Fixation/Permeabilization Solution Kit (BD Biosciences, San Jose, CA) was used for intracellular staining. Trucount Absolute Counting Tubes (BD Biosciences) were added to obtain and homogenize absolute cell counts. Samples were acquired in an LSRFortessa and FACSCanto Flow Cytometer (BD Biosciences) and analyzed using the FlowJo software (Tree Star). Results were corroborated in two independent experiments.

Analysis of proportions of hematopoietic CD45.2^+^ cells or absolute numbers in the SG of murine Ly6C^+^ Monocytes, cDC2 (Ly6C^−^ MCH‐II^+^ CD11c^Hi^ CD11b^+^ XCR1^−^), cDC1 (Ly6C^−^ MCH‐II^+^ CD11c^Hi^ CD11b^−^ XCR1^+^) subsets, and CD3^−^ NK1.1^+^ NK subsets defined by CD11b vs. CD27 expression within the infiltrate were longitudinally analyzed at 8 and 14 days after treatment initiation by sacrificing *n* = 5 mice per group at each timepoint. These DC subpopulations were defined within CD11c^hi^ cells. Levels of activation/maturation markers such as CD64 were analyzed to define inflammatory cDC2 and activation levels of other gated myeloid population. In addition, levels of NKG2D were determined on NK cell subsets.

### Longitudinal analysis of pathology parameters, innate and adaptive immune cell subsets in an *in vivo* model of poly I:C stimulation

We used an *in vivo* model based on the intraperitoneal injection of poly I:C of mice which has been reported to induce infiltration of immune cells into the SG (Nandula *et al*, 2013; Maria *et al*, 2017). Two groups of *n* = 19 female C57BL/6 wild‐type mice aged 6–8 week were intraperitoneally (i.p.) injected either with 50 μg of poly I:C (Invivogen, San Diego, CA) or PBS in the same quantity every 2 for 14 days and analyzed at 1 week post injection in four independent experiments. Mice receiving the two treatments were randomized together in different cages. Blinding of experimental conditions was performed for flow cytometry analysis. Animals that died before the time point of analysis were excluded from the study. Dynamics of cDC2 and NK cells were analyzed at different time points. In some experiments, analyses were extended to 2, 3, and 8 weeks post injection for further phenotypical analysis of innate and adaptive immune cells in PB and SMSG. Right SGs were extracted and treated for 20 min at 37°C with 250 μg/ml Liberase TL and 100 μg/ml DNAase I (Roche, Basel, Switzerland) in HBSS medium to facilitate tissue enzymatic digestion and cell disaggregation. Single‐cell suspensions were then obtained by grating the digested organs through a 70 μm cell strainer (Falcon). Using this model, we analyzed the proportions of infiltrated hematopoietic cells and the enrichment on NK cell and cDC1 and cDC2 subsets in the submandibular SG (SMSG) of mice injected with poly I:C compared to those receiving PBS at two early timepoints (1 and 2 weeks) after treatment initiation before the disease onset and at 3 weeks after interruption of poly I:C injection, when adaptive immune cells have been described to be present in the SMSG in this model.

Left SMSGs were preserved in paraffin for subsequent immunohistochemistry staining containing hematoxilin and Rabbit anti‐mouse Pax5 primary and antirabbit‐HRP secondary antibodies to define aggregates of inflammatory infiltrated immune cells, and these areas were quantified with QuPath v0.4.3 software after taking images of total tissue with the Zeiss microscopy. Moreover, two groups of mice either receiving PBS or poly I:C were used to test salivary gland hypofunction as previously described (Deshmukh *et al*, 2009). Pilocarpine‐induced saliva was collected at 1, 2, 3, and 8 weeks after PBS or poly I:C treatment. Mice were anesthetized with Ketamine/Xylazine and injected with a freshly prepared solution of pilocarpine hydrochloride (Sigma‐Alrich) by intraperitoneal route (0.5 μg/g body weight) and let it act for a 2 min interval. Subsequently, saliva was collected directly from the mouth of mice during 20 min in a cotton piece, which was transferred into an Eppendorf tube and finally weighted. Saliva weight was calculated after subtracting the weight of control empty tubes.

### Gene expression analysis by RNA‐Seq and computational data analysis

Total RNA was isolated from sorted primary PB Mo, cDC2, and cDC1 subsets from *n* = 4 untreated pSS patients and *n* = 4 HD, using the RNeasy Micro Kit (Qiagen). Quality and integrity of each RNA sample was checked using a Bioanalyzer prior to proceeding to RNAseq protocol. Subsequently, selected RNAs from cDCs were used to amplify the cDNA using the SMART‐Seq v4 Ultra Low Input RNA Kit (Clontech‐Takara). Amplified (1 ng) cDNA was used to generate barcoded libraries using the Nextera XT DNA library preparation kit (Illumina, San Diego, CA). The size of the libraries was checked using the Agilent 2100 Bioanalyzer High Sensitivity DNA chip and their concentration was determined using the Qubit® fluorometer (ThermoFisher Scientific, Waltham, MA).

RNA from circulating Mo was processed as follows: 200 ng of total RNA were used to generate barcoded RNA‐seq libraries using the NEBNext Ultra II Directional RNA Library Prep Kit (New England Biolabs Inc). Libraries were sequenced on a HiSeq 2500 (Illumina) and processed with RTA v1.18.66.3. FastQ files for each sample were obtained using bcl2fastq v2.20.0.422 software (Illumina).

Sequencing reads were aligned to the human reference transcriptome (GRCh38 v91) and quantified with RSem v1.3.1 (Li & Dewey, 2011). Raw counts were normalized with TPM (Transcripts per Million) and TMM (Trimmed Mean of M‐values) methods, transformed into log2 expression (log2(rawCount+1)), and compared to calculate fold‐change and corrected *P*‐value. Only those genes expressed with at least 1 count in a number of samples equal to the number of replicate samples of the condition with less replicates were taken into account. Gene expression changes were considered significant if associated to Benjamini and Hochberg adjusted *P*‐value < 0.05.

Heatmaps were generated with Morpheus online tool from Broad Institute (https://software.broadinstitute.org/morpheus). Pathway analysis and visualization of gene networks for each DEG list was performed using Ingenuity Pathway Analysis (Qiagen) and NetworkAnalyst (Zhou *et al*, 2019) Softwares. Finally, the Chordplot connecting selected IPA canonical pathways and the containing DEG was generated using Goplot R package (Walter *et al*, 2015).

### Validation of gene expression by RT–qPCR

RNA was isolated from sorted PB myeloid subsets using RNeasy Micro Kit (Qiagen) according to manufacturer's instructions. cDNA was synthesized using the reverse transcription kit (Promega) and transcriptional expression of IFIT1 (Fw: 5′‐GGAATACACAACCTACTAGCC‐3′; Rv: 5′‐CCAGGTCACCAGACTCCTCA‐3′), (Rv: 5′‐AGGAGAATTCTGGGTTGTTGGGCT‐3′), DDX60 (Fw 5′‐AAGGTGTTCCTTGATGATCTCC‐3′ Rv: 5′‐TGACAATGGGAGTTGATATTCC‐3′), STAT1 (Fw: 5′‐ATGGCAGTCTGGCGGCTGAATTSTAT1‐3′; Rv: 5′‐CCAAACCAGGCTGGCACAATTG‐3′), IL‐15 (Fw: 5′‐GGATTTACCGTGGCTTTGAGTAATGAG‐3′; Rv: 5′‐GAATCAATTGCAATCAAGAAGTG‐3′) was analyzed by semiquantitative PCR using the SYBR Green assay GoTaq® qPCR Master Mix (Promega) with standardized primers (Metabion). TaqMan Gene expression Assays were also used to determine transcriptional expression of RIG‐I (Hs01061436_m1) and β‐Actin (Hs01060665_g1). qPCR amplifications were performed on a StepOne Real‐Time PCR system (Applied Biosystems). Relative gene expression was calculated using technical replicates and normalized to β‐Actin detection.

### siRNA‐mediated gene knockdown

Gene knockdown of DDX60, DDX58 (RIG‐I), IFIT1, and STAT1 was performed by nucleofection of primary cDCs isolated from HD with specific siRNAs (SMART‐pool, Horizon Discovery) or irrelevant scramble siRNA in an Amaxa4D‐Nucleofector (Lonza) instrument using the CM120 protocol and following the manufacturer's instructions. siRNA‐mediated knockdown was confirmed after 24 h by RT–qPCR analysis of mRNA levels for target genes.

### Immunomagnetic isolation of primary cDC2 and NK cells

Total cDC2 were purified from total PBMC suspensions by negative immunomagnetic methods (purity > 90%) using the Human Myeloid DC Enrichment Kit (STEMCELL). Untouched NK cells were also isolated by immunomagnetic selection from autologous PBMC samples using a negative selection kit (purity > 90%) “EasySep™ Human NK Cell Isolation Kit (STEM CELL).”

### Killing assays

Natural cytotoxic function of circulating NK cells from HD and pSS patients, selected based on characteristic differential levels of CD56^hi^ CD16^+^ NK cell enrichment in these individuals (median 7.059) as compared to control donors (median 1.47), were tested *in vitro* by coculture with the target Green Fluorescent Protein (GFP) expressing K562 cell line (NIH Reagent Program 116799). Briefly, NK cells were isolated by immunomagnetic selection as described above and labeled with Violet Cell Proliferation Tracker according to manufacturer's instructions (C34557, ThermoFisher) and subsequently cultured at a NK: GFP‐K562 target ratio 5:1 for 16 h. GFP‐K562 targets were also cultured individually in the absence of NK cells as a control. Specific Killing was determined by flow cytometry by excluding Violet Cell Tracker^+^ NK cells and determining the proportions of cells losing GFP expression and acquiring Live‐Dead Viability Staining (see Appendix Fig S2B).

### *In vitro* stimulation of DCs and functional assays

cDC2 from PBMC of HD or pSS patients were cultured for 24 h in the presence of media alone or 1.5 μg/ml poly I:C (SIGMA) according to manufacturer's instructions. After 24 h, maturation of cDC was evaluated by FACS analysis of expression of maturation markers (CD40, CD86) and ligand for activating (MICa/b) receptor in NK cells.

For functional assays, *ex vivo* cDCs from HD and pSS individuals were co‐cultured directly with autologous CD56^+^ NK cells for 24 h at a 1:2 ratio in media supplemented with 50 IU/ml recombinant human IL‐2 (Prepotech). A negative control of NK cell activation cultured in media only was also included. After 24 h of culture, expression of CD16, CD56, and CD107a was analyzed on gated NK cells by flow cytometry. In some of these experiments, a blocking anti‐MICa/b (4 μg/ml/159207/R&D Systems) or anti‐IL‐15 (4 μg/ml/MAB2471/R&D Systems) or their corresponding isotypic controls (4 μg/ml/402202 and 401402/BioLegend) Abs were added to the cultures to address the functional relevance of NKG2D‐ligand and IL‐15‐receptor interactions. The same conditions were used in functional assays performed with cDCs nucleofected with siRNAs and exposed to media or poly I:C, as previously described. In some experiments, media was supplemented with 1.5 μg/ml Brefeldin A (SIGMA) and Monensin (SIGMA) and anti‐human CD107a mAbs for subsequent analysis of degranulation and intracellular expression of IFNγ and TNFα by flow cytometry at the end of the assay. To address the regulation of NKG2D ligands by IFN I receptor, we isolated cDC2 from the spleen of 8–12 weeks‐old female of C57BL/6 (WT) and B6N.129P2‐Tyk2KO tm1Biat (Tyk2^−/−^) mice (on a C57BL/6 background) (27) (kindly provided by Dr. M. Müller, Institute of Animal Breeding and Genetics, Vienna, Austria) bred and maintained in the animal facilities at the School of Medicine from the Universidad Autónoma de Madrid. All experiments with these mice were performed in accordance with the approval of the National RD 53/2013 and European Union 2010/63/EU directive and under the EU and National Animal Care guidelines. All protocols were approved by Consejería de Medio Ambiente de la Comunidad de Madrid (PROEX 353.8/21). Briefly, splenocytes were treated with a cocktail of NK1.1, CD8a, XCR1, Ly6C, Ly6G, and B220 Abs labeled with PE and these linage cells (NK, CD8 T cells, B cells, Mo, granulocytes, cDC1) were depleted using anti‐PE beads. Subsequently, cDC2 were positively selected using CD11c micropure beads through AutoMasc system (Miltneyi Biotec). Finally, levels of RAE‐I were determined on isolated cDC2 cultured for 16 h in the presence of media or poly I:C.

### Statistics

The statistical significance of differences between the cells from different or within the same patient cohorts (pSS or HD) and in the longitudinal *in vivo* studies were assessed using Mann Whitney *U* or Wilcoxon matched‐pairs signed‐rank tests. Non‐parametric Spearman correlation was performed to test association of mentioned parameters. A *P* value less than 0.05 was considered significant. Statistical analyses were performed using GraphPad Prism 9.0 software.

### Histological analysis of pSS salivary glands by immunofluorescence

Salivary gland biopsies from *n* = 7 pSS patients were embedded in paraffin and segmented in 3 μm fragments. Tissue sections deparaffinization, hydration, and target retrieval were performed with a PT‐LINK (Dako) before immune‐staining. For immuno‐staining of paraffin‐preserved tissue, we used goat and mouse anti‐human CD56 (RyD Systems; ThermoFisher), mouse anti‐human CD1c cDC (Abcam), goat anti‐human MICa/b (RyD Systems), rat anti‐human Granzyme B (eBioscience), rabbit anti‐human HLA‐DR (Abcam), rat anti‐human CD19 (ThermoFisher), goat anti‐human IL‐17a (RyD Systems) as primary antibodies; and donkey anti‐rabbit AF488 (Invitrogen), donkey anti‐goat AF488 (Invitrogen), donkey anti‐rat AF594 (Jackson ImmunoResearch), donkey anti‐goat AF568, donkey anti‐goat AF647 (Invitrogen), and donkey anti‐mouse AF647 (Thermo Fisher) as secondary antibodies (see Appendix Table S5). Images were taken with a Leica TCS SP5 confocal and processed with the LAS AF software. Detection of high CD1c^+^ HLADR^+^ cells with dendritic morphology defining cDC2, detection of MICa/b^+^ cells, CD56^+^ NK cells, and their co‐localization with Granzyme B were analyzed with ImageJ software. In some cases, SG tissue sections were also stained with hematoxylin and eosin to discriminate extracellular matrix defining areas with different levels of infiltrates.

## Author contributions

**Ildefonso Sánchez‐Cerrillo:** Conceptualization; data curation; software; formal analysis; investigation; methodology; writing – original draft; writing – review and editing. **Diego Calzada‐Fraile:** Validation; methodology; writing – review and editing. **Ana Triguero‐Martínez:** Conceptualization; validation; writing – review and editing. **Marta Calvet‐Mirabent:** Conceptualization; formal analysis. **Olga Popova:** Data curation; investigation. **Cristina Delgado‐Arévalo:** Investigation; methodology. **Mariel Valdivia‐Mazeyra:** Resources; investigation. **Marta Ramírez‐Huesca:** Investigation; methodology. **Enrique Vázquez de Luis:** Formal analysis; validation. **Alberto Benguría:** Formal analysis; methodology. **Teresa Aceña‐Gonzalo:** Validation; investigation. **Roberto Moreno‐Vellisca:** Formal analysis; methodology. **Magdalena Adrados de Llano:** Formal analysis; validation; methodology. **Hortensia de la Fuente:** Investigation; methodology; project administration. **Ilya Tsukalov:** Formal analysis; investigation. **Pablo Delgado‐Wicke:** Investigation. **Elena Fernández‐Ruiz:** Resources; funding acquisition; validation. **Emilia Roy‐Vallejo:** Data curation. **Reyes Tejedor‐Lázaro:** Data curation; formal analysis. **Almudena Ramiro:** Validation; visualization; writing – review and editing. **Salvador Iborra:** Conceptualization; data curation; visualization; writing – review and editing. **Francisco Sánchez‐Madrid:** Resources; supervision; funding acquisition; validation; visualization; project administration; writing – review and editing. **Ana Dopazo:** Validation; visualization; methodology. **Isidoro González Álvaro:** Conceptualization; software; funding acquisition; validation; visualization; project administration; writing – review and editing. **Santos Castañeda:** Conceptualization; supervision; funding acquisition; validation; visualization; project administration; writing – review and editing. **Enrique Martin‐Gayo:** Conceptualization; resources; data curation; supervision; funding acquisition; validation; investigation; visualization; methodology; writing – original draft; project administration; writing – review and editing.

## Disclosure and competing interests statement

IGA reports the following competing interests: grants from Instituto de Salud Carlos III, during the course of the study; personal fees from Lilly and Sanofi; personal fees and non‐financial support from BMS; personal fees and non‐financial support from Abbvie; research support, personal fees, and non‐financial support from Roche Laboratories; non‐financial support from MSD, Pfizer, and Novartis, not related to the submitted work. The rest of authors declare that they have no conflict of interest.

## Supporting information



Appendix

Source Data for Figure 1

Source Data for Figure 2

Source Data for Figure 3

Source Data for Figure 4

Source Data for Figure 5

## Data Availability

RNA‐seq data from the study has been deposited and are accessible on the public Gene Expression Omnibus (GEO) repository, accession number: GSE157047 (https://www.ncbi.nlm.nih.gov/geo/query/acc.cgi?acc=GSE157047) and GSE194263 (https://www.ncbi.nlm.nih.gov/geo/query/acc.cgi?acc=GSE194263).
