# Supplementary material for: MICa/b‐dependent activation of natural killer cells by CD64 + inflammatory type 2 dendritic cells contributes to autoimmunity
Source: EMBO J. 2023 Nov 2;42(23):e113714. doi: 10.15252/embj.2023113714 (PMC10690448; doi:10.15252/embj.2023113714)
Supplement: Supplementary file 1 — Appendix [file EMBJ-42-e113714-s001.pdf]

Table of contents:

- Appendix Figure S1 and legend…………………………Page 2-3
- Appendix Figure S2 and legend…………………………Page 4-5
- Appendix Figure S3 and legend…………………………Page 6-7
- Appendix Figure S4 and legend…………………………Page 8-9
- Appendix Figure S5 and legend…………………………Page 10-11
- Appendix Figure S6 and legend…………………………Page 12-13
- Appendix Figure S7 and legend…………………………Page 14-15
- Appendix Figure S8 and legend…………………………Page 16-17
- Appendix Figure S9 and legend…………………………Page 18-19
- Appendix Figure S10 and legend………………………..Page 20-21
- Appendix Table S1 and legend……………………………Page 22-23
- Appendix Table S2 and legend……………………………Page 24
- Appendix Table S3 and legend……………………………Page 25
- Appendix Table S4 and legend……………………………Page 26-96
- Appendix Table S5 and legend……………………………Page 97-100

Appendix Figure S1

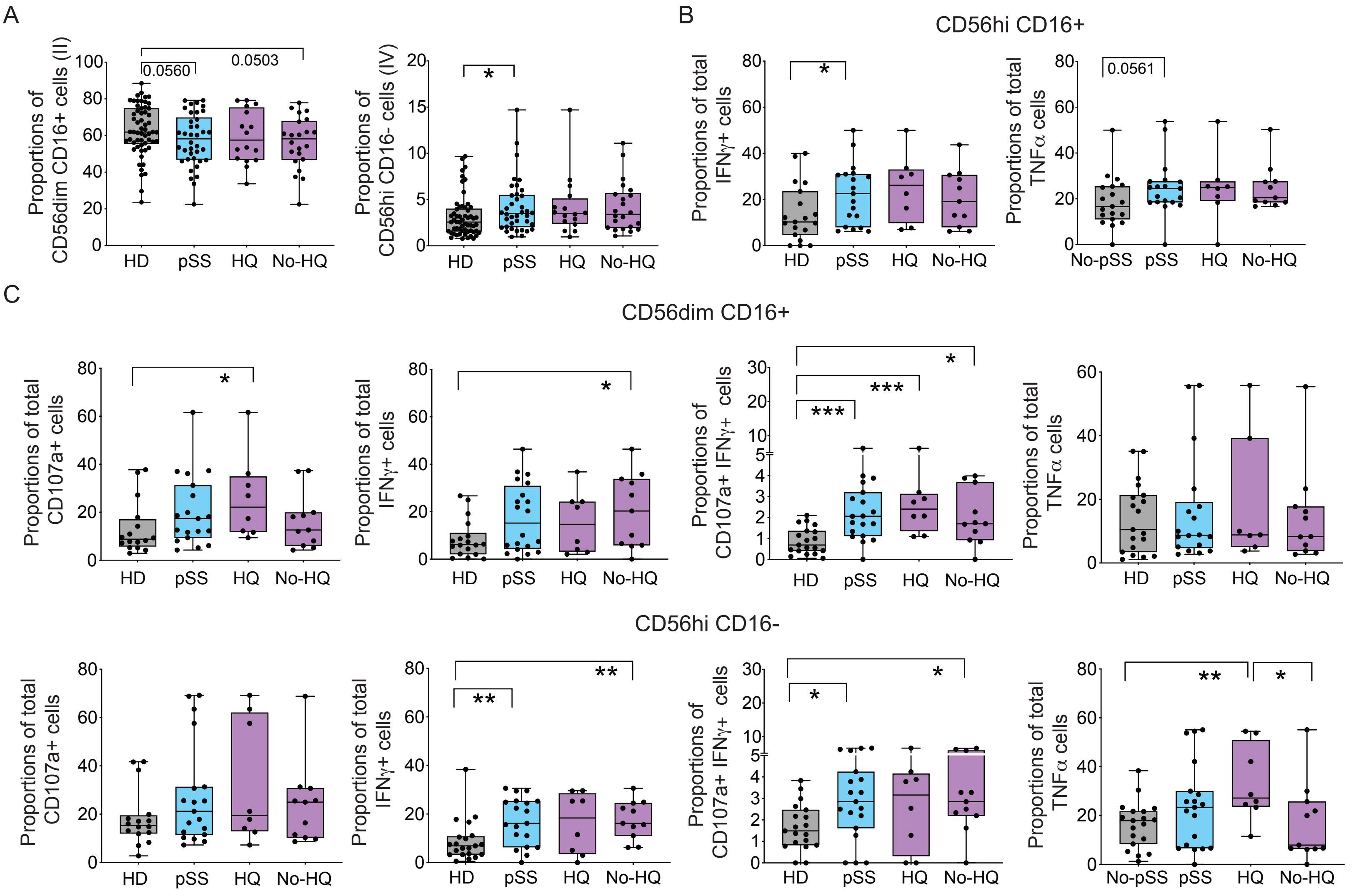

Appendix Figure S1. Phenotypic analysis of natural killer cell populations from pSS patients.

(A): Summarized proportions of circulating NK cell subsets defined in figure 1 including $CD56^{dim}$ $CD16^{+}$ (II) and $CD56^{hi}$ $CD16^{-}$ (IV) NK subsets in n= 56 healthy donors (HD; grey) and n= 42 total or n=17 HQ and n=25 No-HQ primary Sjögren´s syndrome (pSS; blue) patients.

(B-C): Proportions of cells expressing CD107a (B, C), and frequencies of total $IFN\gamma^{+}$, total $TNF\alpha^{+}$, $IFN\gamma^{+}$ $CD107a^{+}$ (C) within the indicated circulating NK cell subsets from 16 HD (grey) and n= 19 total or HQ stratified (n=8 HQ, n=11 No-HQ) pSS patients (blue).

Data information: Data is represented as box and whiskers plots with maximum and minimum range and a median value central band. Data points are representing biological replicates. Statistical significance was calculated using a two-tailed Mann Whitney test. *$p<0.05$; **$p<0.01$; ***$p<0.001$.

Appendix Figure S2

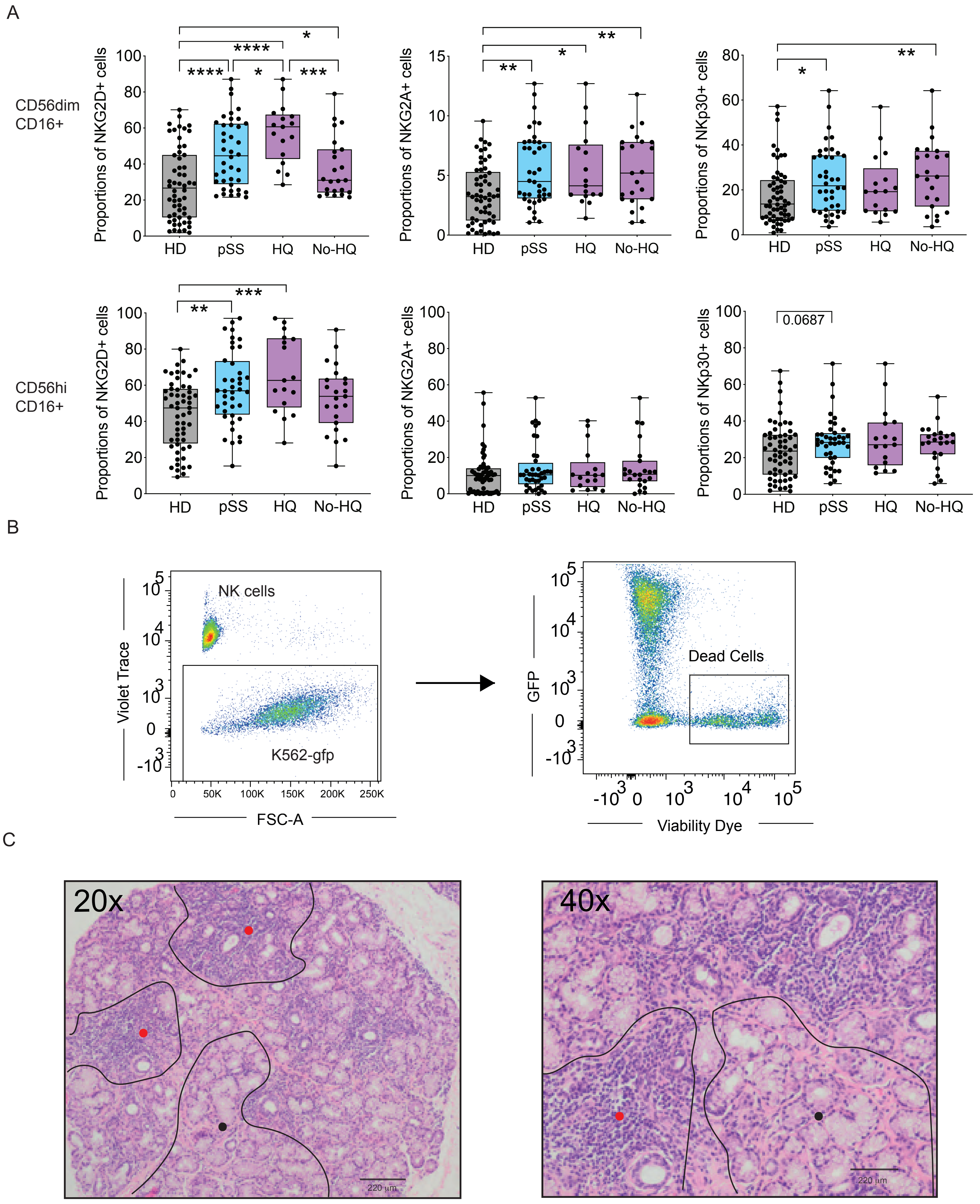

Appendix Figure S2. Characterization of activating and inhibitory receptors and functional analysis of NK cells from pSS patients.

(A): Proportions of NKG2D (left plot), NKG2A (middle plot) and NKp30 (right plot) receptors on de indicated NK cell subsets from primary Sjögren´s syndrome (total n=40 pSS; blue; HQ n=17; No-HQ n=23 purple) and healthy donors (n= 56 HD; grey).

(B): Flow cytometry gating strategy representing frequencies of dead K562-GFP after culture in medium alone or in the presence of isolated NK cells. K562 cells were identified as Violet Trace negative cells to exclude NK cells. Dead K562 were quantified as cells losing GFP expression and acquiring of staining of the viability dye marker.

(C): Eosin/hemotoxilin tissue sections from SG tissue of a representative patient to different magnifications 20x (left) and 40x (right), showing low (black stars) and highly (red stars) infiltrated areas with altered morphology.

Data information (A): Data is represented as box and whiskers plots with maximum and minimum range and a median value central band. Data points represented biological replicates. Statistical significance was calculated using a two-tailed Mann Whitney test. *$p<0.05$; **$p<0.01$; ***$p<0.001$; ****$p<0.0001$.

Appendix Figure S3

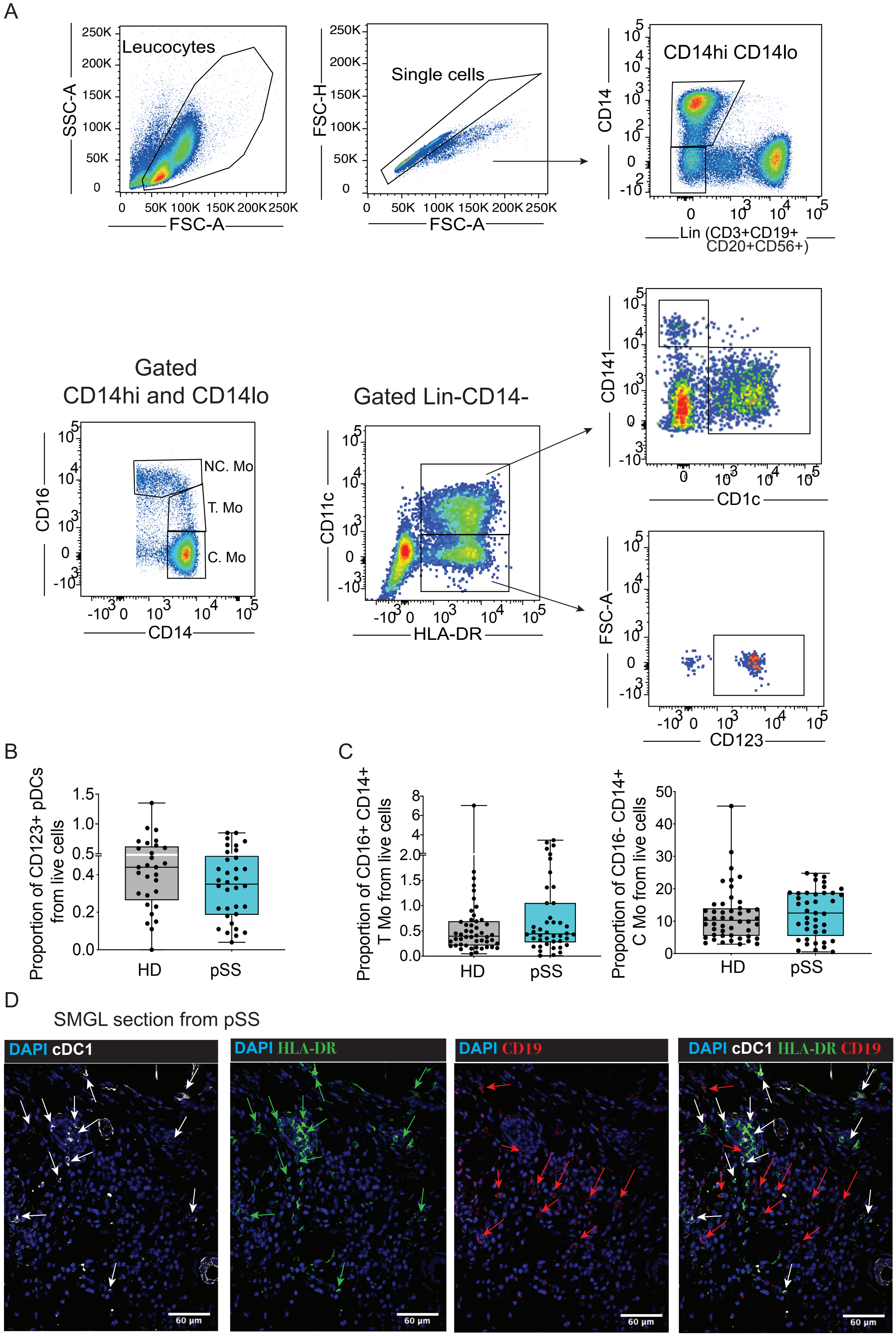

Appendix Figure S3. Identification and frequencies of circulating myeloid subsets from pSS patients.

(A): Flow cytometry gating strategy identifying $CD14^{+}$ Monocytes and HLA-DR^{+} $CD11c^{+}$ conventional (cDC) subdivided into $CD1c^{+}$ cDC2 and $CD141^{+}$ cDC1 and $CD11c^{-}$ $CD123^{+}$ plasmacytoid (pDC) dendritic cells from Lineage (CD3, CD19, CD20, CD56) negative $CD14^{-}$ cells. $CD14^{+}$ Mo was further defined as $CD14^{lo}$ $CD16^{hi}$ non classical (NC), $CD14^{+}$ $CD16^{+}$ transitional (T) and $CD14^{+}$ $CD16^{-}$ classical (C) Mo on the basis of the expression of CD16.

(B-C): Box and whisker plots representing frequencies of Lin^{-} HLA-DR^{+} $CD11c^{-}$ $CD123^{+}$ pDCs (B), T Mo and C Mo subsets (C) in live mononuclear cells from PB from HD (grey, n=48) and total pSS patients (blue, n=44) obtained by flow cytometry.

(D): Representative confocal microscopy (40x magnification) showing immunofluorescence analysis of CD1c (white), HLADR (green) and CD19 (red) expression on a representative SG tissue section from a pSS patient. Cell nuclei were stained with DAPI (blue). cDC2 co-expressing CD1c and high levels of HLADR with dendritic cell morphology are highlighted with white arrows. $CD19^{+}$ cells expressing low levels of CD1c and HLA-DR are highlighted with red arrows. A 60μm Scale bar is included in each image.

Data information (B): Data is represented as box and whiskers plots with maximum and minimum range and a median value central band. Data points represented biological replicates.

Appendix Figure S4

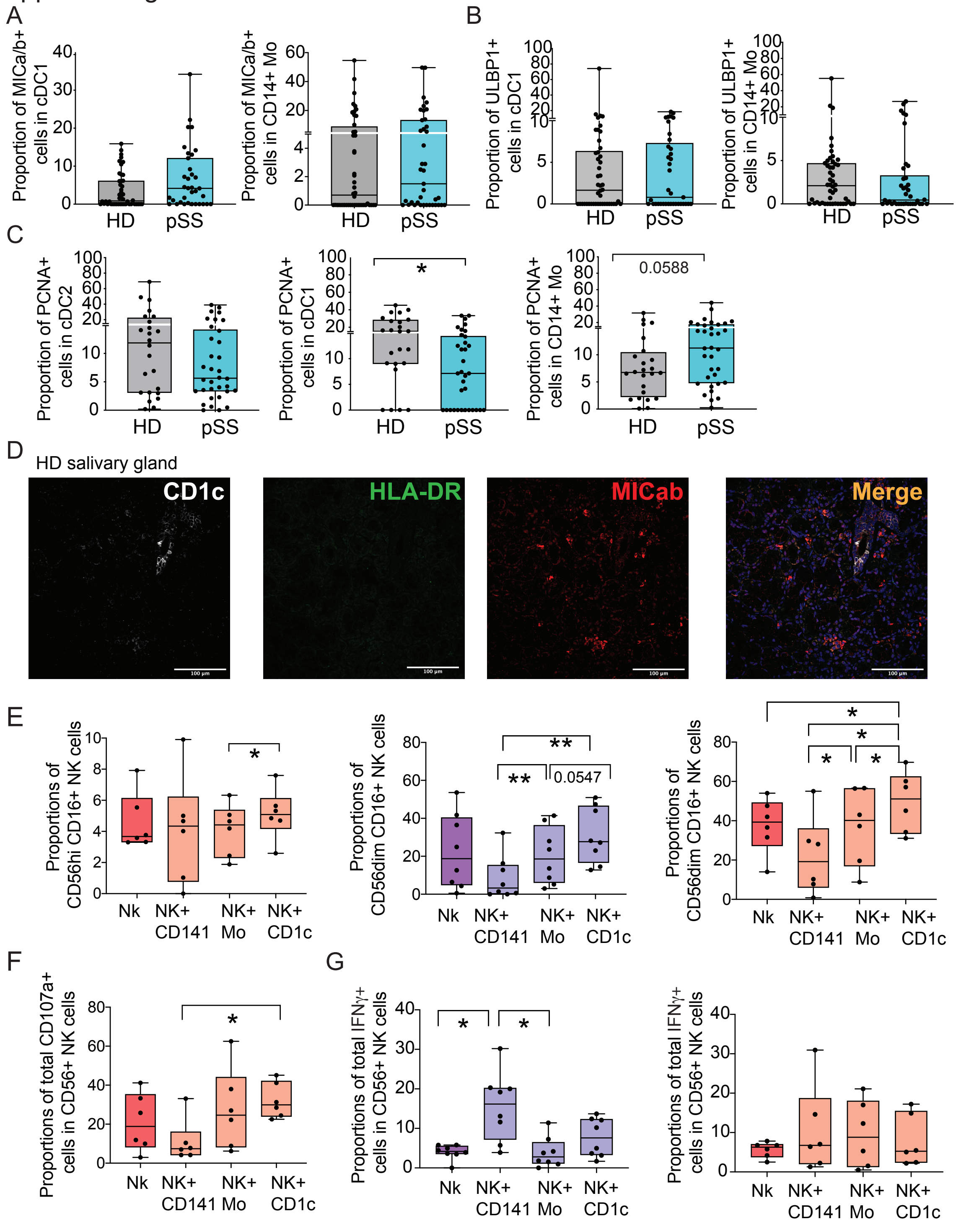

Appendix Figure S4. Functional ability of cDC2, cDC1 and Mo to stimulate NK cells

(A, B,C): Proportions of MICa/b^{+} (A), ULBP1^{+} (B) PCNA^{+} (C) cells from circulating cDC1 and Mo in healthy donors (HD; grey n=46 A,B; n=24 C) and primary Sjögren´s syndrome (pSS; blue; n=42 A,B; n=34 C) individuals. Levels of PCNA on cDC2 are also included.

(D): Representative confocal microscopy (40x magnification) showing immunofluorescence analysis of CD1c (white), HLADR (green) and MICab (red) expression on a representative control SG tissue section from a non-autoimmune mechanical sialadenitis patient from a total of n=3 individuals tested. Cell nuclei were stained with DAPI (blue).

(E): Proportions of the CD56^{hi} CD16^{+} (left) and CD56^{dim} CD16^{+} (middle and right) NK cell subsets from HD (orange) and CD56^{dim} CD16^{+} NK subset from pSS patients (purple), after 16h culture of sorted autologous CD56^{+} NK cell in media alone or in the presence of sorted autologous circulating Mo, cDC2 and cDC1 from n=8 pSS patients and n=6 HD, at ratio 1:2 (myeloid cell:NK).

(F,G): Expression of CD107a from NK cells on HD (F; n=6 biological replicates) and proportions of IFNγ^{+} NK cells from pSS patients and HD (G; n=8 biological replicates) in these functional assays is shown. Statistical significance was calculated using two-tailed Wilcoxon matched pairs signed rank test (*p<0.05; **p<0.01).

Data information (A, B, C, E, F, G): Data is represented as box and whiskers plots with maximum and minimum range and a median value central band. Data points are represented by biological replicates. (C): Statistical significance was calculated using a two-tailed Mann Whitney test. *p<0.05. (E,F,G): Statistical significance was calculated using two-tailed Wilcoxon matched pairs signed rank test (*p<0.05; **p<0.01).

Appendix Figure S5

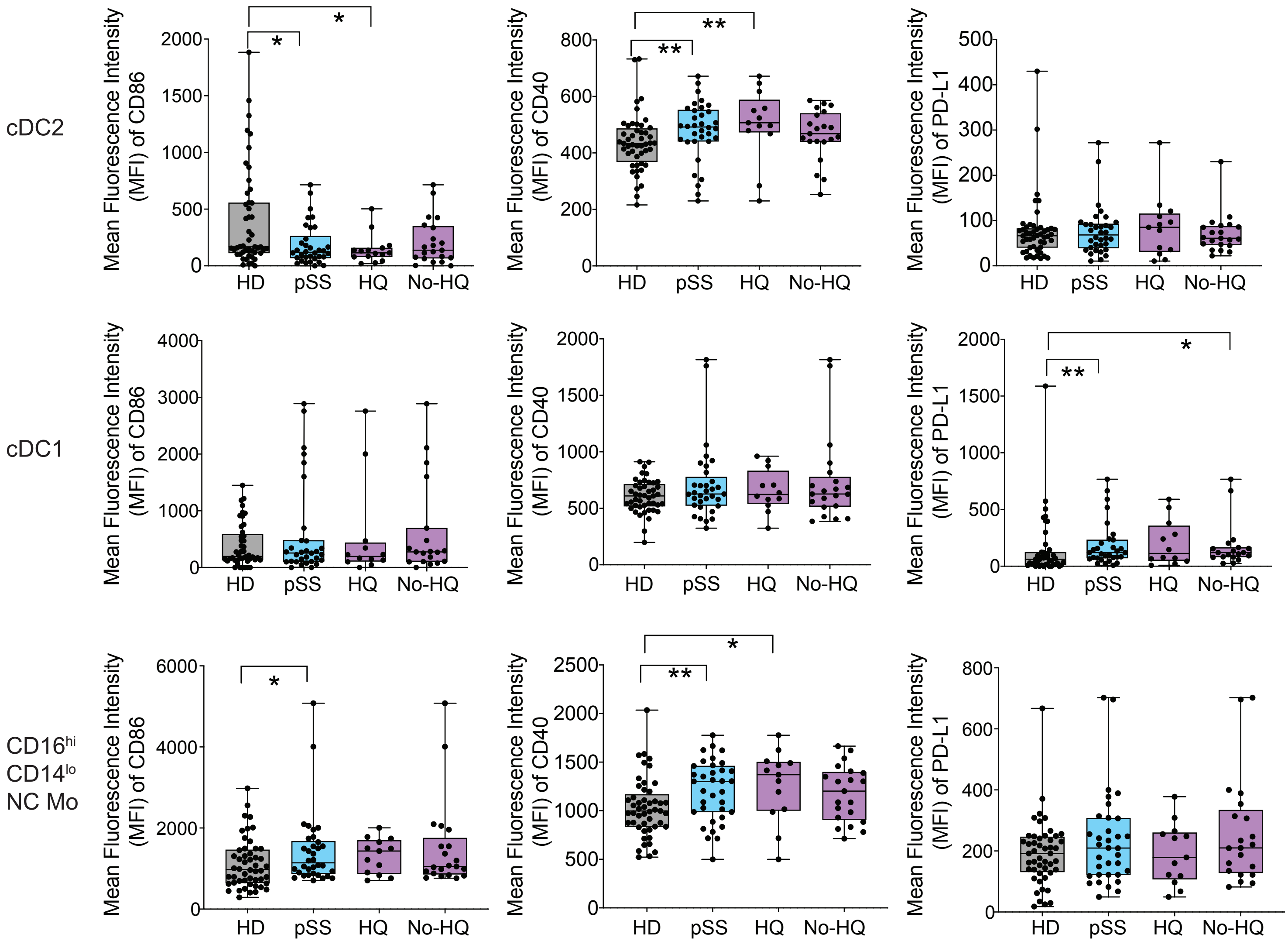

Appendix Figure S5. Phenotypic characteristics of circulating myeloid subsets from pSS patients.

(A-B): Mean Fluorescence Intensity (MFI) of expression of the indicated Interferon stimulated costimulatory molecules on circulating cDC1 and cDC2 and non-classical (NC) Mo from the blood of n=48 healthy donors (HD; grey) and n=34 total (blue) and n=13 HQ and n=21 No-HQ (purple) primary Sjögren´s syndrome (pSS) individuals.

Data information: (A-Data is represented as box and whiskers plots with maximum and minimum range and a median value central band. Data points are represented by biological replicates. Statistical significance was calculated using a two-tailed Mann Whitney test. *$p<0.05$; **$p<0.01$.

Appendix Figure S6

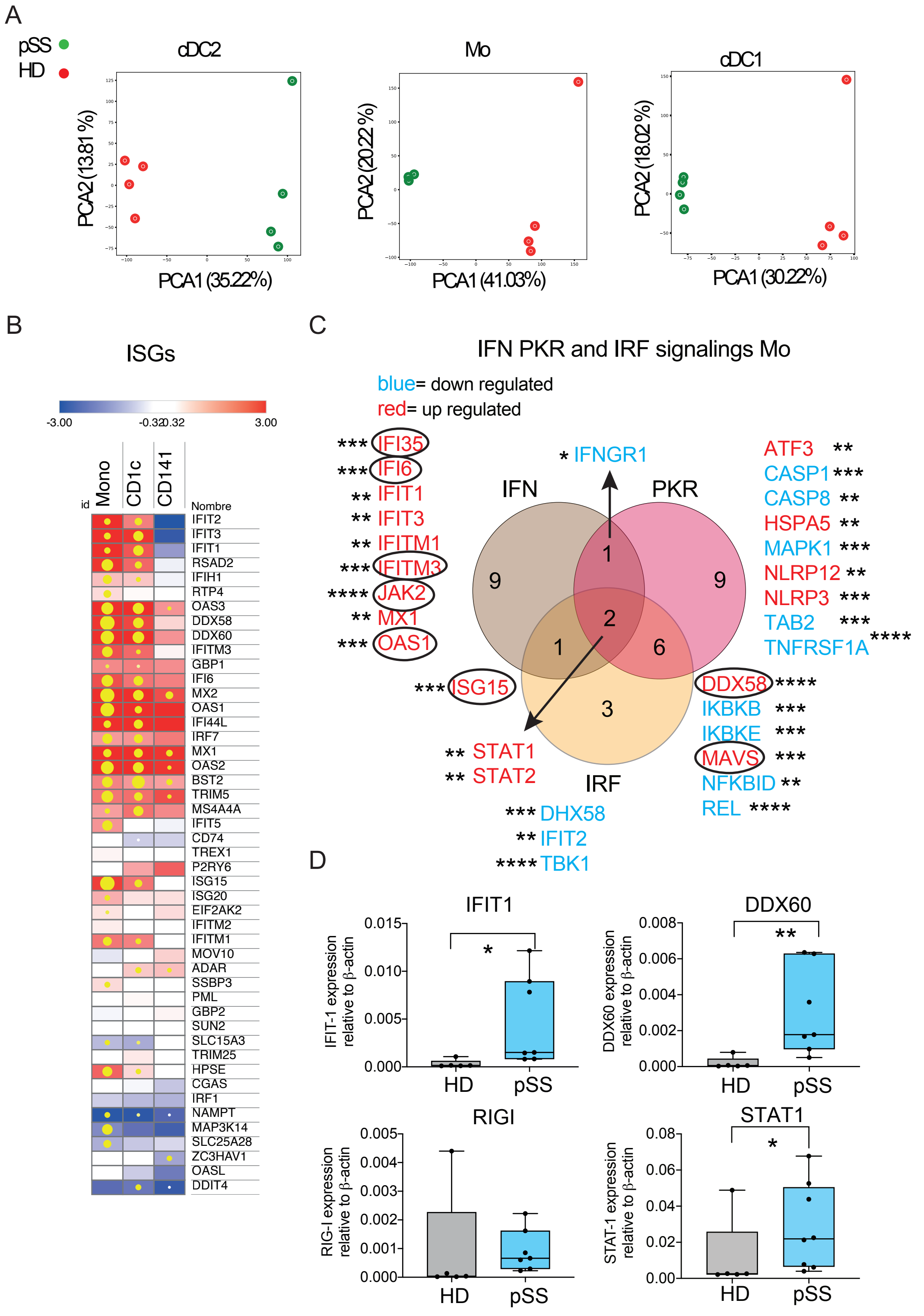

Appendix Figure S6. Pathway analysis of differential transcriptional patterns of Mo and cDC subsets from the blood of pSS patients.

(A): Principal Component Analysis (PCA) representing transcriptional profiles in cDC2, cDC1 and Mo from the blood of n=4 primary Sjögren´s syndrome (pSS; green) and n=4 healthy donors (HD; red).

(B): Heatmap reflecting Log2FC in expression of 47 transcripts associated with IFN signaling or IFN Stimulated Genes (ISG) on Mo, cDC2 and cDC1 from the blood of 4 pSS patients compared to 4 HD. Highlighted Red show upregulated expression and blue, show downregulated pathways. Yellow dots size is proportional to statistical significance of DEG expression (p<0.05 FDR corrected values).

(C): Venn´s diagram of overlapping significant DEG included in selected IFN, PKR and IRF canonical pathways in PB Mo from pSS individuals. Significant levels of differential expression are highlighted for each DEG. *p<0.05; **p<0.01; ***p<0.001; ****p<0.0001.

(D): qPCR validation of the indicated transcript from sorted circulating Mo from n=7 pSS patients (blue) versus n=5 HD (grey) as biological replicates. Statistical significance was calculated using a two tailed U Mann Whitney test. *p<0.05; **p<0.01.

Data information (D): Data is represented as box and whiskers plots with maximum and minimum range and a median value central band.

Appendix Figure S7

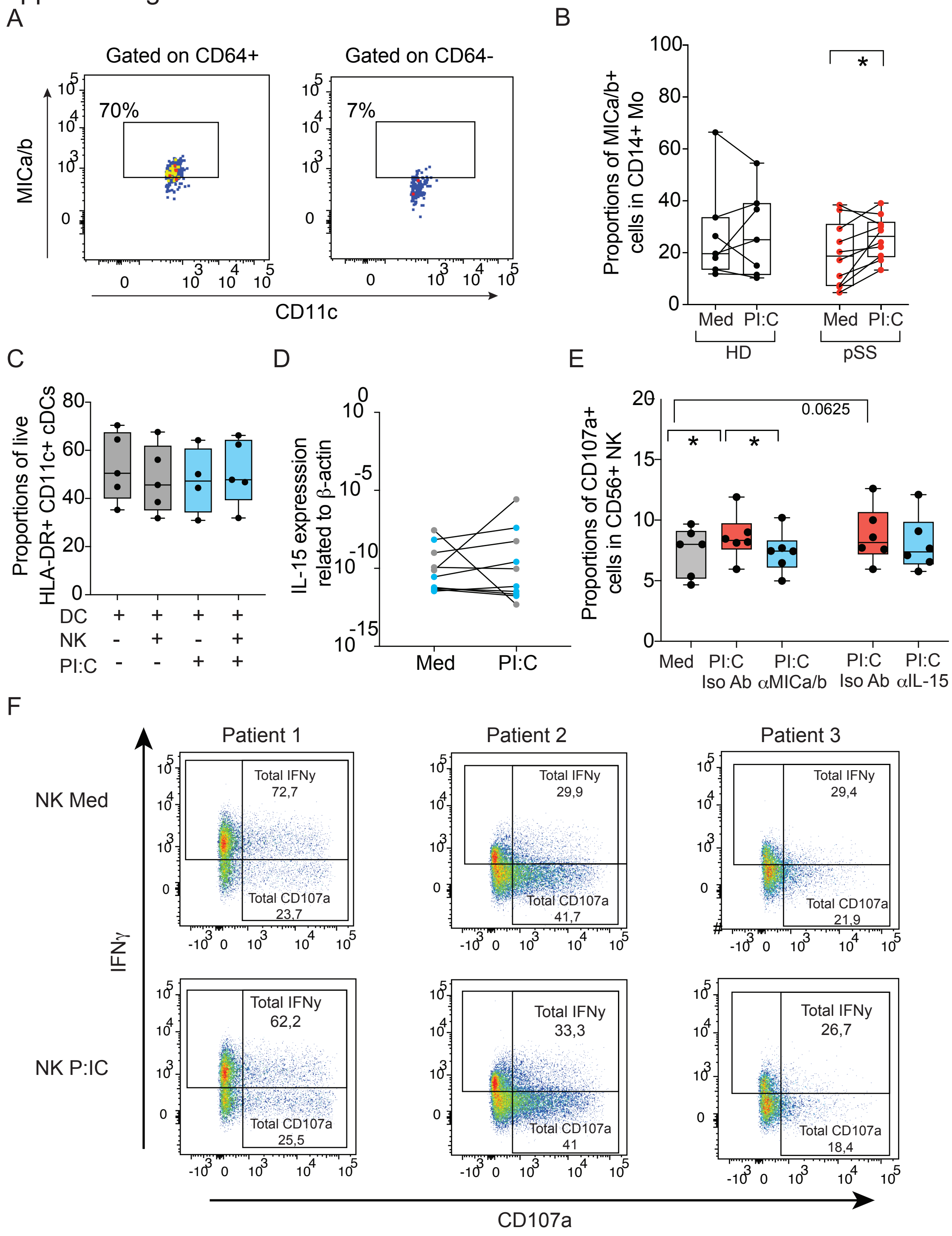

Appendix Figure S7. MICa/b upregulation after poly I:C stimulation in myeloid cells from healthy and pSS individuals Sjögren patients. Impact in NK cell functional activation.

(A): Representative flow cytometry dot plots showing levels of MICa/b versus CD11c expression on gated cDC2 defined by $CD64^+$ and $CD64^-$ expression. Percentages of positive $MICa/b^+$ cells are highlighted on top of each box.

(B): Proportions of cells expressing MICa/b in Mo from the blood of HD (black; n=7 biological replicates) or pSS individuals (red; n=9 biological replicates), after 16h of culture in the presence of poly I:C (PI:C) compared to cells cultured in media only (Med).

(C): Proportions of live $CD11c^+$ $HLADR^+$ cDC2 precultured in media or PI:C and co-cultured in the absence or the presence of autologous NK cells. Mortality was determined by FACS with a viability dye in different biological replicates.

(D): RT-qPCR analysis of IL-15 transcriptional levels relative to beta-actin in cDC2 from HD (grey; n=4) and pSS patients (blue; n=6) as biological replicates, cultured for 16h in Med or PI:C.

(E): Proportions of $CD107a^+$ NK cells in PBMC after culture for 16h in Med or with PI:C and in the absence or the presence of neutralizing antibodies against MICa/b, IL-15 or the corresponding isotypic controls.

(F): Flow cytometry analysis of IFNγ versus CD107a expression on isolated NK cells from three representative healthy donors cultured for 16h in the presence of Med alone or PI:C. Proportions of total $IFN\gamma^+$ and $CD107a^+$ cells are highlighted on the corresponding gates.

Data information (B, C, E): Data is represented as box and whiskers plots with maximum and minimum range and a median value central band. Statistical significance was calculated using two-tailed Wilcoxon matched pairs signed rank test (*$p<0.05$).

Appendix Figure S8

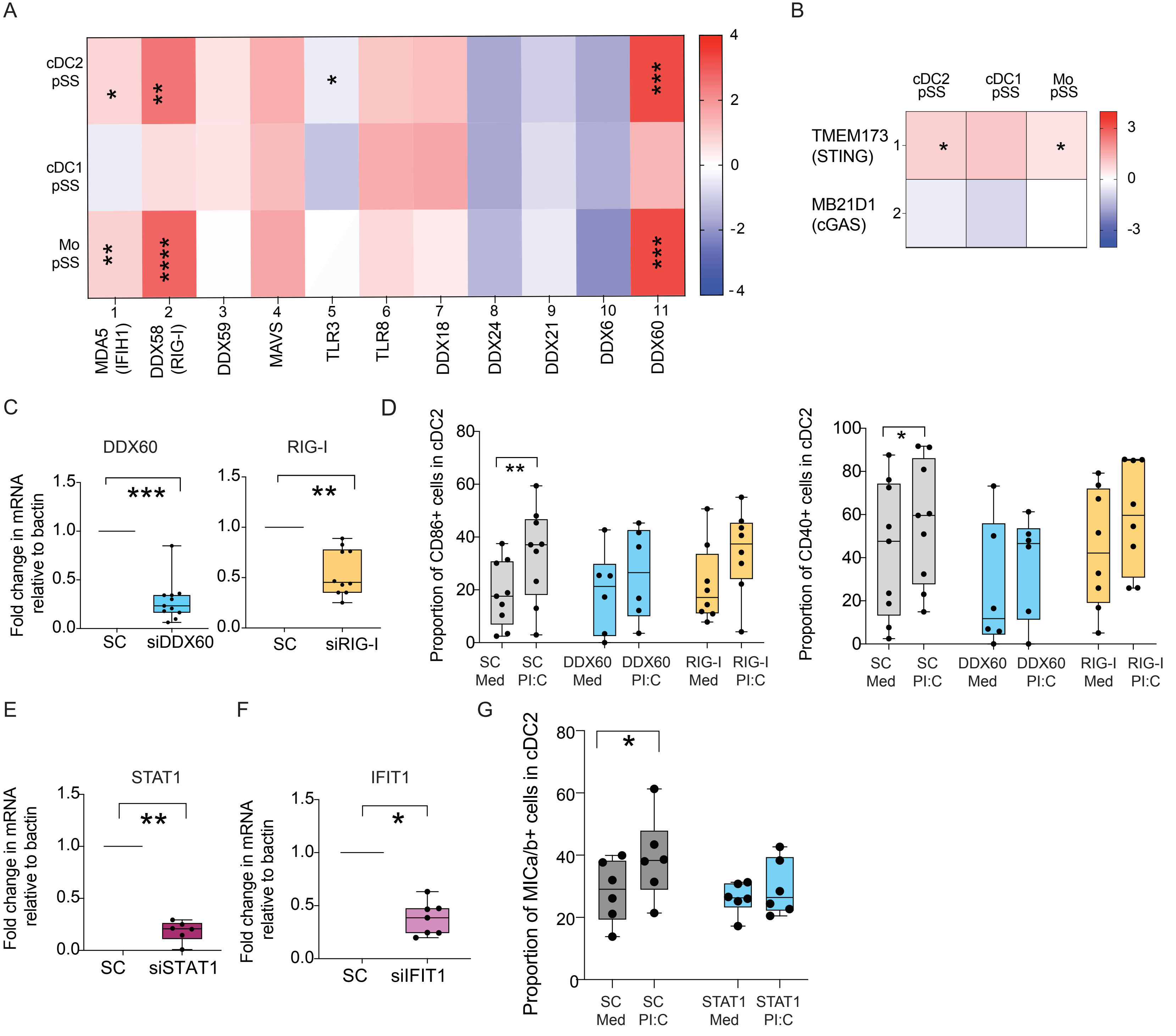

Appendix Figure S8. Expression of innate sensors and si-RNA knock down of DDX60/RIG-I and STAT1 on cDC2.

(A): Heatmap reflecting fold change in transcriptional expression of the indicated RNA helicases in cDC2, cDC1 and Mo from pSS compared to HD. Significant changes are highlighted. Red upregulated, blue downregulated.

(B): Heatmap reflecting log2FC in transcription of innate DNA sensors in cDC2 and cDC1 and in Mo of n=4 primary Sjögren´s syndrome (pSS) and n=4 healthy donor (HD).

(C, E, F): Fold change in transcriptional levels of DDX60 (C; left), DDX58 (C; RIG-I, right), STAT1 (E) and IFIT1 (F) analyzed by RT-qPCR and normalized by endogenous β-Actin levels in cDC2 nucleofected with the corresponding siRNAs. RT-qPCR values were normalized to those in cells nucleofected with control scramble (SC) siRNAs. (D, G): Proportions of cells expressing CD86 (left), CD40 (right) (D) or MICa/b within cDC2 nucleofected with either SC-siRNA (biological replicates n=9 in D and n=6 in G) or DDX60 (biological replicates n=6) or DDX58 (biological replicates n=8) (D) or STAT1 (G) (biological replicates n=6)-specific siRNAs and cultured in the presence of Med or PI:C for 16h (D). In some experiments (n=6) the impact of siRNA specific for STAT1 was also studied (G).

Data information (C, D, E, G): Data is represented as box and whiskers plots with maximum and minimum range and a median value central band. Data points are represented by biological replicates. (A,B): Statistical significant differences in differential expression in indicated genes from heatmaps is highlighted. *p<0.05; **p<0.01; ***p<0.001; ****p<0.0001.(C,D,E,F,G): Statistical significance was calculated using a two-tailed Wilcoxon matched pairs test (*p<0.05; **p<0.01; ***p<0.001).

Appendix Figure S9

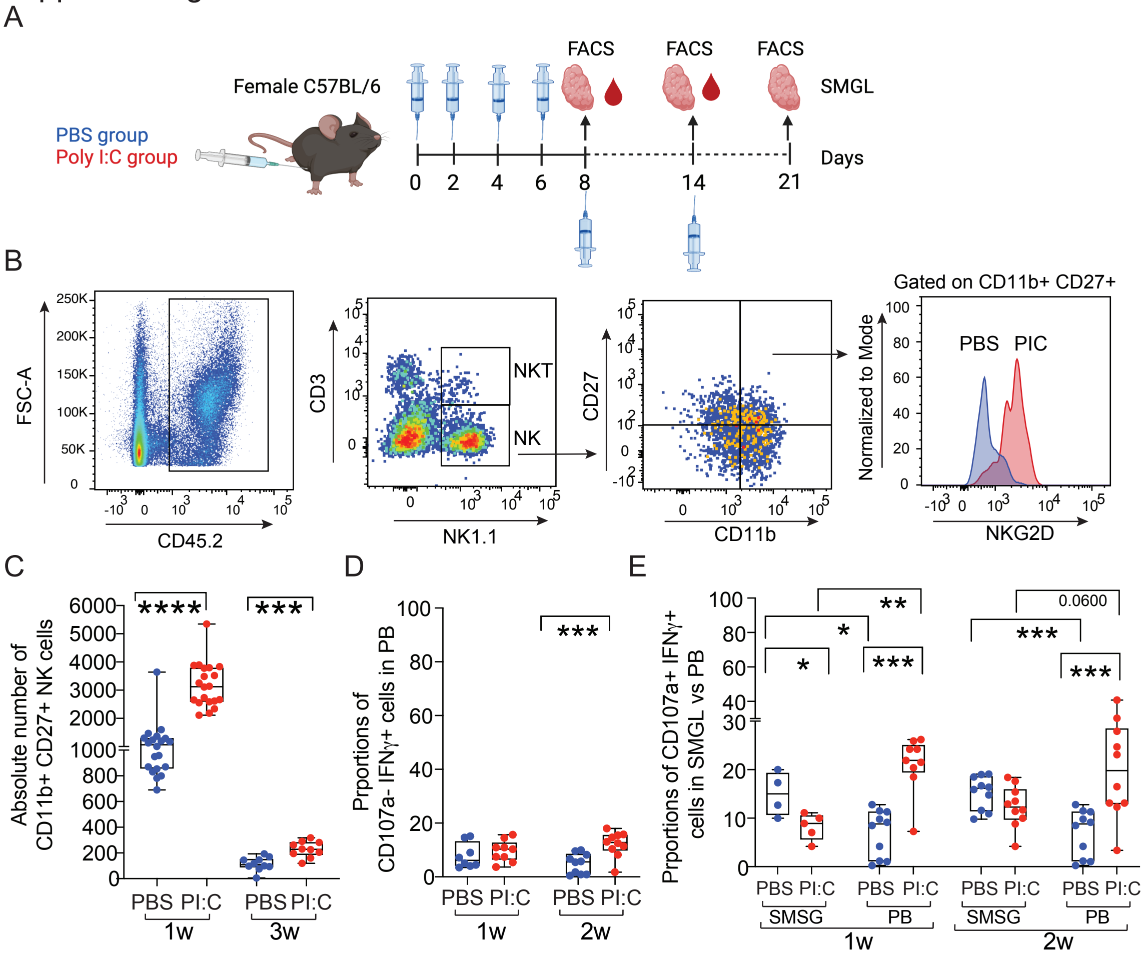

Appendix Figure S9. *In vivo* analysis of NK cells after intraperitoneal injection of poly I:C in mice.

(A): Schematic representation of the experimental design for the *in vivo* experiments where myeloid and NK cell subsets were studied in the submandibular salivary gland (SMSG) after the injection of mice with poly I:C (PI:C) or PBS.

(B): Representative flow cytometry gating strategy defining NK cell subsets in the SMSG and the expression of the indicated markers on these cells. Expression of NKG2D on gated NK cells is also shown.

(C): Absolute numbers of CD11b$^+$ CD27$^+$ cells present in the SMSG after 1 and 3 weeks post-injection with PBS (blue, biological replicates n=19 at week 1 and n=10 at week 3) and PI:C (n=19 and n=10 biological replicates at 1 and 3 weeks, respectively).

(D, E): Proportions of the CD107a$^-$ IFNγ^+ (n=8; PBS, n=9; PI:C at 1 week; n=10 PBS and PI:C at week 2)ñ in PB (D) and CD107a$^+$ IFNγ^+ cells (E) on gated CD11b$^+$ CD27$^+$ NK cell subset present in the SMSG and PB (SMSG biological replicates: n=4, n=10 in PBS at week 1 and week2; n=5 and n=10 in PI:C at week 1 and week 2; PB biological replicates: n=10 for both week 1 and 2 in PBS, n=9 and n=10 in PI:C at week 1 and 2, respectively) of the indicated experimental groups at 1 and 2 weeks after treatment initiation.

Data information (C, D, E): Data is represented as box and whiskers plots with maximum and minimum range and a median value central band. Data points are represented by biological replicates. (C,D,E): Statistical significance was calculated with a two tailed Mann Whitney test. *p<0.05; **p<0.01; ***p<0.001; ****p<0.0001.

Appendix Figure S10

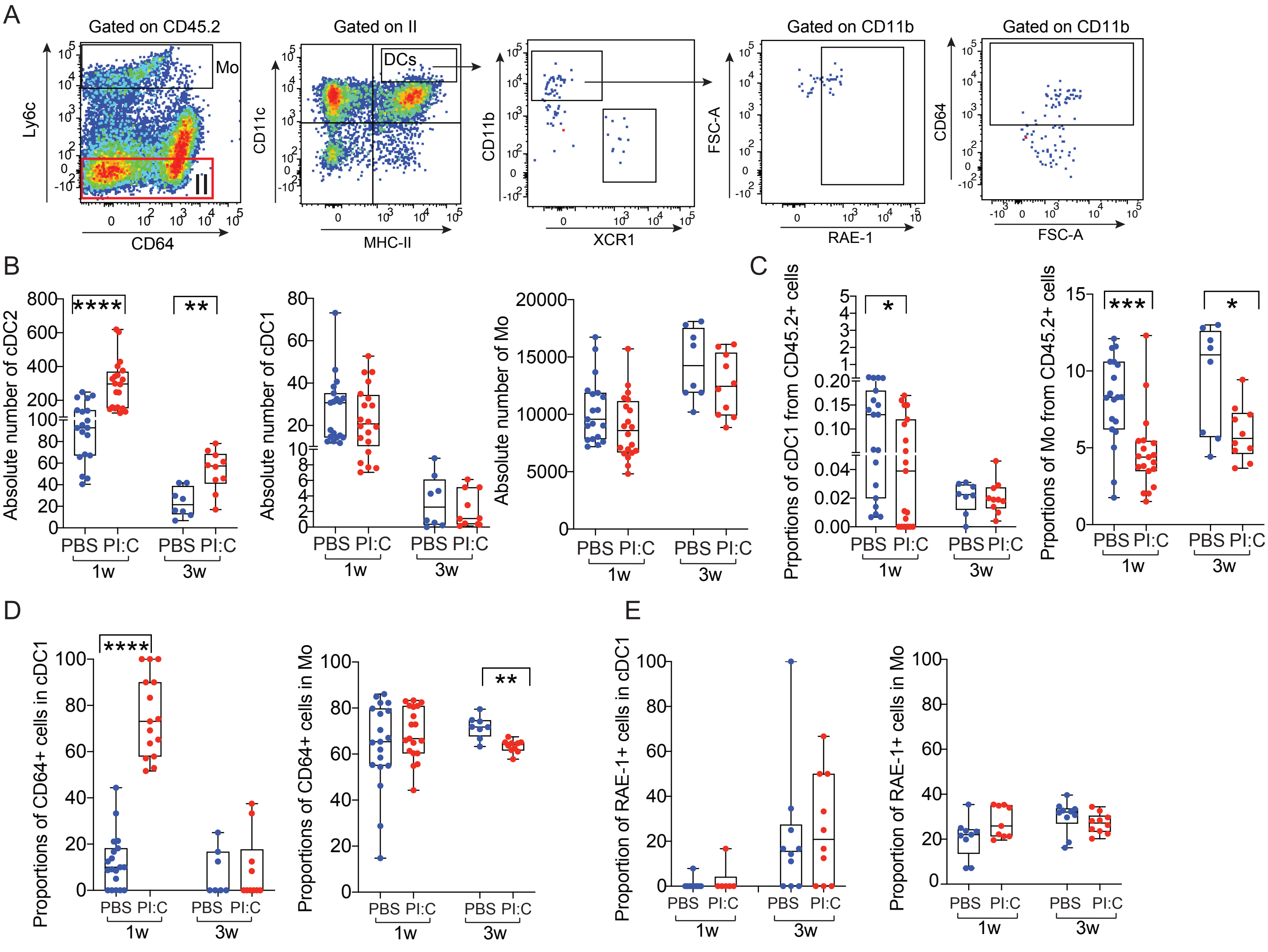

Appendix Figure S10. ***In vivo* analysis of cDC subsets and Mo after intraperitoneal injection of poly I:C in mice.**

(A): Representative flow cytometry gating strategy defining cDC1 and cDC2 subsets defined as $CD11^{hi}$ $MHC\text{-}II^{+}$ cells differing on expression of XCR1 and CD11b in the SMSG. Expression of CD64 and RAE-1 on gated cDC2 is also shown.

(B-C): Absolute numbers (B) and frequencies (C) of $XCR1^{+}$ $CD11c^{hi}$ cDC1 and $Ly6^{+}$ Mo in the SMSG of mice injected with PBS (blue) or PI:C (red) at 1 and 3 weeks after treatment initiation. Absolute numbers of cDC2 are also included. Biological replicates n=19 PBS and PI:C in B, C at 1 week and n=8 in PBS and n=10 PI:C at 3 weeks in B,C.

(D-E): Proportions of $CD64^{+}$ (n= 19 PBS and PI:C in Mo and cDC1; n=16 PI:C cDC1 at 1 week; n=8 PBS and n=10 PI:C in both subsets at 3 weeks) (D) and $RAE\text{-}I^{+}$ (n=9 PBS, n=6 PI:C cDC1; n=9 PBS, PI:C Mo at 1 week; n=10 PBS, PI:C in cDC1 and Mo at 3 weeks) (E) cells included in $XCR1^{+}$ cDC1 and $Ly6C^{+}$ Mo in the SMSG from the two mice groups at the indicated timepoints are also shown.

Data information (B, C, D, E): Data is represented as box and whiskers plots with maximum and minimum range and a median value central band. Data points are represented by biological replicates. Statistical significance was calculated with a two tailed Mann Whitney test. *$p<0.05$; **$p<0.01$; ***$p<0.001$; ****$p<0.0001$.

	HD	pSS	pSS HQ	pSS Non-HQ	OT	*p-value* HQ vs non-HQ	*p-value* HQ vs OT
**Number of patients (n)**	**56**	**48**	**17**	**25**	**6**		
Mean Age *(years, Min-Max)*	59 (25-75)	60 (23-88)	66 (44-88)	64 (23-87)	64 (42-70)	0.3667	0.2468
Sex (% Female)	100%	98%	100%	97%	100%	0.0810	n.d
**Severity n (%)**							
Mild	n.d	31 (64%)	11 (64%)	21 (84%)	0	0.0013**	<0.0001****
Severe	n.d	10 (21%)	6 (35%)	4 (16%)	6 (100%)	0.002**	<0.0001****
HQ treatment		17 (35%)	17 (100%)	0	0	<0.0001****	<0.0001****
**Extra-glandular manifestations n (%)**	n.d						
Any extra-glandular manifestations		27 (56%)	13 (76%)	8 (32%)	6 (100%)	<0.0001****	<0.0001****
Arthritis		16 (33%)	7 (41%)	6 (24%)	3 (50%)	0.0377*	0.063
Raynaud		4 (8.3%)	3 (18%)	1 (4%)	0	0.0016**	<0.0001****
Oral ulcers		1 (2.1%)	0	0	1 (16.6%)	n.d	<0.0001****
Vasculitis process		2 (4.2%)	1 (5.8%)	0	0	0.0129*	0.0129
Polyneuropathy		1 (2.1%)	0	0	1 (16.6%)	n.d	<0.0001****
Pulmonary involvement		1 (2.1%)	0	0	1 (16.6%)	n.d	<0.0001****
Kidney disease		1 (2.1%)	0	1 (3.2%)	0	0.0810	n.d
Lymphoma		2 (4.2%)	2 (11.8%)	0	0	0.0004***	0.0004***
**Laboratory values** ***Median (Min-Max)***	n.d						
RF (UI/ml)		24 (8-209)	20 (8-209)	18 (10-104)	13 (10-30)	0.3051	0.1359
ESR		27 (3-79)	29 (6-59)	31 (3-79)	17 (4-28)	0.7710	0.0455*
CRP (mg/dl)		0.16 (0.03-1.7)	0.16 (0.03-0.69)	0.18 (0-1.7)	0.1 (0.03-0,25)	0.5107	0.2931
Lymphocyte (Count/mm^3)		1545 (490-5190)	1390 (900-2480)	1565 (490-5190)	2070 (4400-9970)	0.1769	0.0023**
Gammaglobulin (g/l)		1.43 (0.41-2.79)	1.5 (0.41-2.79)	1.43 (1.05-2.68)	1.3 (0.8-1.9)	0.914	0.4826
IgG (mg/dl)		1680 (591-3430)	1930 (591-3430)	1555 (1060-2700)	1150 (877-2260)	0.2666	0.3097
C3 (mg/dl)		105 (78.9-165)	95.4 (80.7-124)	108 (57-165)	101 (81-145)	0.0306*	0.3075
C4 (mg/dl)		20 (11-29)	19.2 (13.4-33.6)	19.3 (9.1-30.6)	23 (18-29)	0.4511	0.3631

Appendix Table S1. Clinical characteristics of Sjögren´s syndrome patients

Abbreviations: HQ: Hydroxychloroquine; OT: other treatments (GC: glucocorticoids; MTX: methotrexate; B.T: biological therapy); RF: rheumatoid factor; ESR: erythrocyte sedimentation rate; CRP: C-reactive protein; IgG: immunoglobulin type G; C3: fraction 3 of complement; C4: fraction 4 of complement; n: number; n.d.= no date.

	HD	pSS	HQ pSS	Non-HQ pSS	p-value (HD vs pSS)	p-value (HD vs HQ pSS)	p-value (HD vs non-HQ pSS)	p-value (HQ vs non-HQ pSS)
Median proportions of CD56- CD16+ (min-max)	5.5 (1.3-25)	5.8 (1.2-35)	4.2 (1.2-19)	6.5 (2.4-35)	0.3654	0.9456	0.1733	0.2228
Median of CD107a on CD56- CD16+ (min-max)	12.2 (1.8-54)	22.4 (7-53)	28.6 7-53)	19.4 (10-52)	0.0479*	0.1531	0.0797	0.9039
Median of IFNγ on CD56- CD16+ (min-max)	9 (2.5-25)	15 (6-48)	14.3 (6-41)	15.5 (6-48)	0.0171*	0.0191*	0.1123	0.8404
Median of CD107a+ IFNγ+ on CD56- CD16+ (min-max)	1.5 (0-6.7)	3.3 (0-12)	3.2 (0-12)	3.5 (0.8-11)	0.085	0.5488	0.0417*	0.4778
Median of TNFα on CD56- CD16+ (min-max)	9.6 (1-50)	5.6 (0-74)	5.7 (0.2-74)	4.3 (0-58)	0.7074	0.955	0.5528	0.7242
Median of NKG2D on CD56- CD16+ (min-max)	25.5 (1.3-68)	47 (16-82)	53 (26-82)	34 (16-75)	0.0007***	<0.0001****	0.0995	0.0034**
Median of NKG2A on CD56- CD16+ (min-max)	2 (0-12)	3.3 (0.4-15)	2.9 (0.4-15)	3.45 (0.4-12)	0.0381*	0.0970	0.1054	0.8764
Median of NKp30 on CD56- CD16+ (min-max)	16.2 (1.5-58)	17.5 (2.7-55)	12.7 (3-50)	22.8 (2.7-55)	0.8032	0.1666	0.1395	0.0486*
Median of NKG2D on CD56hi CD16- (min-max)	40 (2.7-86)	49 (15-92)	59 (15-92)	46 (22-76)	0.0305*	0.0133*	0.2719	0.0913
Median of NKG2A on CD56hi CD16- (min-max)	11.5 (0-42)	14.5 (0.8-40)	13.6 (1.4-40)	16 (0.8-38)	0.1133	0.2835	0.1623	0.7712
Median of NKp30 on CD56hi CD16- (min-max)	14 (0-56)	19 (3-44)	20,3 (3-44)	16,5 (5.3-30)	0.0392*	0.0706	0.1384	0.4603

Appendix Table S2. Phenotype of different NK cell populations related with HQ (hydroxychloroquine) treatment in pSS patients. Statistical significance was calculated using a two tailed U Mann Whitney test. *p<0.05; **p<0.01; ***p<0.001; ****p<0.0001.

	HD	pSS	HQ pSS	Non-HQ pSS	p-value (HD vs pSS)	p-value (HD vs HQ pSS)	p-value (HD vs non-HQ pSS)	p-value (HQ vs non-HQ pSS)
Median proportions of CD123+ pDCs (min-max)	0.44 (0-1.3)	0.36 (0-0.9)	0.23 (0-0.9)	0.3225 (0.1-0.9)	0.2413	0.1108	0.66	0.3352
Median MFI of CD86 on pDCs (min-max)	147 (10-391)	245 (5-4080)	306 (33-4080)	166 (5-1513)	0.0451*	0.0221*	0.2506	0.5305
Median MFI of PDL-1 on pDCs (min-max)	12 (0.4-84)	20.6 (0.2-91)	8.2 (0.8-48)	27.4 (0.2-91)	0.044*	0.9779	0.0033**	0.0345*
Median MFI of CD40 on pDCs (min-max)	389 (151-558)	457 (149-724)	471 (149-724)	409 (268-724)	0.1798	0.5556	0.1387	0.4952
Median MFI of CD64 on pDCs (min-max)	66 (12-574)	200 (62-685)	246,5 (63-516)	200 (62-685)	<0.0001****	0.001**	0.0004***	0.9670
Median proportions of T Mo (min-max)	0.4 (0-7)	0.4 (0-3.5)	0.4 (0.1-3.5)	0.5 (0-3)	0.4783	0.5624	0.5711	0.9930
Median MFI of CD86 on T Mo (min-max)	809 (135-3371)	827 (398-4270)	689 (477-1721)	873 (398-4270)	0.7436	0.7040	0.4132	0.2882
Median MFI of PDL-1 on T Mo (min-max)	301 (70-1303)	320 (123-1453)	266 (123-639)	388 (141-1453)	0.1597	0.4891	0.0105*	0.0260*
Median MFI of CD40 on T Mo (min-max)	1059 (430-2275)	1309 (485-2077)	1264 (485-2077)	1309 (541-2018)	0.0414*	0.1681	0.0721	0.9272
Median MFI of CD64 on T Mo (min-max)	2919 (337-7791)	3929 (1136-9061)	4418 (1136-9061)	3409 (1298-7010)	0.0177*	0.0537	0.0661	0.9165
Median proportions of C Mo (min-max)	10,3 (3-46)	11,6 (0.8-24)	15,7 (0.8-23)	8,9 (1.1-24)	0.6347	0.1345	0.6576	0.1925
Median MFI of CD86 on C Mo (min-max)	376 (17-1976)	383 (0-3066)	363 (0-783)	452 (40-3066)	0.6435	0.5352	0.8612	0.7532
Median MFI of PDL-1 on C Mo (min-max)	423 (96-1124)	524 (134-1612)	417 (134-754)	617 (217-1612)	0.2731	0.6539	0.0623	0.0840
Median MFI of CD40 on C Mo (min-max)	770 (208-1285)	793 (288-1557)	802 (288-1557)	793 (306-1075)	0.5741	0.5476	0.7482	0.9141
Median MFI of CD64 on C Mo (min-max)	6835 (1146-19059)	8213 (1712-12940)	8644 (1712-12940)	7836 (2398-12235)	0.073	0.2159	0.1216	0.7006

Appendix Table S3. Proportion of different myeloid cell populations related with HQ (hydroxychloroquine) treatment in pSS patients.
Abbreviations: T Mo: Transitional Monocytes; C Mo: Classical Monocytes; MFI: Mean Fluorescence Intensity. Statistical significance was calculated using a two tailed U Mann Whitney test. *p<0.05; **p<0.01; ***p<0.001; ****p<0.0001.

Appendix table S4: DEGs on cDC2, cDC1 and Mo (Log2 FC cutoff upregulated >1.5; downregulated <-1.5)

DEG CD1c+ cDCs pSS *versus* HD

Transcipt ID	Gene name	Log2(FC)	FDR < 0.05
ENSG00000137959	IFI44L	4.16535789	0.00167388
ENSG00000111335	OAS2	4.15678392	0.00068553
ENSG00000182578	CSF1R	3.93436922	0.00186519
ENSG00000168329	CX3CR1	3.83812944	0.01267943
ENSG00000177409	SAMD9L	3.76415944	0.00072853
ENSG00000157601	MX1	3.7111664	0.00190217
ENSG00000089127	OAS1	3.52032768	0.00433679
ENSG00000111913	RIPOR2	3.51241416	0.0040364
ENSG00000121807	CCR2	3.51168175	0.02587417
ENSG00000132530	XAF1	3.48286431	0.00089939
ENSG00000101347	SAMHD1	3.45232655	0.00847993
ENSG00000249437	NAIP	3.42167956	0.04887038
ENSG00000111331	OAS3	3.41018922	0.00057232
ENSG00000133706	LARS	3.38958469	0.0026949
ENSG00000183486	MX2	3.32443198	0.00080111
ENSG00000140749	IGSF6	3.31354682	0.0026949
ENSG00000137628	DDX60	3.2680394	0.00045076
ENSG00000110876	SELPLG	3.24737994	0.00155933
ENSG00000139970	RTN1	3.22749388	0.00272842
ENSG00000092964	DPYSL2	3.14283953	0.00253643
ENSG00000163683	SMIM14	3.14167309	0.00668554
ENSG00000170581	STAT2	3.13212829	0.00080302
ENSG00000005020	SKAP2	3.1231954	0.00186707
ENSG00000088827	SIGLEC1	3.04532683	0.01503232
ENSG00000138449	SLC40A1	2.95156286	0.00108396
ENSG00000088986	DYNLL1	2.93772619	0.00233277
ENSG00000158488	CD1E	2.89042691	0.00562529
ENSG00000110077	MS4A6A	2.86675942	0.01645689
ENSG00000082074	FYB1	2.8576699	0.00513019
ENSG00000133835	HSD17B4	2.82579063	0.00365669
ENSG00000240065	PSMB9	2.81532891	0.00096838
ENSG00000119917	IFIT3	2.81308894	0.00098988
ENSG00000117676	RPS6KA1	2.79970312	0.00089939
ENSG00000178927	C17orf62	2.79591345	0.00088661
ENSG00000038427	VCAN	2.76743251	0.00536505
ENSG00000167220	HDHD2	2.74889964	0.00261709

ENSG00000006715	VPS41	2.73934221	0.00068553
ENSG00000107551	RASSF4	2.73092693	0.00329946
ENSG00000093072	ADA2	2.72385816	0.00251682
ENSG00000165168	CYBB	2.70586786	0.00264376
ENSG00000181381	DDX60L	2.69717824	0.00052149
ENSG00000148110	MFSD14B	2.63197719	0.00520463
ENSG00000196975	ANXA4	2.61333737	0.00077204
ENSG00000197713	RPE	2.60713676	0.01153698
ENSG00000179639	FCER1A	2.59644663	0.00882281
ENSG00000175567	UCP2	2.59370025	0.00563712
ENSG00000197043	ANXA6	2.58953409	0.00467602
ENSG00000135218	CD36	2.58500504	0.00349422
ENSG00000134326	CMPK2	2.56634923	0.00203231
ENSG00000133574	GIMAP4	2.5651703	0.02150282
ENSG00000110031	LPXN	2.55736628	0.00563329
ENSG00000188641	DPYD	2.53918064	0.00935057
ENSG00000169385	RNASE2	2.53792206	0.00498953
ENSG00000196209	SIRPB2	2.52111187	0.01585679
ENSG00000107201	DDX58	2.51544972	0.00080302
ENSG00000105281	SLC1A5	2.49745673	0.00099236
ENSG00000133943	DGLUCY	2.49512886	0.00155927
ENSG00000035403	VCL	2.47427384	0.0012446
ENSG00000170915	PAQR8	2.4699681	0.00200912
ENSG00000197142	ACSL5	2.46991698	0.00362829
ENSG00000161929	SCIMP	2.45193688	0.00709936
ENSG00000105483	CARD8	2.43912494	0.00089939
ENSG00000154930	ACSS1	2.43622234	0.00230358
ENSG00000138119	MYOF	2.4320701	0.0040802
ENSG00000165795	NDRG2	2.40671028	0.00744894
ENSG00000177575	CD163	2.39869809	0.00650305
ENSG00000146192	FGD2	2.39240703	0.00246913
ENSG00000075303	SLC25A40	2.39230879	0.00230358
ENSG00000050344	NFE2L3	2.39130111	0.00292999
ENSG00000163220	S100A9	2.38949301	0.02885702
ENSG00000165672	PRDX3	2.38019003	0.00142465
ENSG00000121281	ADCY7	2.37163873	0.00167388
ENSG00000136631	VPS45	2.37092946	0.00286187
ENSG00000188554	NBR1	2.36778573	0.0012446
ENSG00000134986	NREP	2.35723992	0.00237378
ENSG00000106785	TRIM14	2.3368806	0.00576925
ENSG00000084733	RAB10	2.32913481	0.00108396
ENSG00000184432	COPB2	2.32693742	0.00467602

ENSG00000169413	RNASE6	2.32692653	0.0010458
ENSG00000151702	FLI1	2.31890751	0.00081022
ENSG00000104974	LILRA1	2.29125844	0.00474195
ENSG00000127951	FGL2	2.2824011	0.00089939
ENSG00000166801	FAM111A	2.26969444	0.00208192
ENSG00000111269	CREBL2	2.26874304	0.00458456
ENSG00000164414	SLC35A1	2.26564961	0.0022793
ENSG00000170458	CD14	2.25623821	0.01184035
ENSG00000185745	IFIT1	2.2531576	0.00089939
ENSG00000068079	IFI35	2.25305844	0.00098988
ENSG00000258659	TRIM34	2.24478854	0.00339437
ENSG00000164125	FAM198B	2.24095776	0.01271911
ENSG00000065413	ANKRD44	2.23995368	0.00068553
ENSG00000138459	SLC35A5	2.23385815	0.00360081
ENSG00000204131	NHSL2	2.23097905	0.00406198
ENSG00000125703	ATG4C	2.23095256	0.0047099
ENSG00000107929	LARP4B	2.22788083	0.00358122
ENSG00000178685	PARP10	2.22287319	0.00015003
ENSG00000155097	ATP6V1C1	2.21598765	0.00433873
ENSG00000186088	GSAP	2.21576679	0.00261709
ENSG00000082996	RNF13	2.21087655	0.00073864
ENSG00000072501	SMC1A	2.20979185	0.00376598
ENSG00000096968	JAK2	2.20812913	0.00130443
ENSG00000198771	RCSD1	2.20709862	0.00457885
ENSG00000145781	COMMD10	2.20515315	0.00155034
ENSG00000136485	DCAF7	2.19735281	0.01586099
ENSG00000004455	AK2	2.19351353	0.0008952
ENSG00000115415	STAT1	2.17610829	0.00211531
ENSG00000102699	PARP4	2.17444696	0.00340571
ENSG00000025708	TYMP	2.17024743	0.00563582
ENSG00000103381	CPPED1	2.16814525	0.00150263
ENSG00000090861	AARS	2.16649736	0.00043805
ENSG00000093144	ECHDC1	2.16126628	0.00419863
ENSG00000143624	INTS3	2.15695705	0.01343737
ENSG00000113845	TIMMDC1	2.15354406	0.00720168
ENSG00000168310	IRF2	2.15086059	0.0041688
ENSG00000103313	MEFV	2.14612293	0.00418017
ENSG00000102893	PHKB	2.14216041	0.00131723
ENSG00000138413	IDH1	2.14150089	0.00290205
ENSG00000160593	JAML	2.14039823	0.00460845
ENSG00000145416	MARCH1	2.13486128	0.00931242
ENSG00000133106	EPSTI1	2.13206205	0.00068553

ENSG00000159228	CBR1	2.13014578	0.00045076
ENSG00000116455	WDR77	2.12930733	0.00358122
ENSG00000137965	IFI44	2.12777848	0.00876064
ENSG00000166326	TRIM44	2.1270268	0.00261709
ENSG00000163606	CD200R1	2.12284896	0.00152415
ENSG00000112367	FIG4	2.12218668	0.00167388
ENSG00000103051	COG4	2.1204803	0.00180663
ENSG00000138078	PREPL	2.12006781	0.01270208
ENSG00000173821	RNF213	2.11768767	0.00553015
ENSG00000138246	DNAJC13	2.11570284	0.00068553
ENSG00000159131	GART	2.11358372	0.00326645
ENSG00000081087	OSTM1	2.11289772	0.01186229
ENSG00000101336	HCK	2.09767392	0.00418283
ENSG00000074706	IPCEF1	2.09384519	0.02342212
ENSG00000175857	GAPT	2.08765079	0.00150263
ENSG00000095585	BLNK	2.08376627	0.00803214
ENSG00000144218	AFF3	2.08136827	0.01047324
ENSG00000136279	DBNL	2.07401784	0.00439901
ENSG00000158517	NCF1	2.06949637	0.02847331
ENSG00000166888	STAT6	2.06811372	0.00224048
ENSG00000102189	EEA1	2.06093793	0.00236146
ENSG00000280153	AC133065.6	2.06005278	0.01070935
ENSG00000163563	MNDA	2.05970748	0.0043897
ENSG00000116824	CD2	2.0576007	0.00073864
ENSG00000117054	ACADM	2.05707859	0.00152894
ENSG00000162736	NCSTN	2.05415475	0.00194739
ENSG00000150681	RGS18	2.05320806	0.00130443
ENSG00000131844	MCCC2	2.0530904	0.01886776
ENSG00000107099	DOCK8	2.0516027	0.00570959
ENSG00000198814	GK	2.04963172	0.00350313
ENSG00000148634	HERC4	2.04789706	0.00411384
ENSG00000134851	TMEM165	2.04511559	0.00084979
ENSG00000136040	PLXNC1	2.04407099	0.00885173
ENSG00000139641	ESYT1	2.02820528	0.00268199
ENSG00000048052	HDAC9	2.02033464	0.01399335
ENSG00000124357	NAGK	2.01980368	0.0012446
ENSG00000158714	SLAMF8	2.01655113	0.02493296
ENSG00000090863	GLG1	2.01630472	0.00703198
ENSG00000134321	RSAD2	2.01046868	0.00489118
ENSG00000130021	PUDP	2.00722498	0.00419863
ENSG00000031081	ARHGAP31	2.00094561	0.00235967
ENSG00000164062	APEH	1.99681356	0.00478686

ENSG00000158467	AHCYL2	1.98490934	0.01332182
ENSG00000184979	USP18	1.98276515	0.00785233
ENSG00000116990	MYCL	1.98235502	0.00516637
ENSG00000180353	HCLS1	1.98216761	0.00090802
ENSG00000121210	TMEM131L	1.98071161	0.00224427
ENSG00000142089	IFITM3	1.97921372	0.01850953
ENSG00000100364	KIAA0930	1.97310857	0.00090772
ENSG00000070785	EIF2B3	1.97182446	0.00068553
ENSG00000163565	IFI16	1.97135082	0.00322266
ENSG00000134955	SLC37A2	1.97090771	0.00376598
ENSG00000079950	STX7	1.96308556	0.00166984
ENSG00000058668	ATP2B4	1.96027503	0.00934972
ENSG00000103544	C16orf62	1.96011824	0.00248227
ENSG00000141510	TP53	1.9574839	0.00284279
ENSG00000132182	NUP210	1.94812233	0.01100861
ENSG00000188419	CHM	1.94649213	0.00235025
ENSG00000134256	CD101	1.94646613	0.0034249
ENSG00000138646	HERC5	1.94343046	0.00898743
ENSG00000169220	RGS14	1.94080353	0.00276203
ENSG00000145348	TBCK	1.93932334	0.00398077
ENSG00000155957	TMBIM4	1.93849172	0.00057232
ENSG00000134255	CEPT1	1.93725411	0.00656895
ENSG00000112118	MCM3	1.93115618	0.00553015
ENSG00000134910	STT3A	1.92914174	0.01415762
ENSG00000157637	SLC38A10	1.92596603	0.00200912
ENSG00000100030	MAPK1	1.92079694	0.00089939
ENSG00000137752	CASP1	1.91625457	0.00255612
ENSG00000156110	ADK	1.91186848	0.03702661
ENSG00000010671	BTK	1.9062616	0.00350274
ENSG00000134318	ROCK2	1.90399304	0.00430676
ENSG00000160310	PRMT2	1.9005587	0.00351578
ENSG00000130309	COLGALT1	1.90004736	0.00460345
ENSG00000165071	TMEM71	1.89529701	0.00356837
ENSG00000243943	ZNF512	1.88990246	0.00303209
ENSG00000132256	TRIM5	1.88565672	0.00229084
ENSG00000273749	CYFIP1	1.88474249	0.0066992
ENSG00000133313	CNDP2	1.88141659	0.0032195
ENSG00000178537	SLC25A20	1.88126405	0.00747632
ENSG00000138642	HERC6	1.88069728	0.00981248
ENSG00000104998	IL27RA	1.88021435	0.00089939
ENSG00000184178	SCFD2	1.87811894	0.00194739
ENSG00000150867	PIP4K2A	1.87751101	0.00205261

ENSG00000135899	SP110	1.87509223	0.00286812
ENSG00000170854	RIOX2	1.87144549	0.00472286
ENSG00000214114	MYCBP	1.87095474	0.00322031
ENSG00000156587	UBE2L6	1.86989923	0.00542396
ENSG00000059377	TBXAS1	1.86972621	0.00256622
ENSG00000187688	TRPV2	1.8686118	0.01599641
ENSG00000111540	RAB5B	1.85822044	0.00226659
ENSG00000106780	MEGF9	1.85807432	0.00309933
ENSG00000110079	MS4A4A	1.85718018	0.00107684
ENSG00000169403	PTAFR	1.85688839	0.0091414
ENSG00000104972	LILRB1	1.85246369	0.00656277
ENSG00000188404	SELL	1.84886004	0.02601302
ENSG00000204261	PSMB8-AS1	1.84595288	0.00947011
ENSG00000103423	DNAJA3	1.84202531	0.00058696
ENSG00000008869	HEATR5B	1.8416768	0.00089939
ENSG00000198951	NAGA	1.84079564	0.00525137
ENSG00000144468	RHBDD1	1.83990652	0.00652895
ENSG00000185722	ANKFY1	1.83883439	0.01278329
ENSG00000136518	ACTL6A	1.83498314	0.00330233
ENSG00000103479	RBL2	1.83345605	0.00107684
ENSG00000163644	PPM1K	1.83301931	0.00810903
ENSG00000040933	INPP4A	1.8290558	0.00351201
ENSG00000109911	ELP4	1.82900732	0.00431962
ENSG00000143799	PARP1	1.82636685	0.00888272
ENSG00000126709	IFI6	1.82601023	0.00130443
ENSG00000143252	SDHC	1.82148734	0.00109198
ENSG00000178175	ZNF366	1.81805716	0.00877547
ENSG00000161955	TNFSF13	1.8140582	0.00155034
ENSG00000213445	SIPA1	1.81355362	0.00155933
ENSG00000187608	ISG15	1.81101135	0.00349422
ENSG00000136100	VPS36	1.8056376	0.00393222
ENSG00000014216	CAPN1	1.80513921	0.00068553
ENSG00000102316	MAGED2	1.80374069	0.00599034
ENSG00000182179	UBA7	1.79549594	0.00581898
ENSG00000129675	ARHGEF6	1.79496177	0.00572497
ENSG00000143110	C1orf162	1.79134014	0.00113898
ENSG00000111667	USP5	1.78841999	0.00362829
ENSG00000111481	COPZ1	1.78766003	0.00155933
ENSG00000014919	COX15	1.78480081	0.01278329
ENSG00000196511	TPK1	1.78455658	0.00084372
ENSG00000138496	PARP9	1.78403341	0.0028424
ENSG00000168538	TRAPPC11	1.78376308	0.00095746

ENSG00000102710	SUPT20H	1.78220368	0.0016072
ENSG00000102524	TNFSF13B	1.78177857	0.0015584
ENSG00000136404	TM6SF1	1.7797517	0.01106233
ENSG00000143554	SLC27A3	1.77628553	0.00383452
ENSG00000165675	ENOX2	1.77518869	0.00531959
ENSG00000119471	HSDL2	1.77443536	0.01839835
ENSG00000166002	SMCO4	1.771189	0.00652389
ENSG00000160712	IL6R	1.76704675	0.00509474
ENSG00000243749	TMEM35B	1.76307995	0.002218
ENSG00000213983	AP1G2	1.7561938	0.02118998
ENSG00000111731	C2CD5	1.74901382	0.00089939
ENSG00000081189	MEF2C	1.74589214	0.00328847
ENSG00000108798	ABI3	1.74419429	0.00606651
ENSG00000140395	WDR61	1.74340544	0.00568
ENSG00000162704	ARPC5	1.74224952	0.00236146
ENSG00000104133	SPG11	1.7409701	0.02029751
ENSG00000088682	COQ9	1.74064775	0.00089939
ENSG00000135317	SNX14	1.74050946	0.00068553
ENSG00000149311	ATM	1.73933874	0.00246913
ENSG00000125124	BBS2	1.73821256	0.00386312
ENSG00000197798	FAM118B	1.73605372	0.01373726
ENSG00000105639	JAK3	1.73481175	0.03835979
ENSG00000105726	ATP13A1	1.73232	0.00215982
ENSG00000250138	AC139495.3	1.73142998	0.01204627
ENSG00000104365	IKBKB	1.73039626	0.00170624
ENSG00000165476	REEP3	1.72913973	0.00190829
ENSG00000140575	IQGAP1	1.72869261	0.00068553
ENSG00000189339	SLC35E2B	1.72516176	0.0086378
ENSG00000112079	STK38	1.72055039	0.00243303
ENSG00000149196	HIKESHI	1.71739712	0.00080111
ENSG00000160883	HK3	1.71050513	0.00336571
ENSG00000197157	SND1	1.71045368	0.00235967
ENSG00000134452	FBXO18	1.70474196	0.00390878
ENSG00000133103	COG6	1.70350221	0.03295002
ENSG00000172269	DPAGT1	1.7034649	0.00923715
ENSG00000071967	CYBRD1	1.70229963	0.01899945
ENSG00000169116	PARM1	1.70212386	0.00581841
ENSG00000132383	RPA1	1.70063799	0.00278533
ENSG00000121067	SPOP	1.69949896	0.00398133
ENSG00000187554	TLR5	1.6992318	0.02127473
ENSG00000163154	TNFAIP8L2	1.69414654	0.00601809
ENSG00000154822	PLCL2	1.69378163	0.00852904

ENSG00000219545	UMAD1	1.69103006	0.00229084
ENSG00000166797	FAM96A	1.690023	0.01107601
ENSG00000026751	SLAMF7	1.6850367	0.00419863
ENSG00000155926	SLA	1.68407908	0.00419863
ENSG00000030066	NUP160	1.68341977	0.0034249
ENSG00000174944	P2RY14	1.68228319	0.00359283
ENSG00000164308	ERAP2	1.68187261	0.00204546
ENSG00000198876	DCAF12	1.6788135	0.00418283
ENSG00000099810	MTAP	1.67769763	0.00688879
ENSG00000157483	MYO1E	1.67761878	0.00615876
ENSG00000104518	GSDMD	1.67618977	0.00221039
ENSG00000119922	IFIT2	1.675019	0.00402472
ENSG00000033050	ABCF2	1.67497452	0.00210312
ENSG00000163840	DTX3L	1.67472708	0.00338864
ENSG00000170006	TMEM154	1.67392517	0.0078963
ENSG00000116005	PCYOX1	1.669621	0.00077204
ENSG00000121691	CAT	1.66938691	0.00264994
ENSG00000152683	SLC30A6	1.66696901	0.02358751
ENSG00000185973	TMLHE	1.66541486	0.00719883
ENSG00000138768	USO1	1.66532486	0.00160581
ENSG00000167797	CDK2AP2	1.66381151	0.00464174
ENSG00000117228	GBP1	1.66192443	0.03832356
ENSG00000125863	MKKS	1.661671	0.00340571
ENSG00000143493	INTS7	1.66145404	0.00358122
ENSG00000256043	CTSO	1.65905713	0.00201407
ENSG00000115234	SNX17	1.65477931	0.00155933
ENSG00000139687	RB1	1.65432967	0.00599034
ENSG00000134996	OSTF1	1.65369005	0.00815818
ENSG00000124942	AHNAK	1.65160242	0.01310182
ENSG00000132646	PCNA	1.65025522	0.00075186
ENSG00000119686	FLVCR2	1.6496537	0.00409363
ENSG00000139278	GLIPR1	1.64663546	0.00668554
ENSG00000143390	RFX5	1.644153	0.0028804
ENSG00000176783	RUFY1	1.64278813	0.00019288
ENSG00000110934	BIN2	1.64169805	0.0076335
ENSG00000162946	DISC1	1.63975313	0.02702117
ENSG00000118855	MFSD1	1.63886239	0.00340003
ENSG00000115204	MPV17	1.63630852	0.00589844
ENSG00000185885	IFITM1	1.636154	0.0100445
ENSG00000130150	MOSPD2	1.63384537	0.02943076
ENSG00000103047	TANGO6	1.63139604	0.01973655
ENSG00000116701	NCF2	1.62979246	0.02288169

ENSG00000100714	MTHFD1	1.62878459	0.00744894
ENSG00000110665	C11orf21	1.62866074	0.00233277
ENSG00000006756	ARSD	1.62737992	0.00261478
ENSG00000167851	CD300A	1.62451488	0.00424199
ENSG00000186687	LYRM7	1.62448007	0.01398358
ENSG00000196510	ANAPC7	1.62292839	0.01199328
ENSG00000122025	FLT3	1.62276623	0.00503046
ENSG00000170876	TMEM43	1.62113951	0.00351394
ENSG00000104325	DECR1	1.61561589	0.00648504
ENSG00000183978	COA3	1.61537192	0.00546142
ENSG00000127946	HIP1	1.61235796	0.01311257
ENSG00000131828	PDHA1	1.61121525	0.00224048
ENSG00000138375	SMARCAL1	1.60521453	0.01682839
ENSG00000064115	TM7SF3	1.60501436	0.02874989
ENSG00000120860	WASHC3	1.59995858	0.00951642
ENSG00000181192	DHTKD1	1.59870003	0.00337093
ENSG00000101337	TM9SF4	1.59776353	0.00419863
ENSG00000124508	BTN2A2	1.59672095	0.00803266
ENSG00000163932	PRKCD	1.59396075	0.00295864
ENSG00000197548	ATG7	1.59160887	0.00155933
ENSG00000092531	SNAP23	1.59108194	0.00330233
ENSG00000128915	ICE2	1.59062394	0.00580111
ENSG00000136874	STX17	1.59054099	0.00272216
ENSG00000163389	POGLUT1	1.58939529	0.00563712
ENSG00000120451	SNX19	1.58640112	0.00126831
ENSG00000155659	VSIG4	1.58489352	0.0408237
ENSG00000137478	FCHSD2	1.58247814	0.00660914
ENSG00000160551	TAOK1	1.58239354	0.0150404
ENSG00000129691	ASH2L	1.58217542	0.00478686
ENSG00000155660	PDIA4	1.57763287	0.00398077
ENSG00000100201	DDX17	1.57737112	0.03353705
ENSG00000113522	RAD50	1.57335076	0.00398077
ENSG00000091106	NLRC4	1.57239752	0.01243906
ENSG00000124356	STAMBP	1.57147346	0.02912533
ENSG00000145246	ATP10D	1.57110413	0.01169583
ENSG00000134014	ELP3	1.57089038	0.01831573
ENSG00000142687	KIAA0319L	1.57079909	0.00272842
ENSG00000250687	AC146944.2	1.57069338	0.01618458
ENSG00000143106	PSMA5	1.57054856	0.00303209
ENSG00000106049	HIBADH	1.56946545	0.00155933
ENSG00000008130	NADK	1.56913963	0.00155933
ENSG00000173193	PARP14	1.56722447	0.04430578

ENSG00000166145	SPINT1	1.56706073	0.00261709
ENSG00000168010	ATG16L2	1.56683923	0.01188302
ENSG00000163946	FAM208A	1.56522132	0.00837266
ENSG00000139163	ETNK1	1.56452612	0.00391676
ENSG00000123106	CCDC91	1.56162639	0.00097295
ENSG00000135842	FAM129A	1.56093832	0.00961836
ENSG00000184992	BRI3BP	1.56076587	0.00653628
ENSG00000130985	UBA1	1.55856912	0.01170173
ENSG00000007923	DNAJC11	1.55848947	0.00232308
ENSG00000229754	CXCR2P1	1.55812835	0.0028754
ENSG00000132274	TRIM22	1.55293658	0.00530367
ENSG00000148180	GSN	1.55125534	0.03250746
ENSG00000124532	MRS2	1.54854827	0.00267271
ENSG00000119906	SLF2	1.54812256	0.00192901
ENSG00000138594	TMOD3	1.5475971	0.00349422
ENSG00000254505	CHMP4A	1.54643452	0.00089939
ENSG00000147251	DOCK11	1.54569043	0.00489336
ENSG00000197943	PLCG2	1.54491224	0.01603467
ENSG00000155229	MMS19	1.54354691	0.00355307
ENSG00000108946	PRKAR1A	1.54287173	0.00987036
ENSG00000136628	EPRS	1.54248327	0.00578374
ENSG00000101096	NFATC2	1.5422227	0.00697219
ENSG00000100889	PCK2	1.53930727	0.0174206
ENSG00000158481	CD1C	1.53806136	0.02980208
ENSG00000018699	TTC27	1.53740158	0.00401831
ENSG00000171307	ZDHHC16	1.53552972	0.00214395
ENSG00000185324	CDK10	1.53547738	0.00343119
ENSG00000149269	PAK1	1.53498981	0.04782639
ENSG00000151116	UEVLD	1.53468566	0.00115627
ENSG00000138363	ATIC	1.53434242	0.00548977
ENSG00000108679	LGALS3BP	1.5335672	0.00709936
ENSG00000137845	ADAM10	1.53333701	0.00648504
ENSG00000104812	GYS1	1.53254233	0.00647532
ENSG00000167272	POP5	1.53225337	0.00476653
ENSG00000109854	HTATIP2	1.53116263	0.01097056
ENSG00000113552	GNPDA1	1.52780061	0.00116354
ENSG00000189091	SF3B3	1.52570221	0.0027654
ENSG00000123213	NLN	1.52198051	0.00810903
ENSG00000138660	AP1AR	1.5204922	0.01532212
ENSG00000088888	MAVS	1.52026475	0.02330006
ENSG00000160326	SLC2A6	1.51991534	0.01121565
ENSG00000137509	PRCP	1.51757796	0.02010187

ENSG00000168710	AHCYL1	1.51740183	0.00205904
ENSG00000166986	MARS	1.51668507	0.00786406
ENSG00000156017	CARNMT1	1.51223681	0.00605285
ENSG00000134369	NAV1	1.51142591	0.01566357
ENSG00000204713	TRIM27	1.51001976	0.0028334
ENSG00000151414	NEK7	1.50940757	0.00142767
ENSG00000188352	FOCAD	1.50803856	0.0140459
ENSG00000085224	ATRX	1.50709992	0.00481717
ENSG00000100442	FKBP3	1.50707858	0.00393222
ENSG00000010292	NCAPD2	1.50618187	0.01643738
ENSG00000118961	LDAH	1.50329037	0.00088661
ENSG00000092929	UNC13D	1.50312908	0.00362829
ENSG00000108439	PNPO	1.50146753	0.00837266
ENSG00000009790	TRAF3IP3	1.50121255	0.00174835
ENSG00000136169	SETDB2	1.50094086	0.00497876
ENSG00000110931	CAMKK2	1.50039673	0.0048661
ENSG00000123689	G0S2	-7.6084168	0.02613793
ENSG00000161921	CXCL16	-4.5620958	0.01042913
ENSG00000112149	CD83	-4.5009312	0.03606944
ENSG00000095794	CREM	-4.4779198	0.01900203
ENSG00000166920	C15orf48	-4.0542904	0.01764021
ENSG00000113448	PDE4D	-3.9972163	0.02161677
ENSG00000118515	SGK1	-3.9960238	0.00395078
ENSG00000088826	SMOX	-3.9659598	0.03956602
ENSG00000120063	GNA13	-3.8727133	0.02583179
ENSG00000162496	DHRS3	-3.8716416	0.01786884
ENSG00000090104	RGS1	-3.8422815	0.02570437
ENSG00000163376	KBTBD8	-3.8282484	0.00666811
ENSG00000104312	RIPK2	-3.80965	0.00516045
ENSG00000186594	MIR22HG	-3.717995	0.00597583
ENSG00000100644	HIF1A	-3.5700622	0.00489336
ENSG00000170525	PFKFB3	-3.4828636	0.0356038
ENSG00000140379	BCL2A1	-3.4594922	0.01833318
ENSG00000122644	ARL4A	-3.4288512	0.00758077
ENSG00000130340	SNX9	-3.4221805	0.01121546
ENSG00000008083	JARID2	-3.3861327	0.01931415
ENSG00000143507	DUSP10	-3.351403	0.00758077
ENSG00000217801	AL390719.1	-3.3393149	0.04447417
ENSG00000145860	RNF145	-3.3305447	0.00043805
ENSG00000169155	ZBTB43	-3.3242159	0.00073864
ENSG00000184205	TSPYL2	-3.3190953	0.01986667
ENSG00000131669	NINJ1	-3.3057839	0.00354594

ENSG00000160223	ICOSLG	-3.244978	0.00747632
ENSG00000231721	LINC-PINT	-3.237142	0.02215288
ENSG00000173166	RAPH1	-3.2366725	0.02706525
ENSG00000255073	ZFP91-CNTF	-3.2069268	0.00316541
ENSG00000155307	SAMSN1	-3.1958956	0.01212833
ENSG00000169508	GPR183	-3.1649492	0.00084979
ENSG00000167996	FTH1	-3.1220734	0.00418283
ENSG00000153234	NR4A2	-3.1215849	0.03084488
ENSG00000113070	HBEGF	-3.1121736	0.01863785
ENSG00000173334	TRIB1	-3.1114145	0.0220503
ENSG00000154640	BTG3	-3.1046384	0.00133507
ENSG00000166128	RAB8B	-3.1014849	0.03010848
ENSG00000132906	CASP9	-3.0953133	0.03939224
ENSG00000180530	NRIP1	-3.0636904	0.00339177
ENSG00000125538	IL1B	-3.0506771	0.00688974
ENSG00000135604	STX11	-3.039988	0.0030748
ENSG00000120705	ETF1	-3.036428	0.00817205
ENSG00000011422	PLAUR	-3.0294006	0.00817205
ENSG00000272888	LINC01578	-3.0146861	0.01208127
ENSG00000165030	NFIL3	-3.0104971	0.00601809
ENSG00000168036	CTNNB1	-3.0086254	0.01162234
ENSG00000146457	WTAP	-2.9875884	0.00311935
ENSG00000277117	FP565260.3	-2.9712348	0.0112941
ENSG00000143622	RIT1	-2.9564428	0.02044292
ENSG00000102760	RGCC	-2.9555816	0.00503046
ENSG00000175130	MARCKSL1	-2.9550318	0.02165853
ENSG00000171488	LRRC8C	-2.9124449	0.00987036
ENSG00000140564	FURIN	-2.9117479	0.02813783
ENSG00000132952	USPL1	-2.8997561	0.0030748
ENSG00000169895	SYAP1	-2.8975029	0.03098433
ENSG00000132819	RBM38	-2.8929631	0.02885702
ENSG00000057657	PRDM1	-2.8814963	0.02297287
ENSG00000106004	HOXA5	-2.8736118	0.01402569
ENSG00000137936	BCAR3	-2.8427275	0.04935297
ENSG00000117036	ETV3	-2.8424885	0.04129631
ENSG00000140743	CDR2	-2.8211935	0.01278329
ENSG00000175040	CHST2	-2.8202678	0.04101553
ENSG00000197170	PSMD12	-2.8030745	0.00068553
ENSG00000211445	GPX3	-2.7908858	0.0279606
ENSG00000075426	FOSL2	-2.784013	0.02667059
ENSG00000138166	DUSP5	-2.7541356	0.02403311
ENSG00000162772	ATF3	-2.7312135	0.00375073

ENSG00000185022	MAFF	-2.7224192	0.01710678
ENSG00000114784	EIF1B	-2.7198084	0.00156522
ENSG00000186660	ZFP91	-2.7136779	0.00360138
ENSG00000110852	CLEC2B	-2.7117292	0.03716562
ENSG00000125657	TNFSF9	-2.6940337	0.00170624
ENSG00000162711	NLRP3	-2.6934593	0.02305255
ENSG00000271614	ATP2B1-AS1	-2.6879716	0.00430935
ENSG00000011566	MAP4K3	-2.6859818	0.01043629
ENSG00000277632	CCL3	-2.6812142	0.00193765
ENSG00000141682	PMAIP1	-2.6736041	0.00340571
ENSG00000025156	HSF2	-2.6655426	0.00107181
ENSG00000015475	BID	-2.632563	0.03295002
ENSG00000261740	BOLA2-SMG1P6	-2.629706	0.00122855
ENSG00000136738	STAM	-2.6137392	0.01121546
ENSG00000239305	RNF103	-2.6050891	0.00152893
ENSG00000117000	RLF	-2.604355	0.00200912
ENSG00000102908	NFAT5	-2.6031591	0.0113783
ENSG00000100600	LGMN	-2.5968497	0.04577179
ENSG00000173812	EIF1	-2.5962337	0.00204067
ENSG00000110721	CHKA	-2.581298	0.01251936
ENSG00000180628	PCGF5	-2.5780265	0.00326645
ENSG00000105835	NAMPT	-2.5580719	0.0357451
ENSG00000131408	NR1H2	-2.5564414	0.0084248
ENSG00000228830	AL160408.2	-2.5536041	0.02501364
ENSG00000122068	FYTTD1	-2.5491067	0.00096838
ENSG00000123091	RNF11	-2.5393281	0.00356837
ENSG00000147119	CHST7	-2.5367945	0.00858563
ENSG00000221869	CEBPD	-2.533836	0.00120407
ENSG00000141506	PIK3R5	-2.5294937	0.04023237
ENSG00000139832	RAB20	-2.5208739	0.01470183
ENSG00000020633	RUNX3	-2.5173964	0.00609972
ENSG00000120616	EPC1	-2.511977	0.00599216
ENSG00000156671	SAMD8	-2.5026093	0.00182038
ENSG00000118689	FOXO3	-2.4834505	0.00654052
ENSG00000112511	PHF1	-2.4821148	0.03838578
ENSG00000168264	IRF2BP2	-2.4705596	0.0088892
ENSG00000136950	ARPC5L	-2.4661936	0.00546681
ENSG00000123358	NR4A1	-2.4608666	0.00169166
ENSG00000144802	NFKBIZ	-2.460033	0.01089345
ENSG00000155252	PI4K2A	-2.4575166	0.00425322
ENSG00000126524	SBDS	-2.4403481	0.00186519

ENSG00000138069	RAB1A	-2.4313541	0.00759346
ENSG00000196843	ARID5A	-2.4206069	0.00418283
ENSG00000086062	B4GALT1	-2.4193901	0.01382481
ENSG00000175606	TMEM70	-2.4157729	0.00451031
ENSG00000105856	HBP1	-2.4083623	0.00068687
ENSG00000055483	USP36	-2.4070147	0.02957349
ENSG00000113369	ARRDC3	-2.4042168	0.02261771
ENSG00000100614	PPM1A	-2.3961858	0.00130443
ENSG00000107937	GTPBP4	-2.3954039	0.01170173
ENSG00000145675	PIK3R1	-2.3823332	0.02075998
ENSG00000171867	PRNP	-2.3817915	0.00187431
ENSG00000155304	HSPA13	-2.3758936	0.00068553
ENSG00000013441	CLK1	-2.3717733	0.00509881
ENSG00000123908	AGO2	-2.3688319	0.04186509
ENSG00000059804	SLC2A3	-2.3674552	0.03311309
ENSG00000177426	TGIF1	-2.3650867	0.00541373
ENSG00000196428	TSC22D2	-2.3621518	0.00084979
ENSG00000152484	USP12	-2.3562138	0.00628144
ENSG00000128272	ATF4	-2.3559686	0.00089939
ENSG00000087074	PPP1R15A	-2.3477231	0.01230525
ENSG00000213923	CSNK1E	-2.3443671	0.00599034
ENSG00000166165	CKB	-2.3365594	0.00191753
ENSG00000122862	SRGN	-2.3273528	0.00167388
ENSG00000124762	CDKN1A	-2.3268303	0.00479775
ENSG00000137409	MTCH1	-2.3223044	0.01442877
ENSG00000101558	VAPA	-2.3187794	0.00701573
ENSG00000171522	PTGER4	-2.3166235	0.00843969
ENSG00000025772	TOMM34	-2.315441	0.00854947
ENSG00000120690	ELF1	-2.3099373	0.00131723
ENSG00000186162	CIDECP	-2.3095705	0.00460845
ENSG00000115165	CYTIP	-2.3070301	0.0084484
ENSG00000173575	CHD2	-2.300325	0.00152893
ENSG00000151553	FAM160B1	-2.2992749	0.00272842
ENSG00000259884	AC025259.3	-2.2984853	0.00153329
ENSG00000176946	THAP4	-2.2948812	0.02164364
ENSG00000100906	NFKBIA	-2.2869631	0.00120407
ENSG00000132326	PER2	-2.284667	0.00190217
ENSG00000083799	CYLD	-2.2825129	0.00342468
ENSG00000105968	H2AFV	-2.2815598	0.00837434
ENSG00000027697	IFNGR1	-2.2789363	0.00068553
ENSG00000086666	ZFAND6	-2.2765428	0.0028622
ENSG00000108179	PPIF	-2.2745121	0.01004343

ENSG00000143153	ATP1B1	-2.2691712	0.00478686
ENSG00000114098	ARMC8	-2.2609608	0.00199149
ENSG00000141580	WDR45B	-2.2609042	0.00977231
ENSG00000166669	ATF7IP2	-2.2602164	0.02069455
ENSG00000082153	BZW1	-2.2562513	0.01364735
ENSG00000256235	SMIM3	-2.2558118	0.00772947
ENSG00000116044	NFE2L2	-2.255481	0.00359283
ENSG00000134107	BHLHE40	-2.2548603	0.00929148
ENSG00000154710	RABGEF1	-2.2496546	0.03965112
ENSG00000030110	BAK1	-2.2473536	0.04265766
ENSG00000185728	YTHDF3	-2.2411364	0.00209529
ENSG00000069956	MAPK6	-2.2409442	0.00130054
ENSG00000137331	IER3	-2.2285083	0.01475667
ENSG00000270681	AC095055.1	-2.2274383	0.00337861
ENSG00000121966	CXCR4	-2.224568	0.00709936
ENSG00000165233	CARD19	-2.2243595	0.02956604
ENSG00000176407	KCMF1	-2.220784	0.0128914
ENSG00000023734	STRAP	-2.2162293	0.01354867
ENSG00000239857	GET4	-2.2154294	0.00077204
ENSG00000176845	METRNL	-2.2127164	0.0012446
ENSG00000272886	DCP1A	-2.2052986	6.3344E-05
ENSG00000136603	SKIL	-2.203748	0.03903684
ENSG00000112245	PTP4A1	-2.2015299	0.0214355
ENSG00000102265	TIMP1	-2.1948074	0.0034608
ENSG00000079332	SAR1A	-2.190543	0.03812963
ENSG00000005483	KMT2E	-2.1903478	0.02518335
ENSG00000091317	CMTM6	-2.1902788	0.00520463
ENSG00000101544	ADNP2	-2.1847581	0.01951335
ENSG00000006451	RALA	-2.1830848	0.01575296
ENSG00000159128	IFNGR2	-2.1825726	0.00632567
ENSG00000153066	TXNDC11	-2.1784838	0.00582255
ENSG00000182831	C16orf72	-2.1781934	0.00940502
ENSG00000005379	TSPOAP1	-2.1779897	0.04328864
ENSG00000068697	LAPTM4A	-2.1749313	0.01217754
ENSG00000171604	CXXC5	-2.1725344	0.00509474
ENSG00000119801	YPEL5	-2.1686411	0.01396869
ENSG00000140941	MAP1LC3B	-2.1642957	0.00272842
ENSG00000117569	PTBP2	-2.1642283	0.01869289
ENSG00000135018	UBQLN1	-2.1582418	0.00655063
ENSG00000145241	CENPC	-2.1575822	0.02464372
ENSG00000196396	PTPN1	-2.1570102	0.03002164
ENSG00000162511	LAPTM5	-2.1549726	0.03818104

ENSG00000078269	SYNJ2	-2.1548806	0.04403393
ENSG00000163961	RNF168	-2.1499857	0.00578065
ENSG00000134480	CCNH	-2.1479935	0.02504962
ENSG00000092820	EZR	-2.1437161	0.02593285
ENSG00000106610	STAG3L4	-2.143527	0.00599034
ENSG00000065809	FAM107B	-2.1424545	0.00970995
ENSG00000151247	EIF4E	-2.141887	0.00276203
ENSG00000260708	AL118516.1	-2.1401102	0.00272216
ENSG00000169251	NMD3	-2.1376019	0.00360081
ENSG00000005812	FBXL3	-2.1372538	0.00669784
ENSG00000183696	UPP1	-2.1346803	0.0030247
ENSG00000101421	CHMP4B	-2.13348	0.0084259
ENSG00000116741	RGS2	-2.1331362	0.00388647
ENSG00000163877	SNIP1	-2.1286004	0.00340571
ENSG00000131263	RLIM	-2.1271499	0.00885186
ENSG00000182149	IST1	-2.1220026	0.00068553
ENSG00000100401	RANGAP1	-2.117229	0.01141344
ENSG00000115548	KDM3A	-2.1165221	0.00728599
ENSG00000101782	RIOK3	-2.1149049	0.00464235
ENSG00000185477	GPRIN3	-2.113831	0.00084979
ENSG00000107263	RAPGEF1	-2.1097344	0.02971227
ENSG00000184007	PTP4A2	-2.1085876	0.00362616
ENSG00000019995	ZRANB1	-2.1078433	0.00541373
ENSG00000224531	SMIM13	-2.1078034	0.02714729
ENSG00000205423	CNEP1R1	-2.1062582	0.02888384
ENSG00000130522	JUND	-2.1045991	0.00709518
ENSG00000171988	JMJD1C	-2.1019	0.00283377
ENSG00000121274	PAPD5	-2.0987985	0.03894852
ENSG00000106635	BCL7B	-2.092901	0.00023522
ENSG00000138081	FBXO11	-2.082102	0.00084979
ENSG00000184182	UBE2F	-2.0783818	0.00465281
ENSG00000140332	TLE3	-2.0771629	0.02785036
ENSG00000121671	CRY2	-2.0757999	0.015409
ENSG00000197061	HIST1H4C	-2.075254	0.02335005
ENSG00000138750	NUP54	-2.0752146	0.00345464
ENSG00000126775	ATG14	-2.0750812	0.00702311
ENSG00000078804	TP53INP2	-2.0749929	0.00215171
ENSG00000160570	DEDD2	-2.0734776	0.00300687
ENSG00000070495	JMJD6	-2.0730925	0.00020762
ENSG00000135404	CD63	-2.0723006	0.02502702
ENSG00000137947	GTF2B	-2.0715962	0.00166984
ENSG00000136732	GYPC	-2.0682301	0.00573304

ENSG00000165195	PIGA	-2.0645569	0.00842185
ENSG00000132475	H3F3B	-2.0602954	0.00669784
ENSG00000111832	RWDD1	-2.0599284	0.01295877
ENSG00000174574	AKIRIN1	-2.0497049	0.00218967
ENSG00000164823	OSGIN2	-2.0493887	0.00240547
ENSG00000117318	ID3	-2.0457496	0.03864295
ENSG00000085433	WDR47	-2.0428813	0.01756356
ENSG00000102034	ELF4	-2.0396186	0.02228775
ENSG00000101216	GMEB2	-2.039088	0.00363002
ENSG00000115520	COQ10B	-2.0383301	0.00340571
ENSG00000178381	ZFAND2A	-2.0345729	0.00503046
ENSG00000105193	RPS16	-2.0333475	0.02582355
ENSG00000164211	STARD4	-2.031064	0.04103226
ENSG00000008294	SPAG9	-2.0306064	0.01396572
ENSG00000143384	MCL1	-2.0302729	0.02313935
ENSG00000033327	GAB2	-2.0297601	0.01533113
ENSG00000241978	AKAP2	-2.0291565	0.01179582
ENSG00000185947	ZNF267	-2.0266958	0.00660914
ENSG00000006607	FARP2	-2.0249938	0.00572652
ENSG00000083937	CHMP2B	-2.0232671	0.00155933
ENSG00000183735	TBK1	-2.0229868	0.00068553
ENSG00000132823	OSER1	-2.0215438	0.00581841
ENSG00000174738	NR1D2	-2.019269	0.00174835
ENSG00000162664	ZNF326	-2.0174041	0.00088661
ENSG00000263020	AL662899.2	-2.0171997	0.00303209
ENSG00000100225	FBXO7	-2.0112347	0.002218
ENSG00000198833	UBE2J1	-2.0108671	0.02781843
ENSG00000137309	HMGA1	-2.0106825	0.01380074
ENSG00000161835	GRASP	-2.0106253	0.0239467
ENSG00000072110	ACTN1	-2.0082421	0.02476494
ENSG00000167491	GATAD2A	-2.0076679	0.0126736
ENSG00000113742	CPEB4	-2.0063751	0.00629665
ENSG00000051108	HERPUD1	-2.0029501	0.00168749
ENSG00000118503	TNFAIP3	-2.0020555	0.02209667
ENSG00000100393	EP300	-2.0018885	0.00200055
ENSG00000010072	SPRTN	-2.0009042	0.00155933
ENSG00000075415	SLC25A3	-1.9977476	0.01300954
ENSG00000141551	CSNK1D	-1.9973424	0.03828641
ENSG00000115816	CEBPZ	-1.9962322	0.00261709
ENSG00000104450	SPAG1	-1.9958727	0.00940502
ENSG00000140044	JDP2	-1.9942568	0.00710159
ENSG00000165650	PDZD8	-1.9925158	0.01583418

ENSG00000165806	CASP7	-1.9923525	0.00433347
ENSG00000121749	TBC1D15	-1.9913267	0.00073864
ENSG00000135655	USP15	-1.990058	0.00157557
ENSG00000090339	ICAM1	-1.985087	0.0011615
ENSG00000124782	RREB1	-1.9848633	0.01993667
ENSG00000187522	HSPA14	-1.984788	0.00148654
ENSG00000104969	SGTA	-1.9845314	0.00406198
ENSG00000102580	DNAJC3	-1.9826848	0.00316541
ENSG00000069849	ATP1B3	-1.9803725	0.03301678
ENSG00000107372	ZFAND5	-1.9774872	0.00335516
ENSG00000130202	NECTIN2	-1.9702677	0.04379249
ENSG00000109332	UBE2D3	-1.9694495	0.00669784
ENSG00000146232	NFKBIE	-1.9685766	0.00368511
ENSG00000067064	IDI1	-1.9666984	0.00513019
ENSG00000183484	GPR132	-1.964038	0.03115245
ENSG00000137876	RSL24D1	-1.9635082	0.00791952
ENSG00000131051	RBM39	-1.9634459	0.006914
ENSG00000117410	ATP6V0B	-1.9594605	0.00200357
ENSG00000173875	ZNF791	-1.9582482	0.00563712
ENSG00000108932	SLC16A6	-1.9581589	0.00509474
ENSG00000114120	SLC25A36	-1.9569907	0.00328847
ENSG00000119523	ALG2	-1.9567845	0.00517978
ENSG00000196704	AMZ2	-1.9557548	0.03094864
ENSG00000180611	MB21D2	-1.9555644	0.00194739
ENSG00000163874	ZC3H12A	-1.953556	0.00345283
ENSG00000113811	SELENOK	-1.9509753	0.00697219
ENSG00000182481	KPNA2	-1.9417789	0.00454367
ENSG00000139826	ABHD13	-1.9402662	0.00068553
ENSG00000104064	GABPB1	-1.9400449	0.00677138
ENSG00000134758	RNF138	-1.938487	0.00566992
ENSG00000116786	PLEKHM2	-1.9376457	0.03167434
ENSG00000140367	UBE2Q2	-1.9372058	0.00296499
ENSG00000283154	IQCJ-SCHIP1	-1.9349819	0.02389596
ENSG00000156273	BACH1	-1.9305297	0.00988027
ENSG00000117139	KDM5B	-1.9272034	0.04200804
ENSG00000150991	UBC	-1.9263115	0.01118305
ENSG00000172845	SP3	-1.9239939	0.00058696
ENSG00000111011	RSRC2	-1.9205933	0.00068553
ENSG00000124766	SOX4	-1.9189514	0.02692794
ENSG00000145780	FEM1C	-1.9187959	0.04665951
ENSG00000157954	WIPI2	-1.916411	0.00467602
ENSG00000121879	PIK3CA	-1.9146928	0.03693198

ENSG00000132912	DCTN4	-1.9131747	0.00489427
ENSG00000143761	ARF1	-1.9111358	0.00308134
ENSG00000171310	CHST11	-1.9077359	0.01618175
ENSG00000189376	C8orf76	-1.9070412	0.00652677
ENSG00000109787	KLF3	-1.9062143	0.00418283
ENSG00000159346	ADIPOR1	-1.9041438	0.00214395
ENSG00000070501	POLB	-1.9013523	0.00599034
ENSG00000267520	AC010733.2	-1.9013328	0.00224048
ENSG00000163811	WDR43	-1.9006545	0.00284279
ENSG00000057757	PITHD1	-1.8991198	0.00541373
ENSG00000178127	NDUFV2	-1.8977143	0.00068553
ENSG00000197872	FAM49A	-1.8976251	0.00642418
ENSG00000158122	AAED1	-1.8922164	0.00828958
ENSG00000183624	HMCES	-1.8915489	0.00155927
ENSG00000279766	AC067931.1	-1.8906824	0.01405517
ENSG00000125037	EMC3	-1.890385	0.00541373
ENSG00000123562	MORF4L2	-1.8875291	0.00391676
ENSG00000156535	CD109	-1.8867933	0.00907356
ENSG00000173349	SFT2D3	-1.8852676	0.00272216
ENSG00000137817	PARP6	-1.8848749	0.01481879
ENSG00000122257	RBBP6	-1.8831845	0.00570959
ENSG00000161011	SQSTM1	-1.8818954	0.00068553
ENSG00000251022	THAP9-AS1	-1.879013	0.03173612
ENSG00000197619	ZNF615	-1.8774678	0.03738591
ENSG00000164169	PRMT9	-1.8770873	0.00113486
ENSG00000162616	DNAJB4	-1.8748711	0.00197763
ENSG00000166200	COPS2	-1.8744975	0.00217078
ENSG00000160789	LMNA	-1.8728405	0.01500442
ENSG00000128271	ADORA2A	-1.8721744	0.00581841
ENSG00000162924	REL	-1.8707183	0.00562921
ENSG00000180228	PRKRA	-1.8703994	0.00068553
ENSG00000138670	RASGEF1B	-1.8692749	0.01681053
ENSG00000170852	KBTBD2	-1.8689308	0.00541373
ENSG00000130559	CAMSAP1	-1.8685161	0.04305401
ENSG00000100425	BRD1	-1.8681931	0.01639526
ENSG00000056972	TRAF3IP2	-1.8659941	0.00355307
ENSG00000196646	ZNF136	-1.8650425	0.00764292
ENSG00000168175	MAPK1IP1L	-1.8646852	0.00368155
ENSG00000139725	RHOF	-1.8612864	0.00089939
ENSG00000116670	MAD2L2	-1.860474	0.00520463
ENSG00000160799	CCDC12	-1.8604399	0.00179151
ENSG00000110046	ATG2A	-1.8574903	0.04672487

ENSG00000156232	WHAMM	-1.8571372	0.0069367
ENSG00000044574	HSPA5	-1.8566284	0.03098812
ENSG00000156875	MFSD14A	-1.8550645	0.00100563
ENSG00000127824	TUBA4A	-1.854568	0.0356038
ENSG00000144747	TMF1	-1.8533699	0.01907077
ENSG00000113575	PPP2CA	-1.845288	0.00155927
ENSG00000166822	TMEM170A	-1.8428681	0.00028968
ENSG00000114796	KLHL24	-1.8426384	0.01944808
ENSG00000122406	RPL5	-1.8421604	0.00084979
ENSG00000091527	CDV3	-1.8393821	0.01912849
ENSG00000124688	MAD2L1BP	-1.8379636	0.01436972
ENSG00000232956	SNHG15	-1.8378725	0.02908505
ENSG00000156502	SUPV3L1	-1.8376414	0.02406189
ENSG00000008056	SYN1	-1.8369916	0.03260989
ENSG00000111328	CDK2AP1	-1.8365447	0.00470819
ENSG00000181555	SETD2	-1.8349682	0.01078639
ENSG00000121741	ZMYM2	-1.8347027	0.00475524
ENSG00000070756	PABPC1	-1.83213	0.01041945
ENSG00000280138	AC027290.2	-1.8290481	0.0091546
ENSG00000105849	TWISTNB	-1.8270408	0.01340294
ENSG00000143702	CEP170	-1.8267153	0.0250423
ENSG00000067082	KLF6	-1.8252776	0.0003396
ENSG00000150787	PTS	-1.8239395	0.02885702
ENSG00000111615	KRR1	-1.8237644	0.00277722
ENSG00000149658	YTHDF1	-1.8222774	0.00068553
ENSG00000106546	AHR	-1.8195494	0.00370312
ENSG00000119977	TCTN3	-1.8157545	0.00888906
ENSG00000145779	TNFAIP8	-1.8131266	0.0141295
ENSG00000170540	ARL6IP1	-1.8129335	0.00340571
ENSG00000143514	TP53BP2	-1.8100934	0.01342164
ENSG00000153201	RANBP2	-1.8072062	0.02608793
ENSG00000133134	BEX2	-1.806631	0.0196015
ENSG00000166225	FRS2	-1.8065146	0.00647532
ENSG00000133112	TPT1	-1.8063189	0.00132049
ENSG00000106615	RHEB	-1.8038768	0.00837266
ENSG00000106346	USP42	-1.8019186	0.00190335
ENSG00000116560	SFPQ	-1.7994113	0.00068553
ENSG00000124226	RNF114	-1.7993923	0.00073864
ENSG00000109670	FBXW7	-1.7973337	0.00854947
ENSG00000004897	CDC27	-1.7965648	0.00261709
ENSG00000114013	CD86	-1.7955992	0.01681053
ENSG00000185862	EVI2B	-1.7937298	0.00456187

ENSG00000164758	MED30	-1.7926099	0.00068553
ENSG00000196233	LCOR	-1.7918862	0.00394084
ENSG00000076053	RBM7	-1.790178	0.00425431
ENSG00000280254	AC233723.2	-1.7897342	0.02069455
ENSG00000177733	HNRNPA0	-1.7892427	0.01337033
ENSG00000169826	CSGALNACT2	-1.7868556	0.0020197
ENSG00000196850	PPTC7	-1.7814752	0.00205451
ENSG00000108960	MMD	-1.7806926	0.00622658
ENSG00000114126	TFDP2	-1.780669	0.00340571
ENSG00000163605	PPP4R2	-1.7794263	0.00328847
ENSG00000111711	GOLT1B	-1.7781447	0.00057232
ENSG00000160218	TRAPPC10	-1.7731389	0.01579272
ENSG00000163682	RPL9	-1.770584	0.01545309
ENSG00000116030	SUMO1	-1.7704237	0.00175493
ENSG00000120727	PAIP2	-1.7686695	6.3344E-05
ENSG00000170881	RNF139	-1.7631297	0.03231104
ENSG00000103978	TMEM87A	-1.7616235	0.01498914
ENSG00000105851	PIK3CG	-1.7614506	0.0083323
ENSG00000163788	SNRK	-1.7602372	0.00272216
ENSG00000110848	CD69	-1.7598275	0.02816883
ENSG00000124145	SDC4	-1.7572618	0.00478686
ENSG00000135241	PNPLA8	-1.7567401	0.01448055
ENSG00000150977	RILPL2	-1.7559385	0.03826914
ENSG00000168066	SF1	-1.7517346	0.04243611
ENSG00000055208	TAB2	-1.7516409	0.02673165
ENSG00000116752	BCAS2	-1.7512089	0.00270338
ENSG00000105993	DNAJB6	-1.7510581	0.0028536
ENSG00000150907	FOXO1	-1.7504125	0.0426935
ENSG00000172062	SMN1	-1.747204	0.01832629
ENSG00000204178	TMEM57	-1.7452922	0.00355307
ENSG00000269968	AC006064.4	-1.7450836	0.01134212
ENSG00000213079	SCAF8	-1.7440465	0.00647382
ENSG00000267165	CHMP1B-AS1	-1.7402032	0.0076333
ENSG00000029993	HMGB3	-1.73794	0.01056441
ENSG00000133606	MKRN1	-1.7369882	0.00251682
ENSG00000139505	MTMR6	-1.7359162	0.00753737
ENSG00000023287	RB1CC1	-1.735826	0.00898743
ENSG00000115956	PLEK	-1.735279	0.02325376
ENSG00000136826	KLF4	-1.734594	0.01122844
ENSG00000168883	USP39	-1.7340665	0.00244907
ENSG00000089818	NECAP1	-1.7339017	0.00474195
ENSG00000099985	OSM	-1.7300245	0.01313513

ENSG00000134352	IL6ST	-1.7297119	0.00557216
ENSG00000054967	RELT	-1.7292776	0.00985902
ENSG00000165782	PIP4P1	-1.7284522	0.02069455
ENSG00000048405	ZNF800	-1.7253481	0.00068553
ENSG00000153914	SREK1	-1.724517	0.00758153
ENSG00000160741	CRTC2	-1.7237738	0.0356038
ENSG00000153922	CHD1	-1.7229725	0.00075186
ENSG00000147604	RPL7	-1.7216834	0.00328847
ENSG00000089234	BRAP	-1.721121	0.00415779
ENSG00000204977	TRIM13	-1.7191353	0.00087585
ENSG00000144597	EAF1	-1.7172654	0.00292472
ENSG00000155508	CNOT8	-1.7169155	0.01661933
ENSG00000109113	RAB34	-1.714764	0.00170624
ENSG00000162783	IER5	-1.7133399	0.0032195
ENSG00000121797	CCRL2	-1.7130671	0.00303209
ENSG00000205937	RNPS1	-1.7113322	0.0052756
ENSG00000132334	PTPRE	-1.7103155	0.03823029
ENSG00000169612	FAM103A1	-1.7100605	0.00327181
ENSG00000114942	EEF1B2	-1.7078588	0.00398077
ENSG00000089157	RPLP0	-1.7073876	0.00803214
ENSG00000168092	PAFAH1B2	-1.7068097	0.00251682
ENSG00000143774	GUK1	-1.7052018	0.0160308
ENSG00000111647	UHRF1BP1L	-1.7049002	0.00096838
ENSG00000137714	FDX1	-1.7047534	0.00152415
ENSG00000147403	RPL10	-1.7040316	0.0012446
ENSG00000125651	GTF2F1	-1.7027506	0.00178606
ENSG00000140299	BNIP2	-1.7020802	0.00112968
ENSG00000107341	UBE2R2	-1.7018502	0.01041945
ENSG00000006634	DBF4	-1.6999703	0.00251622
ENSG00000124201	ZNFX1	-1.6994888	0.00516637
ENSG00000065978	YBX1	-1.6982792	0.00985902
ENSG00000067334	DNTTIP2	-1.6982219	0.01380074
ENSG00000140455	USP3	-1.6974653	0.04122249
ENSG00000167004	PDIA3	-1.697063	0.00356514
ENSG00000266094	RASSF5	-1.6924631	0.0089013
ENSG00000119048	UBE2B	-1.6916263	0.0013773
ENSG00000070831	CDC42	-1.6915173	0.00151997
ENSG00000136802	LRRC8A	-1.6901455	0.0114427
ENSG00000102753	KPNA3	-1.6888021	0.00088963
ENSG00000122482	ZNF644	-1.6876147	0.01319879
ENSG00000065548	ZC3H15	-1.6864485	0.0069367
ENSG00000182899	RPL35A	-1.6862025	0.00217078

ENSG00000104765	BNIP3L	-1.684738	0.00647532
ENSG00000183876	ARSI	-1.6839177	0.01520729
ENSG00000173020	GRK2	-1.683088	0.00473792
ENSG00000136807	CDK9	-1.6816099	0.01764539
ENSG00000179361	ARID3B	-1.6809762	0.00476653
ENSG00000114125	RNF7	-1.6808329	0.00599034
ENSG00000162910	MRPL55	-1.6802915	0.04284787
ENSG00000156650	KAT6B	-1.6791182	0.00085958
ENSG00000141753	IGFBP4	-1.6786195	0.03066234
ENSG00000152056	AP1S3	-1.6784011	0.04880748
ENSG00000110367	DDX6	-1.6782528	0.00345296
ENSG00000108175	ZMIZ1	-1.676447	0.00843969
ENSG00000197021	CXorf40B	-1.6763325	0.00897301
ENSG00000168209	DDIT4	-1.6762806	0.00955337
ENSG00000149806	FAU	-1.6757868	0.0029323
ENSG00000163125	RPRD2	-1.6743337	0.01278329
ENSG00000241839	PLEKHO2	-1.6742498	0.00152415
ENSG00000205189	ZBTB10	-1.6738141	0.00605285
ENSG00000163694	RBM47	-1.6728334	0.00362829
ENSG00000111640	GAPDH	-1.6727812	0.00278533
ENSG00000170185	USP38	-1.67206	0.00898494
ENSG00000122026	RPL21	-1.6702654	0.00373954
ENSG00000165006	UBAP1	-1.669239	0.01424953
ENSG00000197063	MAFG	-1.6665465	0.00358122
ENSG00000105821	DNAJC2	-1.6665112	0.01220964
ENSG00000176624	MEX3C	-1.6639347	0.00365743
ENSG00000179119	SPTY2D1	-1.6635058	0.02607218
ENSG00000157540	DYRK1A	-1.661791	0.0124965
ENSG00000089737	DDX24	-1.6611366	0.00482535
ENSG00000084463	WBP11	-1.6609711	0.00066481
ENSG00000165527	ARF6	-1.6604177	0.00847993
ENSG00000082515	MRPL22	-1.657823	0.00543849
ENSG00000130803	ZNF317	-1.6575024	0.00155933
ENSG00000138433	CIR1	-1.6543325	0.00992425
ENSG00000172766	NAA16	-1.6542532	0.01220964
ENSG00000198612	COPS8	-1.652776	0.02661358
ENSG00000172239	PAIP1	-1.649347	0.00383452
ENSG00000163660	CCNL1	-1.6462447	0.02582355
ENSG00000188994	ZNF292	-1.6460525	0.04161115
ENSG00000129484	PARP2	-1.6455113	0.00479604
ENSG00000148344	PTGES	-1.6441474	0.00795236
ENSG00000091164	TXNL1	-1.6430885	0.02273887

ENSG00000185883	ATP6V0C	-1.6425553	0.04974898
ENSG00000089693	MLF2	-1.6419361	0.0015584
ENSG00000278311	GGNBP2	-1.6397228	0.01062296
ENSG00000188229	TUBB4B	-1.6380862	0.01331674
ENSG00000101247	NDUFAF5	-1.637469	0.007107
ENSG00000185043	CIB1	-1.6365419	0.0034608
ENSG00000187109	NAP1L1	-1.6353027	0.00664721
ENSG00000112031	MTRF1L	-1.6344423	0.00174835
ENSG00000196235	SUPT5H	-1.6337015	0.01375455
ENSG00000145425	RPS3A	-1.6335388	0.00437009
ENSG00000106608	URGCP	-1.6330278	0.00151997
ENSG00000115339	GALNT3	-1.6320953	0.00803214
ENSG00000177374	HIC1	-1.630597	0.02117842
ENSG00000137818	RPLP1	-1.6292029	0.0028804
ENSG00000113615	SEC24A	-1.6286771	0.02259519
ENSG00000022840	RNF10	-1.6246667	0.00643481
ENSG00000153561	RMND5A	-1.6235615	0.00261709
ENSG00000119725	ZNF410	-1.6234925	0.01184035
ENSG00000137154	RPS6	-1.6228355	0.00456305
ENSG00000128524	ATP6V1F	-1.62182	0.00085232
ENSG00000177879	AP3S1	-1.6213965	0.0254258
ENSG00000071462	BUD23	-1.6206945	0.0150404
ENSG00000156482	RPL30	-1.6202439	0.00454367
ENSG00000163041	H3F3A	-1.6200579	0.00641944
ENSG00000181467	RAP2B	-1.6196627	0.0173468
ENSG00000080546	SESN1	-1.6188649	0.02271425
ENSG00000130734	ATG4D	-1.6179839	0.00089939
ENSG00000099968	BCL2L13	-1.6155117	0.01939163
ENSG00000134453	RBM17	-1.6148427	0.00267271
ENSG00000138032	PPM1B	-1.6147717	0.02622642
ENSG00000198431	TXNRD1	-1.6147491	0.00264376
ENSG00000144713	RPL32	-1.614093	0.00472286
ENSG00000225648	SBDSP1	-1.6135219	0.00088661
ENSG00000272379	AL008729.2	-1.612116	0.00375779
ENSG00000124209	RAB22A	-1.612076	0.02643206
ENSG00000100083	GGA1	-1.6112479	0.00467105
ENSG00000008952	SEC62	-1.6097533	0.00174835
ENSG00000100316	RPL3	-1.6091778	0.00215171
ENSG00000255198	SNHG9	-1.6058942	0.00398077
ENSG00000094975	SUCO	-1.6049018	0.01955516
ENSG00000184014	DENND5A	-1.6044952	0.03763219
ENSG00000143771	CNIH4	-1.6030478	0.02227966

ENSG00000095574	IKZF5	-1.6022235	0.00553015
ENSG00000113580	NR3C1	-1.601731	0.00098422
ENSG00000009307	CSDE1	-1.6014377	0.00136992
ENSG00000179456	ZBTB18	-1.6012679	0.01112659
ENSG00000112033	PPARD	-1.5986873	0.0494711
ENSG00000181220	ZNF746	-1.5982507	0.00606651
ENSG00000172071	EIF2AK3	-1.5962497	0.01874206
ENSG00000124575	HIST1H1D	-1.5959148	0.04985787
ENSG00000109475	RPL34	-1.5953481	0.00581898
ENSG00000148572	NRBF2	-1.5924814	0.01118305
ENSG00000147526	TACC1	-1.5924675	0.02927448
ENSG00000156508	EEF1A1	-1.5924164	0.00690199
ENSG00000178623	GPR35	-1.5917165	0.03932732
ENSG00000134153	EMC7	-1.5912327	0.00230358
ENSG00000162928	PEX13	-1.5910762	0.00068553
ENSG00000124198	ARFGEF2	-1.5903358	0.02438605
ENSG00000142534	RPS11	-1.5880777	0.00479775
ENSG00000174437	ATP2A2	-1.5879063	0.00895721
ENSG00000184203	PPP1R2	-1.5867929	0.00112968
ENSG00000175390	EIF3F	-1.5859656	0.00261709
ENSG00000176142	TMEM39A	-1.585872	0.00174835
ENSG00000197019	SERTAD1	-1.5846568	0.00131723
ENSG00000135269	TES	-1.5840686	0.00478686
ENSG00000198369	SPRED2	-1.5840124	0.02118998
ENSG00000139697	SBNO1	-1.583608	0.0052464
ENSG00000083896	YTHDC1	-1.5835034	0.00816851
ENSG00000188846	RPL14	-1.5809511	0.00131723
ENSG00000148926	ADM	-1.5806023	0.00115627
ENSG00000129351	ILF3	-1.5801779	0.04150351
ENSG00000123066	MED13L	-1.5795238	0.01554896
ENSG00000125633	CCDC93	-1.5790041	0.02193221
ENSG00000119682	AREL1	-1.5788068	0.02024358
ENSG00000173960	UBXN2A	-1.5769502	0.0077505
ENSG00000140988	RPS2	-1.5765242	0.00180611
ENSG00000069399	BCL3	-1.5752995	0.0040364
ENSG00000172216	CEBPB	-1.5741391	0.03113618
ENSG00000198355	PIM3	-1.5733536	0.00854947
ENSG00000123728	RAP2C	-1.5728618	0.00188356
ENSG00000071082	RPL31	-1.5715256	0.00356514
ENSG00000125835	SNRPB	-1.5710062	0.00584684
ENSG00000168298	HIST1H1E	-1.5709735	0.03816405
ENSG00000086598	TMED2	-1.5708252	0.00433347

ENSG00000117155	SSX2IP	-1.5701526	0.00746441
ENSG00000156735	BAG4	-1.5699471	0.02812648
ENSG00000169100	SLC25A6	-1.5696587	0.00084979
ENSG00000177954	RPS27	-1.5682295	0.00536505
ENSG00000167173	C15orf39	-1.5673275	0.00145819
ENSG00000148154	UGCG	-1.5669782	0.01115489
ENSG00000115806	GORASP2	-1.5656612	0.00533443
ENSG00000175348	TMEM9B	-1.5653281	0.00068553
ENSG00000104388	RAB2A	-1.5634659	0.00454367
ENSG00000112312	GMNN	-1.5628654	0.04916348
ENSG00000143256	PFDN2	-1.5606849	0.01221599
ENSG00000146676	PURB	-1.5606155	0.03868251
ENSG00000135956	TMEM127	-1.5603145	0.01043618
ENSG00000173846	PLK3	-1.559521	0.00138775
ENSG00000115540	MOB4	-1.5589761	0.00261709
ENSG00000101084	C20orf24	-1.5589552	0.0078963
ENSG00000113013	HSPA9	-1.5586943	0.00945729
ENSG00000171863	RPS7	-1.558037	0.00898494
ENSG00000058729	RIOK2	-1.5579677	0.02601539
ENSG00000173726	TOMM20	-1.5566251	0.00752548
ENSG00000188647	PTAR1	-1.5561199	0.01520729
ENSG00000169567	HINT1	-1.5556169	0.00073864
ENSG00000142227	EMP3	-1.5538489	0.00110322
ENSG00000135486	HNRNPA1	-1.5535808	0.00068553
ENSG00000265681	RPL17	-1.5528705	0.00084979
ENSG00000231500	RPS18	-1.5528442	0.00803214
ENSG00000111897	SERINC1	-1.5522296	0.00419863
ENSG00000161526	SAP30BP	-1.5518241	0.00669784
ENSG00000166747	AP1G1	-1.550922	0.0048531
ENSG00000132155	RAF1	-1.5505873	0.00977231
ENSG00000186468	RPS23	-1.5496541	0.00384737
ENSG00000134108	ARL8B	-1.5491257	0.01646059
ENSG00000059728	MXD1	-1.5487421	0.03200845
ENSG00000164587	RPS14	-1.5477543	0.01154483
ENSG00000026508	CD44	-1.5456982	0.0344454
ENSG00000243147	MRPL33	-1.545268	0.00340571
ENSG00000132510	KDM6B	-1.544837	0.00655725
ENSG00000137393	RNF144B	-1.5439776	0.04668784
ENSG00000112137	PHACTR1	-1.5429751	0.00174835
ENSG00000167526	RPL13	-1.5426482	0.0010458
ENSG00000182827	ACBD3	-1.5424162	0.00211387
ENSG00000152700	SAR1B	-1.5422892	0.00107147

ENSG00000087460	GNAS	-1.5407708	0.00266233
ENSG00000165637	VDAC2	-1.5403825	0.00760268
ENSG00000137815	RTF1	-1.540021	0.01007801
ENSG00000115128	SF3B6	-1.5393517	0.00553015
ENSG00000101367	MAPRE1	-1.5383761	0.01752544
ENSG00000197958	RPL12	-1.5382434	0.0024099
ENSG00000104626	ERI1	-1.5379124	0.04510053
ENSG00000104267	CA2	-1.5370192	0.01999961
ENSG00000198925	ATG9A	-1.5357	0.01311257
ENSG00000147677	EIF3H	-1.5353354	0.00085958
ENSG00000241343	RPL36A	-1.5333985	0.00294771
ENSG00000145592	RPL37	-1.5323838	0.00478686
ENSG00000117614	SYF2	-1.5302778	0.00333758
ENSG00000036257	CUL3	-1.5298442	0.00194739
ENSG00000047634	SCML1	-1.5298198	0.02908495
ENSG00000141030	COPS3	-1.5291822	0.00261709
ENSG00000138231	DBR1	-1.5291675	0.00094075
ENSG00000054523	KIF1B	-1.5285471	0.0467038
ENSG00000112242	E2F3	-1.5285201	0.00540721
ENSG00000071243	ING3	-1.5273524	0.00428193
ENSG00000124214	STAU1	-1.5271249	0.00138775
ENSG00000171222	SCAND1	-1.5258209	0.0029729
ENSG00000072364	AFF4	-1.5256164	0.00115627
ENSG00000215472	RPL17-C18orf32	-1.5251675	0.00778627
ENSG00000169018	FEM1B	-1.5250244	0.02583179
ENSG00000100997	ABHD12	-1.52373	0.00200912
ENSG00000165312	OTUD1	-1.5229335	0.01913591
ENSG00000198918	RPL39	-1.5211883	0.0078963
ENSG00000162702	ZNF281	-1.520689	0.01220964
ENSG00000008405	CRY1	-1.5205039	0.01220964
ENSG00000139433	GLTP	-1.5192312	0.00458456
ENSG00000254772	EEF1G	-1.5186586	0.00312423
ENSG00000123595	RAB9A	-1.5186059	0.01342519
ENSG00000128016	ZFP36	-1.5183352	0.00098988
ENSG00000177600	RPLP2	-1.5180062	0.0027444
ENSG00000094841	UPRT	-1.5163391	0.01368036
ENSG00000015479	MATR3	-1.5153627	0.00107181
ENSG00000242125	SNHG3	-1.5130102	0.00155927
ENSG00000141428	C18orf21	-1.5124046	0.01076323
ENSG00000055211	GINM1	-1.5119738	0.01087427
ENSG00000175061	LRRC75A-AS1	-1.5090321	0.00774986

ENSG00000198160	MIER1	-1.5086014	0.00250324
ENSG00000155545	MIER3	-1.5083991	0.02110531
ENSG00000185359	HGS	-1.5074004	0.014456
ENSG00000198242	RPL23A	-1.5067581	0.00183922
ENSG00000198755	RPL10A	-1.5053093	0.01354723
ENSG00000142541	RPL13A	-1.5037966	0.00599034
ENSG00000137770	CTDSPL2	-1.5035044	0.00973379
ENSG00000101146	RAE1	-1.5029774	0.00898743
ENSG00000170779	CDCA4	-1.5020035	0.02440814
ENSG00000111666	CHPT1	-1.50194	0.00232828
ENSG00000099381	SETD1A	-1.5018804	0.00454404

DEG CD141+ cDCs pSS *versus* HD

Transcipt ID	Gene name	Log2(FC)	FDR < 0.05
ENSG00000110876	SELPLG	3.82802031	0.01263788
ENSG00000005020	SKAP2	3.68966628	0.01433947
ENSG00000132530	XAF1	3.60076426	0.01959496
ENSG00000101347	SAMHD1	3.54846982	0.00530244
ENSG00000197713	RPE	3.49566248	0.00231608
ENSG00000196975	ANXA4	3.44180679	0.00373194
ENSG00000163683	SMIM14	3.41399309	0.00329769
ENSG00000240065	PSMB9	3.34825852	0.00771252
ENSG00000177409	SAMD9L	3.34415245	0.04700928
ENSG00000151702	FLI1	3.26794279	0.0071252
ENSG00000088986	DYNLL1	3.20207297	0.00718932
ENSG00000197142	ACSL5	3.18105019	0.01686697
ENSG00000131203	IDO1	3.17237199	0.04053815
ENSG00000131844	MCCC2	3.08943003	0.00809412
ENSG00000117676	RPS6KA1	3.08369812	0.00373378
ENSG00000178927	C17orf62	3.01908121	0.00231608
ENSG00000178175	ZNF366	3.00669325	0.01156951
ENSG00000175567	UCP2	2.97576587	0.00942001
ENSG00000167797	CDK2AP2	2.97169671	0.00695581
ENSG00000092964	DPYSL2	2.9662081	0.01733544
ENSG00000133835	HSD17B4	2.95560908	0.00373194
ENSG00000004455	AK2	2.88436107	0.00450671
ENSG00000184432	COPB2	2.85168951	0.00373194
ENSG00000073849	ST6GAL1	2.84253331	0.04936695
ENSG00000108946	PRKAR1A	2.82726055	0.00785938
ENSG00000157601	MX1	2.81221268	0.00643515
ENSG00000117054	ACADM	2.8098934	0.01038122
ENSG00000133943	DGLUCY	2.80765017	0.00939014
ENSG00000111335	OAS2	2.79151142	0.02409658
ENSG00000118855	MFSD1	2.77966236	0.00231608
ENSG00000111913	RIPOR2	2.77926568	0.0164224
ENSG00000188404	SELL	2.7711235	0.00683094
ENSG00000112118	MCM3	2.75448581	0.04775931
ENSG00000127951	FGL2	2.70464858	0.00471655
ENSG00000165672	PRDX3	2.68982116	0.018607
ENSG00000136518	ACTL6A	2.6770965	0.03205559

ENSG00000133106	EPSTI1	2.6728163	0.00427995
ENSG00000143390	RFX5	2.64713348	0.00411253
ENSG00000103381	CPPED1	2.64591863	0.02817193
ENSG00000116455	WDR77	2.62619068	0.00939014
ENSG00000113845	TIMMDC1	2.61924211	0.00498634
ENSG00000170581	STAT2	2.61603253	0.01890536
ENSG00000217555	CKLF	2.61565748	0.01017501
ENSG00000138459	SLC35A5	2.61037387	0.00574457
ENSG00000093072	ADA2	2.61031333	0.01118101
ENSG00000105483	CARD8	2.58053041	0.00366462
ENSG00000146192	FGD2	2.57459956	0.03185424
ENSG00000214114	MYCBP	2.54384693	0.0123091
ENSG00000111481	COPZ1	2.53491668	0.00092215
ENSG00000103018	CYB5B	2.53412838	0.03940052
ENSG00000115415	STAT1	2.51436658	0.00714668
ENSG00000103423	DNAJA3	2.48171181	0.00369871
ENSG00000133739	LRRCC1	2.47022279	0.00427995
ENSG00000058668	ATP2B4	2.46603127	0.01140717
ENSG00000136631	VPS45	2.4455971	0.00669336
ENSG00000101336	HCK	2.43572212	0.00414186
ENSG00000165934	CPSF2	2.42895524	0.02319321
ENSG00000166801	FAM111A	2.42695116	0.04149208
ENSG00000170854	RIOX2	2.42306435	0.01017501
ENSG00000082074	FYB1	2.40640034	0.01889942
ENSG00000166002	SMCO4	2.39157552	0.02742631
ENSG00000174123	TLR10	2.39131619	0.01831296
ENSG00000197471	SPN	2.38349931	0.01377676
ENSG00000132256	TRIM5	2.37573124	0.02259125
ENSG00000174500	GCSAM	2.36971573	0.02056281
ENSG00000162736	NCSTN	2.36845665	0.01972577
ENSG00000155097	ATP6V1C1	2.36729947	0.04652814
ENSG00000183486	MX2	2.3666934	0.00366462
ENSG00000181192	DHTKD1	2.36357046	0.00643515
ENSG00000154511	FAM69A	2.35841198	0.02481535
ENSG00000113522	RAD50	2.35497808	0.00375346
ENSG00000109684	CLNK	2.34232203	0.01516728
ENSG00000107551	RASSF4	2.33187847	0.02021428
ENSG00000161929	SCIMP	2.32602311	0.01801069
ENSG00000133706	LARS	2.32397223	0.00643515
ENSG00000145781	COMMD10	2.32140285	0.02056247

ENSG00000188554	NBR1	2.31683543	0.02110815
ENSG00000134910	STT3A	2.31416843	0.03073439
ENSG00000197043	ANXA6	2.30138687	0.01333465
ENSG00000100442	FKBP3	2.30137712	0.03950432
ENSG00000198951	NAGA	2.30017514	0.01778545
ENSG00000178537	SLC25A20	2.29976075	0.00683094
ENSG00000145907	G3BP1	2.28657148	0.00366462
ENSG00000159131	GART	2.27895148	0.02160911
ENSG00000137513	NARS2	2.27413426	0.0164287
ENSG00000102699	PARP4	2.26160551	0.02565202
ENSG00000108798	ABI3	2.26085742	0.0417526
ENSG00000093144	ECHDC1	2.25935241	0.01346053
ENSG00000166797	FAM96A	2.25395611	0.01382958
ENSG00000028528	SNX1	2.2481123	0.00366462
ENSG00000138073	PREB	2.23899359	0.00427995
ENSG00000196511	TPK1	2.23484766	0.00853143
ENSG00000145982	FARS2	2.23187495	0.01599822
ENSG00000197943	PLCG2	2.2254161	0.01382958
ENSG00000169220	RGS14	2.22529372	0.00471655
ENSG00000143252	SDHC	2.22432491	0.00369871
ENSG00000168310	IRF2	2.21941679	0.01142001
ENSG00000138185	ENTPD1	2.21332138	0.01889942
ENSG00000115204	MPV17	2.21312672	0.00910123
ENSG00000258659	TRIM34	2.20303708	0.02833544
ENSG00000136100	VPS36	2.20130245	0.04311957
ENSG00000068079	IFI35	2.20055085	0.00471655
ENSG00000028116	VRK2	2.19526545	0.00366462
ENSG00000138660	AP1AR	2.18304632	0.02083863
ENSG00000136279	DBNL	2.1820547	0.00897674
ENSG00000103051	COG4	2.18108613	0.01480602
ENSG00000175792	RUVBL1	2.18096182	0.01382958
ENSG00000170915	PAQR8	2.17855823	0.03259356
ENSG00000159228	CBR1	2.17642933	0.01778545
ENSG00000198771	RCSD1	2.15975098	0.03365728
ENSG00000065413	ANKRD44	2.14995458	0.00414186
ENSG00000137509	PRCP	2.14767494	0.02417588
ENSG00000180353	HCLS1	2.14375858	0.00795821
ENSG00000140968	IRF8	2.1415942	0.01040756
ENSG00000110063	DCPS	2.13097937	0.01065996
ENSG00000134152	KATNBL1	2.12442073	0.002059

ENSG00000026751	SLAMF7	2.122695	0.00414186
ENSG00000128513	POT1	2.11684276	0.03724185
ENSG00000153064	BANK1	2.11592711	0.01948002
ENSG00000166326	TRIM44	2.10872196	0.03214462
ENSG00000176087	SLC35A4	2.10613373	0.00783832
ENSG00000159063	ALG8	2.09621344	0.00772963
ENSG00000072501	SMC1A	2.0873365	0.00388963
ENSG00000130021	PUDP	2.08334842	0.02254971
ENSG00000133313	CNDP2	2.08310086	0.00674003
ENSG00000182511	FES	2.08124765	0.00585693
ENSG00000090863	GLG1	2.07175674	0.00686317
ENSG00000006715	VPS41	2.06786491	0.0058823
ENSG00000123106	CCDC91	2.06575858	0.01849331
ENSG00000149084	HSD17B12	2.06249852	0.00329769
ENSG00000014138	POLA2	2.05890883	0.03087971
ENSG00000138768	USO1	2.05724479	0.01698373
ENSG00000155660	PDIA4	2.05661756	0.01046554
ENSG00000067704	IARS2	2.05190677	0.00683094
ENSG00000167220	HDHD2	2.05170364	0.0074401
ENSG00000170876	TMEM43	2.05022357	0.01202718
ENSG00000139641	ESYT1	2.04007922	0.00706653
ENSG00000159445	THEM4	2.03370264	0.00238242
ENSG00000162704	ARPC5	2.031856	0.00621715
ENSG00000243943	ZNF512	2.02404075	0.01764812
ENSG00000075239	ACAT1	2.02336357	0.00678127
ENSG00000164308	ERAP2	2.00102837	0.01747714
ENSG00000102524	TNFSF13B	1.99467751	0.02972242
ENSG00000014216	CAPN1	1.99448283	0.00798766
ENSG00000087263	OGFOD1	1.99421872	0.01403954
ENSG00000255833	TIFAB	1.99346242	0.00212779
ENSG00000138758	SEPT11	1.99278602	0.0159471
ENSG00000135317	SNX14	1.99277301	0.02398012
ENSG00000134996	OSTF1	1.98817748	0.04949211
ENSG00000156587	UBE2L6	1.98563628	0.01659642
ENSG00000166888	STAT6	1.98342839	0.00366462
ENSG00000128340	RAC2	1.98281961	0.0291451
ENSG00000103544	C16orf62	1.97865123	0.04701596
ENSG00000130429	ARPC1B	1.96710688	0.0060948
ENSG00000088035	ALG6	1.9664866	0.03133879
ENSG00000143314	MRPL24	1.9661189	0.02259125

ENSG00000137845	ADAM10	1.96078759	0.0239978
ENSG00000128915	ICE2	1.95925998	0.0053814
ENSG00000177054	ZDHHC13	1.95914759	0.018607
ENSG00000178057	NDUFAF3	1.95822904	0.00834834
ENSG00000035687	ADSS	1.95778237	0.01565564
ENSG00000084733	RAB10	1.95772064	0.00366462
ENSG00000115234	SNX17	1.95654784	0.00450671
ENSG00000147905	ZCCHC7	1.94631911	0.03575874
ENSG00000102710	SUPT20H	1.94521725	0.00471655
ENSG00000272047	GTF2H5	1.94299036	0.00748852
ENSG00000164062	APEH	1.94196538	0.02589679
ENSG00000198736	MSRB1	1.93727756	0.01216395
ENSG00000136874	STX17	1.93569262	0.00373378
ENSG00000112367	FIG4	1.93328907	0.0499912
ENSG00000119203	CPSF3	1.93234535	0.03489201
ENSG00000108651	UTP6	1.93055903	0.02083891
ENSG00000109814	UGDH	1.9275224	0.03064301
ENSG00000120925	RNF170	1.92584144	0.01371457
ENSG00000159596	TMEM69	1.91866781	0.01698373
ENSG00000169116	PARM1	1.91831262	0.01283324
ENSG00000080189	SLC35C2	1.91532369	0.02259125
ENSG00000141510	TP53	1.91435441	0.02477928
ENSG00000138363	ATIC	1.9089075	0.01065996
ENSG00000116133	DHCR24	1.90747385	0.04834494
ENSG00000183597	TANGO2	1.90622629	0.03474393
ENSG00000121691	CAT	1.90505411	0.00366462
ENSG00000121281	ADCY7	1.90312789	0.01321415
ENSG00000101464	PIGU	1.89949586	0.00498718
ENSG00000167272	POP5	1.89909548	0.04297077
ENSG00000162600	OMA1	1.89457122	0.00563522
ENSG00000185973	TMLHE	1.89071014	0.02052985
ENSG00000256043	CTSO	1.89027423	0.03288142
ENSG00000197157	SND1	1.88915776	0.02428243
ENSG00000032389	EIPR1	1.87940006	0.01208677
ENSG00000119321	FKBP15	1.87712459	0.00538725
ENSG00000181924	COA4	1.87138696	0.04404324
ENSG00000070269	TMEM260	1.87022464	0.02813121
ENSG00000155957	TMBIM4	1.86478753	0.01382958
ENSG00000138246	DNAJC13	1.86384869	0.04149208
ENSG00000090861	AARS	1.85873057	0.01863023

ENSG00000131236	CAP1	1.85861215	0.00366462
ENSG00000004468	CD38	1.85798611	0.00715816
ENSG00000168385	SEPT2	1.85482962	0.00505093
ENSG00000110435	PDHX	1.85051266	0.0159471
ENSG00000108984	MAP2K6	1.84232405	0.0088983
ENSG00000122694	GLIPR2	1.83979317	0.00623347
ENSG00000228223	HCG11	1.8396572	0.04682276
ENSG00000172775	FAM192A	1.83064498	0.03279199
ENSG00000138796	HADH	1.82793971	0.00373378
ENSG00000184992	BRI3BP	1.82782933	0.03259356
ENSG00000174243	DDX23	1.82695117	0.01920078
ENSG00000135336	ORC3	1.82458007	0.02598878
ENSG00000196189	SEMA4A	1.82371466	0.04199847
ENSG00000000938	FGR	1.82314394	0.00782979
ENSG00000170006	TMEM154	1.8145082	0.00695581
ENSG00000085871	MGST2	1.81235608	0.02052985
ENSG00000110768	GTF2H1	1.8076399	0.04916572
ENSG00000143624	INTS3	1.80407194	0.01491734
ENSG00000100938	GMPR2	1.80191394	0.00718932
ENSG00000122033	MTIF3	1.79769178	0.03842738
ENSG00000139278	GLIPR1	1.79725051	0.02046969
ENSG00000128654	MTX2	1.79453089	0.01831296
ENSG00000213983	AP1G2	1.79205768	0.00771252
ENSG00000136270	TBRG4	1.79087807	0.01740138
ENSG00000136485	DCAF7	1.79043521	0.03743491
ENSG00000205765	C5orf51	1.78837985	0.04794577
ENSG00000181804	SLC9A9	1.78735554	0.01740138
ENSG00000165795	NDRG2	1.78565459	0.03860348
ENSG00000176102	CSTF3	1.78210992	0.00498634
ENSG00000108528	SLC25A11	1.7809832	0.0225146
ENSG00000138964	PARVG	1.77976448	0.00783112
ENSG00000077232	DNAJC10	1.77674394	0.03956014
ENSG00000182247	UBE2E2	1.77383984	0.02213213
ENSG00000120860	WASHC3	1.77325401	0.04700928
ENSG00000152683	SLC30A6	1.76954512	0.00642013
ENSG00000168906	MAT2A	1.76732256	0.0184155
ENSG00000083535	PIBF1	1.76639128	0.01986989
ENSG00000134215	VAV3	1.76456928	0.04485765
ENSG00000131374	TBC1D5	1.7600876	0.0070359
ENSG00000143106	PSMA5	1.75667886	0.00518293

ENSG00000171307	ZDHHC16	1.75581291	0.0184155
ENSG00000089063	TMEM230	1.75554914	0.02435736
ENSG00000188452	CERKL	1.75073598	0.01764523
ENSG00000073969	NSF	1.74689502	0.04337462
ENSG00000006744	ELAC2	1.7451714	0.03815034
ENSG00000138071	ACTR2	1.74218255	0.00471655
ENSG00000092531	SNAP23	1.74089869	0.02373064
ENSG00000078618	NRDC	1.73547728	0.04120458
ENSG00000149089	APIP	1.72848666	0.00695581
ENSG00000117632	STMN1	1.72686	0.02567634
ENSG00000177628	GBA	1.72678781	0.04466065
ENSG00000273749	CYFIP1	1.72137066	0.04149208
ENSG00000138413	IDH1	1.71448061	0.01517814
ENSG00000131652	THOC6	1.70927481	0.00718932
ENSG00000104325	DECR1	1.7063217	0.00086792
ENSG00000113312	TTC1	1.70394262	0.0102919
ENSG00000136628	EPRS	1.70315632	0.037056
ENSG00000088451	TGDS	1.70293082	0.04806862
ENSG00000182551	ADI1	1.69931137	0.00329769
ENSG00000096968	JAK2	1.69650899	0.04491806
ENSG00000131828	PDHA1	1.69645807	0.00471655
ENSG00000140395	WDR61	1.69609591	0.03676234
ENSG00000122986	HVCN1	1.68983788	0.04685849
ENSG00000104812	GYS1	1.6895187	0.00548871
ENSG00000110108	TMEM109	1.6848329	0.01936255
ENSG00000031081	ARHGAP31	1.68019503	0.00718932
ENSG00000188321	ZNF559	1.67943891	0.04733928
ENSG00000145348	TBCK	1.6758879	0.04543213
ENSG00000141959	PFKL	1.67301115	0.01734575
ENSG00000088205	DDX18	1.66292701	0.02034028
ENSG00000141349	G6PC3	1.65694636	0.00509902
ENSG00000249915	PDCD6	1.65438062	0.01607135
ENSG00000094914	AAAS	1.65321137	0.02178995
ENSG00000148634	HERC4	1.65091532	0.04120458
ENSG00000241553	ARPC4	1.64852211	0.00471655
ENSG00000101246	ARFRP1	1.6474074	0.01498374
ENSG00000014641	MDH1	1.64713785	0.01184988
ENSG00000204619	PPP1R11	1.64151227	0.00751565
ENSG00000117280	RAB29	1.64123066	0.02513578
ENSG00000109911	ELP4	1.63994861	0.04936695

ENSG00000127946	HIP1	1.63988273	0.02095779
ENSG00000114956	DGUOK	1.62764348	0.03247044
ENSG00000146376	ARHGAP18	1.62659247	0.00236932
ENSG00000169019	COMMD8	1.61957748	0.02882527
ENSG00000156110	ADK	1.61949922	0.00366462
ENSG00000162813	BPNT1	1.61931129	0.01918626
ENSG00000147471	PLPBP	1.61714025	0.01972577
ENSG00000120053	GOT1	1.61486095	0.00414186
ENSG00000198814	GK	1.61386071	0.00771252
ENSG00000066583	ISOC1	1.61272735	0.01836102
ENSG00000105676	ARMC6	1.61269789	0.00709631
ENSG00000163346	PBXIP1	1.61145872	0.02274656
ENSG00000156973	PDE6D	1.60933264	0.0108102
ENSG00000125703	ATG4C	1.6090627	0.02248772
ENSG00000124702	KLHDC3	1.6080931	0.00373194
ENSG00000108622	ICAM2	1.60770762	0.00809412
ENSG00000100201	DDX17	1.60554428	0.04949211
ENSG00000182179	UBA7	1.60384845	0.0287894
ENSG00000187688	TRPV2	1.60332056	0.00798925
ENSG00000149196	HIKESHI	1.59916945	0.01636258
ENSG00000162946	DISC1	1.59411755	0.00785938
ENSG00000088827	SIGLEC1	1.59338331	0.02399459
ENSG00000162735	PEX19	1.59181511	0.04044427
ENSG00000129691	ASH2L	1.59058308	0.02886473
ENSG00000168394	TAP1	1.58770237	0.03148322
ENSG00000132182	NUP210	1.58555771	0.0473165
ENSG00000175938	ORAI3	1.58502623	0.00373194
ENSG00000134452	FBXO18	1.58402095	0.00906886
ENSG00000196576	PLXNB2	1.58273003	0.04480124
ENSG00000099899	TRMT2A	1.58232477	0.02820495
ENSG00000125877	ITPA	1.58025065	0.00373194
ENSG00000170248	PDCD6IP	1.57926176	0.0080564
ENSG00000102316	MAGED2	1.57519043	0.00897674
ENSG00000136271	DDX56	1.57485191	0.00892823
ENSG00000119616	FCF1	1.57422682	0.01778545
ENSG00000147592	LACTB2	1.57350347	0.01491734
ENSG00000109572	CLCN3	1.57237796	0.03724185
ENSG00000108389	MTMR4	1.57116178	0.00885073
ENSG00000138629	UBL7	1.57055584	0.03327519
ENSG00000100714	MTHFD1	1.56665968	0.03205559

ENSG00000135457	TFCP2	1.56555175	0.002059
ENSG00000129480	DTD2	1.56436779	0.02044521
ENSG00000160310	PRMT2	1.56277539	0.04787676
ENSG00000108479	GALK1	1.56201279	0.00706653
ENSG00000100395	L3MBTL2	1.56123945	0.01666316
ENSG00000160213	CSTB	1.56071938	0.01530022
ENSG00000140262	TCF12	1.55900951	0.00366462
ENSG00000223501	VPS52	1.55486214	0.0069917
ENSG00000157637	SLC38A10	1.55228723	0.00808263
ENSG00000198252	STYX	1.55066317	0.00373194
ENSG00000180370	PAK2	1.54978257	0.00678127
ENSG00000163344	PMVK	1.54958534	0.04949211
ENSG00000124508	BTN2A2	1.54923435	0.01040756
ENSG00000204261	PSMB8-AS1	1.54647747	0.01382796
ENSG00000165609	NUDT5	1.5464016	0.01920078
ENSG00000077380	DYNC1I2	1.54433065	0.00719913
ENSG00000135624	CCT7	1.54087321	0.01530022
ENSG00000013364	MVP	1.53339149	0.01298825
ENSG00000110871	COQ5	1.53185169	0.01698373
ENSG00000131473	ACLY	1.53045419	0.02615942
ENSG00000129317	PUS7L	1.52568904	0.04483242
ENSG00000134851	TMEM165	1.52339767	0.00678127
ENSG00000033050	ABCF2	1.52272164	0.04794577
ENSG00000241468	ATP5J2	1.52189757	0.0095478
ENSG00000112079	STK38	1.5184563	0.04133962
ENSG00000223960	AC009948.1	1.51472539	0.00719913
ENSG00000085491	SLC25A24	1.51410362	0.037056
ENSG00000165476	REEP3	1.5113931	0.03067089
ENSG00000150753	CCT5	1.50788036	0.01638433
ENSG00000102309	PIN4	1.50580323	0.04940805
ENSG00000171155	C1GALT1C1	1.50396023	0.01677843
ENSG00000160551	TAOK1	1.50351125	0.04395127
ENSG00000163154	TNFAIP8L2	1.50292149	0.01607135
ENSG00000127948	POR	1.50205485	0.0473165
ENSG00000186594	MIR22HG	-4.1138605	0.037056
ENSG00000169895	SYAP1	-3.6935282	0.04888382
ENSG00000175197	DDIT3	-3.6292117	0.01786554
ENSG00000104312	RIPK2	-3.5049456	0.02687948
ENSG00000131669	NINJ1	-3.4464943	0.0095529
ENSG00000171988	JMJD1C	-3.4364109	0.02222424

ENSG00000120690	ELF1	-3.3945734	0.02594793
ENSG00000169508	GPR183	-3.3343572	0.02220983
ENSG00000154640	BTG3	-3.3100222	0.01341595
ENSG00000277117	FP565260.3	-3.2554304	0.03348651
ENSG00000160223	ICOSLG	-3.2221071	0.01516728
ENSG00000011422	PLAUR	-3.1857979	0.02259125
ENSG00000114784	EIF1B	-3.1774242	0.01366317
ENSG00000165233	CARD19	-3.0676944	0.00771252
ENSG00000120705	ETF1	-3.0238314	0.02922772
ENSG00000099860	GADD45B	-2.9778697	0.02435736
ENSG00000130340	SNX9	-2.9684615	0.03585533
ENSG00000196233	LCOR	-2.9437394	0.02052985
ENSG00000197170	PSMD12	-2.8873085	0.0159471
ENSG00000173812	EIF1	-2.8629371	0.01118101
ENSG00000169905	TOR1AIP2	-2.845181	0.02109402
ENSG00000272886	DCP1A	-2.8334997	0.00414186
ENSG00000025156	HSF2	-2.8308445	0.00136833
ENSG00000143153	ATP1B1	-2.7778394	0.00509326
ENSG00000184205	TSPYL2	-2.7635336	0.0276593
ENSG00000068697	LAPTM4A	-2.742152	0.01921875
ENSG00000143507	DUSP10	-2.7355762	0.03535037
ENSG00000146457	WTAP	-2.735327	0.01746478
ENSG00000110852	CLEC2B	-2.7079704	0.00373194
ENSG00000151012	SLC7A11	-2.705993	0.02775042
ENSG00000150907	FOXO1	-2.6787004	0.01662887
ENSG00000121966	CXCR4	-2.676161	0.0414865
ENSG00000067082	KLF6	-2.671159	0.03034903
ENSG00000134107	BHLHE40	-2.6703527	0.03259356
ENSG00000116044	NFE2L2	-2.6667114	0.04483242
ENSG00000113070	HBEGF	-2.657094	0.01651978
ENSG00000138069	RAB1A	-2.6237485	0.02565202
ENSG00000171522	PTGER4	-2.6039809	0.02407448
ENSG00000104765	BNIP3L	-2.6025565	0.01209023
ENSG00000119326	CTNNAL1	-2.5966732	0.00812086
ENSG00000114796	KLHL24	-2.5933178	0.00943543
ENSG00000135655	USP15	-2.5879499	0.01686697
ENSG00000277632	CCL3	-2.5855865	0.03558605
ENSG00000118308	LRMP	-2.5694642	0.0291451
ENSG00000173575	CHD2	-2.5502927	0.03262661
ENSG00000267520	AC010733.2	-2.5416381	0.01944561

ENSG00000120129	DUSP1	-2.5176534	0.01849331
ENSG00000136950	ARPC5L	-2.4969028	0.00848138
ENSG00000185477	GPRIN3	-2.4953763	0.00538725
ENSG00000162924	REL	-2.4769513	0.00366462
ENSG00000166822	TMEM170A	-2.474987	0.03035108
ENSG00000124782	RREB1	-2.4708018	0.00498718
ENSG00000120616	EPC1	-2.4696606	0.00719913
ENSG00000117000	RLF	-2.4630327	0.02304511
ENSG00000111615	KRR1	-2.4532643	0.01803367
ENSG00000107937	GTPBP4	-2.4521505	0.01700522
ENSG00000163961	RNF168	-2.4425438	0.01530022
ENSG00000185650	ZFP36L1	-2.436076	0.00373378
ENSG00000089818	NECAP1	-2.4218155	0.01118014
ENSG00000150977	RILPL2	-2.4151521	0.04793703
ENSG00000132952	USPL1	-2.4128638	0.02471595
ENSG00000013441	CLK1	-2.4099282	0.00574457
ENSG00000272888	LINC01578	-2.3985687	0.0071252
ENSG00000139977	NAA30	-2.3978653	0.00373194
ENSG00000105821	DNAJC2	-2.3925275	0.01118101
ENSG00000115165	CYTIP	-2.3875843	0.03287197
ENSG00000144674	GOLGA4	-2.3827561	0.00427995
ENSG00000081320	STK17B	-2.3761399	0.03823614
ENSG00000011566	MAP4K3	-2.3741461	0.01565564
ENSG00000128272	ATF4	-2.367223	0.0102919
ENSG00000133134	BEX2	-2.3659944	0.00231608
ENSG00000135404	CD63	-2.3636386	0.03593778
ENSG00000100644	HIF1A	-2.3585647	0.00643515
ENSG00000204977	TRIM13	-2.3487972	0.02488781
ENSG00000100393	EP300	-2.3299326	0.002059
ENSG00000163874	ZC3H12A	-2.3204056	0.01651978
ENSG00000189376	C8orf76	-2.3191063	0.02882527
ENSG00000102908	NFAT5	-2.31265	0.04949211
ENSG00000059728	MXD1	-2.3005657	0.04005277
ENSG00000122644	ARL4A	-2.3000878	0.0498361
ENSG00000115946	PNO1	-2.2847106	0.01651978
ENSG00000270681	AC095055.1	-2.2813465	0.01046554
ENSG00000145241	CENPC	-2.2796577	0.03259356
ENSG00000120733	KDM3B	-2.2779398	0.02350696
ENSG00000005483	KMT2E	-2.2773755	0.00952539
ENSG00000178381	ZFAND2A	-2.2759391	0.01529195

ENSG00000255198	SNHG9	-2.2736608	0.01641025
ENSG00000163605	PPP4R2	-2.264769	0.03228479
ENSG00000001561	ENPP4	-2.2537182	0.04703942
ENSG00000117569	PTBP2	-2.2519153	0.03034903
ENSG00000080822	CLDND1	-2.2506605	0.03814153
ENSG00000188647	PTAR1	-2.2470642	0.02053825
ENSG00000114098	ARMC8	-2.2421448	0.02589679
ENSG00000112137	PHACTR1	-2.2401225	0.00670978
ENSG00000115520	COQ10B	-2.2373364	0.013493
ENSG00000091164	TXNL1	-2.2326181	0.00709631
ENSG00000075415	SLC25A3	-2.2167682	0.04792734
ENSG00000153922	CHD1	-2.2050932	0.00373194
ENSG00000102580	DNAJC3	-2.2043728	0.00427995
ENSG00000047634	SCML1	-2.2033829	0.04637292
ENSG00000271614	ATP2B1-AS1	-2.2008705	0.02056281
ENSG00000117139	KDM5B	-2.1966822	0.01686697
ENSG00000100614	PPM1A	-2.1878349	0.04597372
ENSG00000136603	SKIL	-2.18575	0.02360332
ENSG00000155307	SAMSN1	-2.1854028	0.04158965
ENSG00000105968	H2AFV	-2.1838924	0.01212266
ENSG00000121741	ZMYM2	-2.1836391	0.01040756
ENSG00000161011	SQSTM1	-2.1831974	0.02504834
ENSG00000032219	ARID4A	-2.1799884	0.00498634
ENSG00000138593	SECISBP2L	-2.179669	0.02398012
ENSG00000067064	IDI1	-2.1776063	0.0095478
ENSG00000117410	ATP6V0B	-2.1766065	0.03645327
ENSG00000064012	CASP8	-2.164785	0.04923352
ENSG00000137947	GTF2B	-2.1631284	0.01651978
ENSG00000126524	SBDS	-2.1627109	0.0088983
ENSG00000087460	GNAS	-2.1617571	0.0383737
ENSG00000106615	RHEB	-2.1573998	0.01404513
ENSG00000008294	SPAG9	-2.1568151	0.03950432
ENSG00000163788	SNRK	-2.1497816	0.01688977
ENSG00000160570	DEDD2	-2.1427921	0.03185424
ENSG00000111011	RSRC2	-2.1414172	0.00366462
ENSG00000180747	SMG1P3	-2.1398988	0.04580405
ENSG00000197021	CXorf40B	-2.1371799	0.01981956
ENSG00000048405	ZNF800	-2.1367196	0.01636258
ENSG00000144747	TMF1	-2.1363366	0.00894515
ENSG00000113580	NR3C1	-2.1357775	0.02274656

ENSG00000083799	CYLD	-2.128274	0.03645327
ENSG00000213923	CSNK1E	-2.1231989	0.0088983
ENSG00000176407	KCMF1	-2.1192581	0.01565564
ENSG00000161526	SAP30BP	-2.118821	0.01633796
ENSG00000118689	FOXO3	-2.1162289	0.00414186
ENSG00000113811	SELENOK	-2.1155648	0.00366462
ENSG00000139697	SBNO1	-2.1025971	0.013493
ENSG00000135093	USP30	-2.0988346	0.02085529
ENSG00000006607	FARP2	-2.0975951	0.03960245
ENSG00000132823	OSER1	-2.0975217	0.02360332
ENSG00000165806	CASP7	-2.0951807	0.02132332
ENSG00000136451	VEZF1	-2.0882355	0.00450671
ENSG00000187837	HIST1H1C	-2.0821429	0.02440446
ENSG00000176845	METRNL	-2.080076	0.00373194
ENSG00000140743	CDR2	-2.0730612	0.02259125
ENSG00000162664	ZNF326	-2.0716828	0.00471655
ENSG00000005339	CREBBP	-2.0672713	0.0360832
ENSG00000124688	MAD2L1BP	-2.0644079	0.00231608
ENSG00000160799	CCDC12	-2.0627806	0.0184155
ENSG00000020633	RUNX3	-2.0618496	0.03365163
ENSG00000100528	CNIH1	-2.053928	0.01118014
ENSG00000138050	THUMPD2	-2.0529135	0.02922772
ENSG00000140995	DEF8	-2.0505927	0.03309042
ENSG00000115738	ID2	-2.0494412	0.01003557
ENSG00000105849	TWISTNB	-2.0375851	0.00366462
ENSG00000015568	RGPD5	-2.0328509	0.00462374
ENSG00000186162	CIDECP	-2.0315175	0.00471655
ENSG00000124226	RNF114	-2.0279585	0.00366462
ENSG00000152484	USP12	-2.024435	0.0278803
ENSG00000113163	COL4A3BP	-2.0221942	0.00366462
ENSG00000122068	FYTTD1	-2.018704	0.00695581
ENSG00000113615	SEC24A	-2.0168449	0.03950432
ENSG00000239305	RNF103	-2.0139912	0.01786554
ENSG00000074266	EED	-2.0126258	0.03279199
ENSG00000086666	ZFAND6	-2.0125353	0.00471655
ENSG00000114416	FXR1	-2.0109795	0.0164287
ENSG00000183484	GPR132	-2.0088306	0.01901204
ENSG00000070831	CDC42	-2.004092	0.00950579
ENSG00000123091	RNF11	-1.9982661	0.03288142
ENSG00000101146	RAE1	-1.997937	0.01800346

ENSG00000220205	VAMP2	-1.9935544	0.00366094
ENSG00000198780	FAM169A	-1.9880729	0.00643515
ENSG00000124177	CHD6	-1.9868299	0.03012117
ENSG00000083937	CHMP2B	-1.9863551	0.01284201
ENSG00000145779	TNFAIP8	-1.9853279	0.02833847
ENSG00000155744	FAM126B	-1.9852854	0.04698791
ENSG00000131263	RLIM	-1.9850418	0.023638
ENSG00000156650	KAT6B	-1.9847407	0.00518293
ENSG00000076554	TPD52	-1.983622	0.02230772
ENSG00000172845	SP3	-1.9789946	0.01831296
ENSG00000179833	SERTAD2	-1.9784744	0.00471655
ENSG00000108510	MED13	-1.9775367	0.00565028
ENSG00000213096	ZNF254	-1.9762201	0.01659642
ENSG00000115310	RTN4	-1.974329	0.02095779
ENSG00000083896	YTHDC1	-1.9737135	0.02845741
ENSG00000152133	GPATCH11	-1.9736697	0.04700928
ENSG00000104447	TRPS1	-1.9712435	0.0080564
ENSG00000144802	NFKBIZ	-1.9680102	0.03365163
ENSG00000101247	NDUFAF5	-1.9651746	0.02471963
ENSG00000180228	PRKRA	-1.9650885	0.01530022
ENSG00000278311	GGNBP2	-1.9647999	0.02052985
ENSG00000183604	SMG1P5	-1.9634269	0.00910301
ENSG00000101782	RIOK3	-1.9623211	0.04752992
ENSG00000197857	ZNF44	-1.9585977	0.01118101
ENSG00000008083	JARID2	-1.9565897	0.00393434
ENSG00000144566	RAB5A	-1.9552067	0.0157351
ENSG00000111832	RWDD1	-1.9549769	0.02573744
ENSG00000125651	GTF2F1	-1.9502405	0.00525643
ENSG00000112242	E2F3	-1.9492172	0.02046898
ENSG00000057657	PRDM1	-1.9490263	0.0095478
ENSG00000101596	SMCHD1	-1.9456298	0.03221962
ENSG00000135018	UBQLN1	-1.9455278	0.00450671
ENSG00000197019	SERTAD1	-1.944542	0.00373194
ENSG00000164615	CAMLG	-1.9443022	0.01565564
ENSG00000145675	PIK3R1	-1.943078	0.04297077
ENSG00000137876	RSL24D1	-1.9416112	0.01834035
ENSG00000123358	NR4A1	-1.9402544	0.0291451
ENSG00000124201	ZNFX1	-1.9402019	0.04010247
ENSG00000119801	YPEL5	-1.9398959	0.02259125
ENSG00000162923	WDR26	-1.938859	0.01323649

ENSG00000168264	IRF2BP2	-1.9382528	0.00414186
ENSG00000159346	ADIPOR1	-1.9348955	0.02589679
ENSG00000100425	BRD1	-1.9339988	0.00943543
ENSG00000101654	RNMT	-1.9267108	0.0376018
ENSG00000103121	CMC2	-1.9191775	0.01882969
ENSG00000156675	RAB11FIP1	-1.9183849	0.01401736
ENSG00000006576	PHTF2	-1.917113	0.01969645
ENSG00000147872	PLIN2	-1.91664	0.03653071
ENSG00000100852	ARHGAP5	-1.9165827	0.01922859
ENSG00000170385	SLC30A1	-1.9081168	0.0498361
ENSG00000140153	WDR20	-1.9078328	0.00643515
ENSG00000196850	PPTC7	-1.9043128	0.02750331
ENSG00000096746	HNRNPH3	-1.8950537	0.002059
ENSG00000272196	HIST2H2AA4	-1.8877796	0.01651978
ENSG00000129351	ILF3	-1.8846709	0.01824229
ENSG00000120727	PAIP2	-1.8772952	0.0467091
ENSG00000095574	IKZF5	-1.8754389	0.04020907
ENSG00000082515	MRPL22	-1.8707563	0.03228368
ENSG00000150991	UBC	-1.8689767	0.02820495
ENSG00000187522	HSPA14	-1.867056	0.00414186
ENSG00000183624	HMCES	-1.863544	0.00231608
ENSG00000027697	IFNGR1	-1.8604347	0.00518293
ENSG00000134453	RBM17	-1.8584692	0.02610459
ENSG00000173276	ZBTB21	-1.8564865	0.03259356
ENSG00000101544	ADNP2	-1.8551142	0.0322021
ENSG00000132475	H3F3B	-1.8493775	0.0108102
ENSG00000169826	CSGALNACT2	-1.8489192	0.02540537
ENSG00000184182	UBE2F	-1.8486154	0.03300659
ENSG00000146425	DYNLT1	-1.8468949	0.04820922
ENSG00000230551	AC021078.1	-1.8467533	0.00674003
ENSG00000269926	DDIT4-AS1	-1.8458646	0.04840339
ENSG00000094975	SUCO	-1.8424783	0.03515012
ENSG00000265681	RPL17	-1.8407111	0.01750196
ENSG00000101558	VAPA	-1.84037	0.04158427
ENSG00000004897	CDC27	-1.8398906	0.03650675
ENSG00000097007	ABL1	-1.8381003	0.0154968
ENSG00000106829	TLE4	-1.8359712	0.02841211
ENSG00000182149	IST1	-1.8342697	0.00718932
ENSG00000228830	AL160408.2	-1.8299424	0.00366462
ENSG00000101109	STK4	-1.8210315	0.00414186

ENSG00000138750	NUP54	-1.8166616	0.00518293
ENSG00000109466	KLHL2	-1.8165383	0.01488832
ENSG00000112773	FAM46A	-1.8155196	0.03645327
ENSG00000075426	FOSL2	-1.8140914	0.0416784
ENSG00000019995	ZRANB1	-1.812823	0.03764973
ENSG00000176624	MEX3C	-1.8101519	0.002059
ENSG00000177885	GRB2	-1.8096175	0.00678127
ENSG00000115540	MOB4	-1.8093547	0.00414186
ENSG00000196396	PTPN1	-1.8083053	0.0075056
ENSG00000163960	UBXN7	-1.8042536	0.03602727
ENSG00000140455	USP3	-1.8015012	0.04024955
ENSG00000196504	PRPF40A	-1.8012192	0.01734575
ENSG00000100483	VCPKMT	-1.8006405	0.01298825
ENSG00000008952	SEC62	-1.7986837	0.02615942
ENSG00000183735	TBK1	-1.7949466	0.03259356
ENSG00000124762	CDKN1A	-1.790485	0.03653071
ENSG00000068745	IP6K2	-1.7896868	0.0064376
ENSG00000143702	CEP170	-1.7801982	0.01863023
ENSG00000011007	ELOA	-1.77355	0.01599822
ENSG00000184007	PTP4A2	-1.7733847	0.01801069
ENSG00000137815	RTF1	-1.7721063	0.02407448
ENSG00000108175	ZMIZ1	-1.7699173	0.01301122
ENSG00000134058	CDK7	-1.7674828	0.01404513
ENSG00000033327	GAB2	-1.7656548	0.00427995
ENSG00000156535	CD109	-1.7612595	0.01948002
ENSG00000118181	RPS25	-1.7593344	0.04113773
ENSG00000116030	SUMO1	-1.7579458	0.00518293
ENSG00000104388	RAB2A	-1.7575095	0.01753258
ENSG00000260708	AL118516.1	-1.7563172	0.01641025
ENSG00000122482	ZNF644	-1.752177	0.01423498
ENSG00000185811	IKZF1	-1.7518942	0.00538725
ENSG00000198265	HELZ	-1.7505386	0.02861146
ENSG00000198355	PIM3	-1.748421	0.01386124
ENSG00000168092	PAFAH1B2	-1.7443667	0.01740339
ENSG00000112096	SOD2	-1.7439983	0.00366462
ENSG00000197780	TAF13	-1.7436453	0.03327495
ENSG00000142227	EMP3	-1.7416279	0.02066978
ENSG00000023287	RB1CC1	-1.7411566	0.03095398
ENSG00000168214	RBPJ	-1.7361356	0.02765943
ENSG00000162910	MRPL55	-1.7361119	0.04949211

ENSG00000168438	CDC40	-1.7360775	0.01602721
ENSG00000106052	TAX1BP1	-1.7324488	0.00349365
ENSG00000047249	ATP6V1H	-1.7225702	0.00694626
ENSG00000121749	TBC1D15	-1.7182926	0.01118014
ENSG00000132478	UNK	-1.7155076	0.04727882
ENSG00000147548	NSD3	-1.7152432	0.01757603
ENSG00000185947	ZNF267	-1.712521	0.04169608
ENSG00000131876	SNRPA1	-1.7094338	0.01750196
ENSG00000105993	DNAJB6	-1.7081557	0.00562571
ENSG00000051108	HERPUD1	-1.7078525	0.02402911
ENSG00000118620	ZNF430	-1.7032836	0.01359262
ENSG00000182568	SATB1	-1.701464	0.01892978
ENSG00000099622	CIRBP	-1.7006455	0.01599822
ENSG00000275740	AC091959.3	-1.7000485	0.02098066
ENSG00000117614	SYF2	-1.6995587	0.01404513
ENSG00000143761	ARF1	-1.6975774	0.03298113
ENSG00000109920	FNBP4	-1.6964028	0.01659642
ENSG00000153066	TXNDC11	-1.6916219	0.02356641
ENSG00000089737	DDX24	-1.6900113	0.00552569
ENSG00000164611	PTTG1	-1.6888559	0.01921875
ENSG00000150787	PTS	-1.6860297	0.00684755
ENSG00000128016	ZFP36	-1.6833588	0.01528261
ENSG00000255073	ZFP91-CNTF	-1.682648	0.02815656
ENSG00000198815	FOXJ3	-1.6814044	0.00471655
ENSG00000187109	NAP1L1	-1.6785751	0.02552889
ENSG00000116670	MAD2L2	-1.6785118	0.02775042
ENSG00000116560	SFPQ	-1.6754683	0.01636932
ENSG00000173960	UBXN2A	-1.6747454	0.00972062
ENSG00000100083	GGA1	-1.6745857	0.01040756
ENSG00000166848	TERF2IP	-1.6732968	0.03645327
ENSG00000188342	GTF2F2	-1.6714031	0.00427995
ENSG00000132326	PER2	-1.6705062	0.01473428
ENSG00000196470	SIAH1	-1.6700226	0.03185424
ENSG00000089234	BRAP	-1.6662239	0.02841211
ENSG00000034677	RNF19A	-1.6651371	0.04313599
ENSG00000167193	CRK	-1.6582165	0.03309042
ENSG00000132819	RBM38	-1.6578812	0.02822721
ENSG00000124214	STAU1	-1.6562073	0.03815966
ENSG00000162928	PEX13	-1.6557464	0.01698373
ENSG00000070495	JMJD6	-1.6520593	0.01492402

ENSG00000163682	RPL9	-1.6475759	0.02776518
ENSG00000234545	FAM133B	-1.6421073	0.01530022
ENSG00000136819	C9orf78	-1.6385135	0.0060948
ENSG00000217128	FNIP1	-1.6363172	0.02409658
ENSG00000106723	SPIN1	-1.6352289	0.04870387
ENSG00000143774	GUK1	-1.6344216	0.04337462
ENSG00000169641	LUZP1	-1.6338975	0.03185424
ENSG00000269968	AC006064.4	-1.6316226	0.01882969
ENSG00000122406	RPL5	-1.6300295	0.02259125
ENSG00000133226	SRRM1	-1.6299634	0.01882969
ENSG00000114125	RNF7	-1.6273242	0.03343504
ENSG00000151332	MBIP	-1.6225855	0.01681343
ENSG00000155508	CNOT8	-1.6224458	0.03766087
ENSG00000163374	YY1AP1	-1.6221834	0.02562787
ENSG00000138032	PPM1B	-1.613043	0.02578823
ENSG00000139572	GPR84	-1.6119569	0.04491806
ENSG00000091527	CDV3	-1.6111565	0.00715816
ENSG00000175348	TMEM9B	-1.6109897	0.01067079
ENSG00000106245	BUD31	-1.6072295	0.00366462
ENSG00000132334	PTPRE	-1.6041146	0.01101924
ENSG00000234745	HLA-B	-1.6021945	0.02302363
ENSG00000006634	DBF4	-1.6018116	0.0287894
ENSG00000179295	PTPN11	-1.6006623	0.01017501
ENSG00000156030	ELMSAN1	-1.5993026	0.00366462
ENSG00000061936	SFSWAP	-1.5980879	0.03455066
ENSG00000132388	UBE2G1	-1.5863168	0.00427995
ENSG00000100982	PCIF1	-1.586076	0.00458777
ENSG00000164032	H2AFZ	-1.5829662	0.02358364
ENSG00000117523	PRRC2C	-1.5823651	0.00421696
ENSG00000168615	ADAM9	-1.5815231	0.04551538
ENSG00000168137	SETD5	-1.5803551	0.04087958
ENSG00000116752	BCAS2	-1.5794338	0.04794577
ENSG00000033030	ZCCHC8	-1.5790862	0.04297077
ENSG00000163624	CDS1	-1.5761092	0.04870387
ENSG00000119048	UBE2B	-1.5728153	0.03917306
ENSG00000166747	AP1G1	-1.5719046	0.0383737
ENSG00000255112	CHMP1B	-1.5712458	0.04448535
ENSG00000143079	CTTNBP2NL	-1.5684593	0.01403954
ENSG00000146232	NFKBIE	-1.5668712	0.02052812
ENSG00000151461	UPF2	-1.5632883	0.0037268

ENSG00000180596	HIST1H2BC	-1.5602294	0.023638
ENSG00000054267	ARID4B	-1.5600476	0.0080564
ENSG00000179094	PER1	-1.5596991	0.03414375
ENSG00000147604	RPL7	-1.5523558	0.03067089
ENSG00000056097	ZFR	-1.5443294	0.02861146
ENSG00000090061	CCNK	-1.5435958	0.0108102
ENSG00000185043	CIB1	-1.5420194	0.00526983
ENSG00000177879	AP3S1	-1.5417923	0.00517546
ENSG00000170889	RPS9	-1.5371501	0.04672437
ENSG00000145414	NAF1	-1.5358676	0.00939014
ENSG00000134248	LAMTOR5	-1.535767	0.01905624
ENSG00000267165	CHMP1B-AS1	-1.53324	0.03839989
ENSG00000205937	RNPS1	-1.531859	0.01764812
ENSG00000137955	RABGGTB	-1.5311364	0.02187734
ENSG00000147526	TACC1	-1.5295004	0.00817443
ENSG00000271869	AC026979.3	-1.5276153	0.01698373
ENSG00000144848	ATG3	-1.5257477	0.02328206
ENSG00000198160	MIER1	-1.5249849	0.00783832
ENSG00000166266	CUL5	-1.5246382	0.02315457
ENSG00000138326	RPS24	-1.5235435	0.0495915
ENSG00000101166	PRELID3B	-1.5209207	0.01745368
ENSG00000187514	PTMA	-1.5196051	0.00812086
ENSG00000102409	BEX4	-1.5147338	0.00366462
ENSG00000204178	TMEM57	-1.5111317	0.04977237
ENSG00000184014	DENND5A	-1.5096529	0.0311748
ENSG00000109220	CHIC2	-1.5092584	0.02274656
ENSG00000143256	PFDN2	-1.5092422	0.02230772
ENSG00000168036	CTNNB1	-1.5081516	0.0488667
ENSG00000259884	AC025259.3	-1.5079142	0.0291451
ENSG00000156735	BAG4	-1.507731	0.03297477
ENSG00000119844	AFTPH	-1.5075552	0.0383737
ENSG00000137185	ZSCAN9	-1.5056565	0.04795835
ENSG00000184922	FMNL1	-1.5042614	0.00427995
ENSG00000203644	AC083799.1	-1.5031031	0.00604041
ENSG00000129484	PARP2	-1.5019141	0.01498374
ENSG00000172062	SMN1	-1.5017795	0.00809412
ENSG00000114354	TFG	-1.5006429	0.02308955
ENSG00000090905	TNRC6A	-1.5001209	0.01786554

DEG Mo pSS versus HD

Transcipt ID	Gene name	Log2(FC)	FDR < 0.05
ENSG00000088827	SIGLEC1	4.81222715	0.00061969
ENSG00000127951	FGL2	4.63307294	3.69E-05
ENSG00000177409	SAMD9L	4.48867916	7.2716E-05
ENSG00000111331	OAS3	4.42230211	0.00037088
ENSG00000111335	OAS2	4.41241143	0.00023971
ENSG00000182578	CSF1R	4.12149732	0.00018821
ENSG00000111913	RIPOR2	4.05133531	5.6748E-05
ENSG00000204131	NHSL2	3.98423953	8.698E-05
ENSG00000089127	OAS1	3.88177349	0.00014848
ENSG00000082074	FYB1	3.86940796	0.00011131
ENSG00000168329	CX3CR1	3.66901016	0.00346942
ENSG00000132530	XAF1	3.57786021	0.00019855
ENSG00000137959	IFI44L	3.56620595	0.00405956
ENSG00000106780	MEGF9	3.50812058	0.00020506
ENSG00000110876	SELPLG	3.45149629	3.725E-05
ENSG00000119917	IFIT3	3.41478814	0.00578098
ENSG00000157601	MX1	3.40051191	0.00273973
ENSG00000093072	ADA2	3.30929409	0.00035785
ENSG00000137628	DDX60	3.26174787	0.00017728
ENSG00000119922	IFIT2	3.15400711	0.00698448
ENSG00000096968	JAK2	3.1171167	4.002E-05
ENSG00000183486	MX2	3.0908071	0.00012058
ENSG00000203747	FCGR3A	3.05824159	0.00908083
ENSG00000185745	IFIT1	3.04472512	0.00769807
ENSG00000092964	DPYSL2	3.03157003	0.00036453
ENSG00000179583	CIITA	2.97329048	3.69E-05
ENSG00000178927	C17orf62	2.95098495	0.00011008
ENSG00000146192	FGD2	2.94266455	3.69E-05
ENSG00000171115	GIMAP8	2.91127768	0.00035769
ENSG00000121858	TNFSF10	2.90815474	0.00085748
ENSG00000101347	SAMHD1	2.8875798	0.00020568
ENSG00000107551	RASSF4	2.86741339	0.00020286
ENSG00000133706	LARS	2.85358246	0.00024204
ENSG00000157637	SLC38A10	2.83559968	4.0167E-05
ENSG00000105483	CARD8	2.82180872	0.00010905
ENSG00000134321	RSAD2	2.8216868	0.00044978
ENSG00000166801	FAM111A	2.82114926	4.0167E-05

ENSG00000133574	GIMAP4	2.80831292	0.00205538
ENSG00000005020	SKAP2	2.80342257	0.00011131
ENSG00000107201	DDX58	2.79847046	7.6564E-05
ENSG00000139970	RTN1	2.79625262	0.00161234
ENSG00000143624	INTS3	2.76935299	0.00025431
ENSG00000135218	CD36	2.7687755	0.00441468
ENSG00000121807	CCR2	2.76812231	0.00459916
ENSG00000134326	CMPK2	2.76441331	7.007E-05
ENSG00000124491	F13A1	2.76137384	0.0436662
ENSG00000038427	VCAN	2.76089778	0.00031344
ENSG00000139278	GLIPR1	2.75148728	3.69E-05
ENSG00000188641	DPYD	2.7460871	0.00016589
ENSG00000160593	JAML	2.73785141	1.5464E-05
ENSG00000129003	VPS13C	2.72450583	0.00042793
ENSG00000177575	CD163	2.7119008	0.00130504
ENSG00000101336	HCK	2.69647494	4.6204E-05
ENSG00000196975	ANXA4	2.68121227	0.00013137
ENSG00000143546	S100A8	2.67257265	0.00020529
ENSG00000133835	HSD17B4	2.66258299	5.2951E-05
ENSG00000103381	CPPED1	2.65717733	0.00031447
ENSG00000181381	DDX60L	2.6567232	0.00050571
ENSG00000105953	OGDH	2.64511854	0.00021448
ENSG00000130449	ZSWIM6	2.63544798	7.2413E-05
ENSG00000187608	ISG15	2.62966453	0.00010145
ENSG00000165168	CYBB	2.62649243	0.00017515
ENSG00000163683	SMIM14	2.584365	0.00013629
ENSG00000215458	AATBC	2.57203412	5.6247E-05
ENSG00000196209	SIRPB2	2.55920596	0.00116963
ENSG00000073849	ST6GAL1	2.550513	0.00048075
ENSG00000249437	NAIP	2.54772158	0.03856131
ENSG00000138119	MYOF	2.52298352	0.00116629
ENSG00000133106	EPSTI1	2.51730507	0.00024243
ENSG00000163563	MNDA	2.51154611	0.00019527
ENSG00000240065	PSMB9	2.49985703	0.00043175
ENSG00000134256	CD101	2.49461745	0.00672014
ENSG00000185722	ANKFY1	2.4915029	0.00017719
ENSG00000006756	ARSD	2.456905	0.00034289
ENSG00000068079	IFI35	2.45084083	0.00011131
ENSG00000102524	TNFSF13B	2.4295903	0.00016701
ENSG00000110665	C11orf21	2.42600156	4.3201E-05
ENSG00000142405	NLRP12	2.42233631	0.0011604
ENSG00000213203	GIMAP1	2.40060946	0.00029261

ENSG00000250138	AC139495.3	2.39948877	0.00070469
ENSG00000127954	STEAP4	2.39860441	0.02252014
ENSG00000138246	DNAJC13	2.38403223	0.00029419
ENSG00000178685	PARP10	2.38363732	1.8312E-05
ENSG00000128872	TMOD2	2.38333014	0.00011131
ENSG00000163565	IFI16	2.37871911	5.9604E-05
ENSG00000165092	ALDH1A1	2.36838002	0.00200702
ENSG00000149131	SERPING1	2.36410268	0.0017831
ENSG00000100364	KIAA0930	2.3531638	7.9831E-05
ENSG00000135636	DYSF	2.35302925	0.00056476
ENSG00000126709	IFI6	2.35060464	0.00045242
ENSG00000111961	SASH1	2.34814151	0.04833761
ENSG00000050344	NFE2L3	2.33003379	5.9604E-05
ENSG00000142089	IFITM3	2.32819656	0.00076829
ENSG00000146592	CREB5	2.32706132	0.00069182
ENSG00000119638	NEK9	2.32309385	8.2108E-05
ENSG00000140575	IQGAP1	2.30892835	0.00026643
ENSG00000105967	TFEC	2.30676328	8.2108E-05
ENSG00000081189	MEF2C	2.29629681	0.00037032
ENSG00000142687	KIAA0319L	2.28793993	0.0002003
ENSG00000137965	IFI44	2.27380106	0.00211031
ENSG00000138413	IDH1	2.27237293	0.00056064
ENSG00000168310	IRF2	2.27032454	0.00101781
ENSG00000229754	CXCR2P1	2.26268149	0.0008627
ENSG00000106785	TRIM14	2.26268053	0.00011864
ENSG00000135709	KIAA0513	2.26070245	0.00011131
ENSG00000140749	IGSF6	2.25914668	0.00110115
ENSG00000188554	NBR1	2.24022924	0.00011131
ENSG00000058668	ATP2B4	2.23782734	0.00196697
ENSG00000165071	TMEM71	2.22947868	0.00013137
ENSG00000175567	UCP2	2.2209786	0.00179198
ENSG00000031081	ARHGAP31	2.21924073	0.00241362
ENSG00000133943	DGLUCY	2.21857708	4.0167E-05
ENSG00000187554	TLR5	2.21767842	0.00029084
ENSG00000188404	SELL	2.21406286	0.00152087
ENSG00000102189	EEA1	2.21309813	0.00046523
ENSG00000180357	ZNF609	2.21122874	6.6556E-05
ENSG00000138646	HERC5	2.21122579	0.00014889
ENSG00000103313	MEFV	2.20838651	0.00024218
ENSG00000139687	RB1	2.20778929	9.703E-05
ENSG00000115232	ITGA4	2.1977504	0.00066046
ENSG00000081087	OSTM1	2.18697561	5.2951E-05

ENSG00000115155	OTOF	2.17916828	0.04578118
ENSG00000110077	MS4A6A	2.17144347	0.00035769
ENSG00000173821	RNF213	2.16238113	0.00043225
ENSG00000198734	F5	2.15850947	0.00025416
ENSG00000198951	NAGA	2.1583494	0.00023076
ENSG00000150867	PIP4K2A	2.15794943	0.00017634
ENSG00000071967	CYBRD1	2.15624005	0.00158569
ENSG00000074706	IPCEF1	2.15391304	0.00079352
ENSG00000166326	TRIM44	2.15103466	0.00011131
ENSG00000121210	TMEM131L	2.15055037	0.00080531
ENSG00000108771	DHX58	2.12712832	0.00088319
ENSG00000112367	FIG4	2.11750045	2.6941E-05
ENSG00000165672	PRDX3	2.11413223	0.00011131
ENSG00000138756	BMP2K	2.10876117	0.00014473
ENSG00000183023	SLC8A1	2.10770505	0.00140107
ENSG00000161929	SCIMP	2.10518218	0.00054607
ENSG00000082996	RNF13	2.1003053	0.00042489
ENSG00000107099	DOCK8	2.09599002	0.00042323
ENSG00000025708	TYMP	2.09573646	0.00353048
ENSG00000160551	TAOK1	2.0948519	0.00031447
ENSG00000163946	FAM208A	2.08670497	0.00025562
ENSG00000179144	GIMAP7	2.08182365	0.01499835
ENSG00000138496	PARP9	2.07771882	0.00058507
ENSG00000135838	NPL	2.07449096	0.00096657
ENSG00000173083	HPSE	2.06771702	0.00112582
ENSG00000119321	FKBP15	2.06242754	0.00027781
ENSG00000166927	MS4A7	2.06134147	0.00363434
ENSG00000143970	ASXL2	2.05713821	0.0002004
ENSG00000124942	AHNAK	2.05330381	0.00037122
ENSG00000145246	ATP10D	2.04835108	0.00039682
ENSG00000149311	ATM	2.04810493	3.69E-05
ENSG00000165476	REEP3	2.04794403	0.0002967
ENSG00000133561	GIMAP6	2.04177072	0.00071202
ENSG00000163220	S100A9	2.03585241	0.00044987
ENSG00000084733	RAB10	2.03363615	0.0001934
ENSG00000197043	ANXA6	2.02891787	0.0074927
ENSG00000100342	APOL1	2.02806304	0.00710375
ENSG00000088986	DYNLL1	2.02678083	3.69E-05
ENSG00000180370	PAK2	2.02592389	0.00021444
ENSG00000073969	NSF	2.02382863	0.00021098
ENSG00000133313	CNDP2	2.02318759	0.00070469
ENSG00000129566	TEP1	2.02316324	0.00094714

ENSG00000170006	TMEM154	2.02228185	0.00033278
ENSG00000128815	WDFY4	2.01621146	0.0007939
ENSG00000280153	AC133065.6	2.01591236	0.00098903
ENSG00000130589	HELZ2	2.01531518	0.00059211
ENSG00000203710	CR1	2.00968798	0.00167253
ENSG00000111269	CREBL2	1.99637291	0.0004298
ENSG00000160216	AGPAT3	1.9931901	0.00080171
ENSG00000188906	LRRK2	1.99158214	0.00021321
ENSG00000168010	ATG16L2	1.99121182	0.00011877
ENSG00000040933	INPP4A	1.99048334	8.82E-05
ENSG00000186088	GSAP	1.98529176	0.00022632
ENSG00000123213	NLN	1.98353142	0.01069252
ENSG00000122986	HVCN1	1.98183554	0.00017899
ENSG00000135457	TFCP2	1.97973391	5.9604E-05
ENSG00000136485	DCAF7	1.97954393	0.0003913
ENSG00000065413	ANKRD44	1.97584367	3.4895E-05
ENSG00000131374	TBC1D5	1.97247733	0.00033575
ENSG00000084070	SMAP2	1.97215259	0.00518494
ENSG00000155957	TMBIM4	1.97188602	4.9812E-05
ENSG00000169220	RGS14	1.9697004	5.9265E-05
ENSG00000104133	SPG11	1.96773469	8.698E-05
ENSG00000116237	ICMT	1.96760299	5.4121E-05
ENSG00000145416	MARCH1	1.96445135	0.00108021
ENSG00000198585	NUDT16	1.96325971	0.00025638
ENSG00000153936	HS2ST1	1.96273796	5.4121E-05
ENSG00000137752	CASP1	1.95801983	0.00041379
ENSG00000133216	EPHB2	1.94999142	0.00037151
ENSG00000158517	NCF1	1.9496836	0.00084495
ENSG00000197548	ATG7	1.94397589	0.00018547
ENSG00000121281	ADCY7	1.93336445	0.00041634
ENSG00000170581	STAT2	1.93316979	0.00175064
ENSG00000151466	SCLT1	1.93308209	0.00075271
ENSG00000145287	PLAC8	1.93174465	0.00184434
ENSG00000166888	STAT6	1.93017847	0.00051901
ENSG00000136040	PLXNC1	1.92853493	0.00025416
ENSG00000156587	UBE2L6	1.92681789	0.00130163
ENSG00000122694	GLIPR2	1.92658528	0.00020061
ENSG00000226137	BAIAP2-AS1	1.92619115	0.00170844
ENSG00000136631	VPS45	1.92511228	0.00010269
ENSG00000166272	WBP1L	1.92179463	0.00031743
ENSG00000107929	LARP4B	1.91918736	0.00013762
ENSG00000090863	GLG1	1.90303374	3.69E-05

ENSG00000010810	FYN	1.90139286	0.00012627
ENSG00000072501	SMC1A	1.90091004	0.00024409
ENSG00000093144	ECHDC1	1.90087661	0.00020061
ENSG00000155660	PDIA4	1.89991655	5.8839E-05
ENSG00000150681	RGS18	1.89711599	0.00022322
ENSG00000104974	LILRA1	1.89627793	0.00225028
ENSG00000198771	RCSD1	1.89415863	0.00013674
ENSG00000105501	SIGLEC5	1.89344721	0.0002242
ENSG00000103479	RBL2	1.88225062	0.00011163
ENSG00000091106	NLRC4	1.88025166	0.00037654
ENSG00000103335	PIEZO1	1.87511955	0.00136249
ENSG00000010292	NCAPD2	1.869991	0.00013089
ENSG00000008130	NADK	1.86881805	0.000305
ENSG00000162946	DISC1	1.86805719	0.00253017
ENSG00000138640	FAM13A	1.86643031	0.00278912
ENSG00000140090	SLC24A4	1.86540488	8.2108E-05
ENSG00000129675	ARHGEF6	1.85487557	0.00021321
ENSG00000157827	FMNL2	1.85417167	0.00017617
ENSG00000110031	LPXN	1.84675535	0.00058189
ENSG00000112419	PHACTR2	1.83062034	0.0002053
ENSG00000137478	FCHSD2	1.82957906	0.00059545
ENSG00000020577	SAMD4A	1.82723663	6.8112E-05
ENSG00000166197	NOLC1	1.82686298	0.00019633
ENSG00000181192	DHTKD1	1.82658412	0.00095394
ENSG00000164054	SHISA5	1.82575246	0.00014848
ENSG00000171853	TRAPPC12	1.82407323	0.00038272
ENSG00000134996	OSTF1	1.82067029	0.0001277
ENSG00000132182	NUP210	1.81876085	0.0006356
ENSG00000184979	USP18	1.81821607	0.00018668
ENSG00000154930	ACSS1	1.81709923	0.00043615
ENSG00000167220	HDHD2	1.81576718	0.0001277
ENSG00000159339	PADI4	1.8129343	0.00127516
ENSG00000151702	FLI1	1.81161047	0.00062992
ENSG00000250510	GPR162	1.81124772	0.00281025
ENSG00000166928	MS4A14	1.80676496	0.00387978
ENSG00000213648	SULT1A4	1.80594935	0.00220992
ENSG00000177853	ZNF518A	1.80539933	0.00031839
ENSG00000145703	IQGAP2	1.80357672	0.00900436
ENSG00000120594	PLXDC2	1.79914964	0.00207841
ENSG00000121691	CAT	1.79908906	0.00030322
ENSG00000132256	TRIM5	1.79834867	0.00041435
ENSG00000064763	FAR2	1.79614448	0.0001848

ENSG00000197629	MPEG1	1.79410737	0.00348206
ENSG00000108389	MTMR4	1.79292546	4.0167E-05
ENSG00000258659	TRIM34	1.78714573	1.5464E-05
ENSG00000101346	POFUT1	1.78666583	0.00019592
ENSG00000263528	IKBKE	1.78215843	0.00013089
ENSG00000133816	MICAL2	1.77999999	0.00965276
ENSG00000105281	SLC1A5	1.77845426	0.00096657
ENSG00000138449	SLC40A1	1.77795244	0.00044978
ENSG00000185885	IFITM1	1.77549121	0.00195944
ENSG00000164808	SPIDR	1.77402505	4.6204E-05
ENSG00000164414	SLC35A1	1.76140878	6.077E-05
ENSG00000214078	CPNE1	1.75638232	0.0061442
ENSG00000155097	ATP6V1C1	1.75552976	6.8996E-05
ENSG00000130150	MOSPD2	1.75525532	0.02616316
ENSG00000164062	APEH	1.75516083	0.00042376
ENSG00000129993	CBFA2T3	1.75416807	0.00034175
ENSG00000196664	TLR7	1.75117674	0.03127928
ENSG00000196730	DAPK1	1.74965437	0.00305556
ENSG00000145715	RASA1	1.74920879	6.4871E-05
ENSG00000250264	AL669918.1	1.74666005	0.04869143
ENSG00000001629	ANKIB1	1.7450679	4.0167E-05
ENSG00000143669	LYST	1.74377161	0.0001798
ENSG00000177119	ANO6	1.73797693	4.0167E-05
ENSG00000113845	TIMMDC1	1.73688181	0.00023416
ENSG00000108798	ABI3	1.73475905	0.0010977
ENSG00000076770	MBNL3	1.73193071	7.5228E-05
ENSG00000134955	SLC37A2	1.72948671	0.00224493
ENSG00000112079	STK38	1.72691606	0.00026307
ENSG00000168995	SIGLEC7	1.72316115	0.00234137
ENSG00000174600	CMKLR1	1.72163844	0.00172775
ENSG00000142784	WDTC1	1.72084308	4.0167E-05
ENSG00000118596	SLC16A7	1.71739468	0.00224493
ENSG00000159228	CBR1	1.71678557	0.00016348
ENSG00000164308	ERAP2	1.71653322	0.03650502
ENSG00000117115	PADI2	1.71315931	0.00446116
ENSG00000103196	CRISPLD2	1.71032628	0.00540432
ENSG00000198814	GK	1.708838	0.0100024
ENSG00000005844	ITGAL	1.70855868	0.00248508
ENSG00000136161	RCBTB2	1.70732997	0.00050507
ENSG00000165675	ENOX2	1.70371623	0.00034874
ENSG00000254838	GVINP1	1.70358337	0.00371368
ENSG00000154822	PLCL2	1.70345847	0.00242728

ENSG00000280071	FP565260.6	1.6991183	0.0033487
ENSG00000138459	SLC35A5	1.69403765	0.00018322
ENSG00000198736	MSRB1	1.68859047	0.00042489
ENSG00000114770	ABCC5	1.68856034	0.00092437
ENSG00000135842	FAM129A	1.68719891	0.00173207
ENSG00000178695	KCTD12	1.68715468	0.00048075
ENSG00000103544	C16orf62	1.68559026	0.00013674
ENSG00000182511	FES	1.68541817	0.0003573
ENSG00000165819	METTL3	1.67918653	0.00013429
ENSG00000143110	C1orf162	1.67827229	0.00036405
ENSG00000136816	TOR1B	1.67818427	0.00021444
ENSG00000184992	BRI3BP	1.6780333	0.0004298
ENSG00000175538	KCNE3	1.67614598	4.3201E-05
ENSG00000101695	RNF125	1.67437375	0.00086119
ENSG00000088888	MAVS	1.67434021	0.00090564
ENSG00000124357	NAGK	1.67011388	0.00078899
ENSG00000077420	APBB1IP	1.66646142	0.00011229
ENSG00000006715	VPS41	1.6644439	0.00014019
ENSG00000089597	GANAB	1.66262829	0.00017634
ENSG00000138642	HERC6	1.65949223	0.00015833
ENSG00000101307	SIRPB1	1.65722086	0.01664438
ENSG00000104365	IKBKB	1.65533587	0.00016701
ENSG00000175216	CKAP5	1.65500781	0.00116057
ENSG00000166002	SMCO4	1.65376135	0.00130388
ENSG00000119457	SLC46A2	1.65167071	0.0023381
ENSG00000135899	SP110	1.65130811	0.00013089
ENSG00000090861	AARS	1.64939027	0.00110912
ENSG00000026652	AGPAT4	1.64716441	0.0008941
ENSG00000204261	PSMB8-AS1	1.64527738	0.00138063
ENSG00000103047	TANGO6	1.64454838	0.00187327
ENSG00000196782	MAML3	1.64105639	0.00074128
ENSG00000142185	TRPM2	1.64064032	0.00191016
ENSG00000172164	SNTB1	1.63923717	0.0017562
ENSG00000087274	ADD1	1.63577739	0.00053834
ENSG00000110934	BIN2	1.63492943	0.0018027
ENSG00000075303	SLC25A40	1.63413334	0.0001277
ENSG00000175309	PHYKPL	1.63396822	0.00275355
ENSG00000177628	GBA	1.63228568	0.00179412
ENSG00000198133	TMEM229B	1.6321927	0.01145874
ENSG00000175471	MCTP1	1.63149672	0.00014889
ENSG00000167085	PHB	1.6309904	0.00072824
ENSG00000168016	TRANK1	1.63095088	0.00080171

ENSG00000119397	CNTRL	1.63015137	0.00400993
ENSG00000162704	ARPC5	1.62932633	0.00023416
ENSG00000020129	NCDN	1.62621196	0.00081608
ENSG00000146776	ATXN7L1	1.62399274	0.00026334
ENSG00000197142	ACSL5	1.62105862	0.00051346
ENSG00000214013	GANC	1.62065399	0.00014848
ENSG00000115415	STAT1	1.62023511	0.00431728
ENSG00000099326	MZF1	1.61914023	0.00012317
ENSG00000176783	RUFY1	1.61894132	0.00059298
ENSG00000163328	GPR155	1.61543313	0.00032578
ENSG00000108468	CBX1	1.61471009	0.00010905
ENSG00000205133	TRIQK	1.61434355	0.00085331
ENSG00000004455	AK2	1.61380087	0.00025545
ENSG00000152818	UTRN	1.61190641	0.00268679
ENSG00000130021	PUDP	1.6114027	3.69E-05
ENSG00000113522	RAD50	1.60816637	0.00048893
ENSG00000148110	MFSD14B	1.60660809	0.00324543
ENSG00000179921	GPBAR1	1.6060237	0.00011774
ENSG00000101337	TM9SF4	1.60519099	0.00011131
ENSG00000185482	STAC3	1.60443502	0.00015833
ENSG00000130303	BST2	1.60174167	0.00075271
ENSG00000112297	CRYBG1	1.59901085	0.00026047
ENSG00000086300	SNX10	1.5940843	7.5228E-05
ENSG00000179978	AC140134.1	1.59225393	0.01034033
ENSG00000134452	FBXO18	1.58838748	0.00030322
ENSG00000162869	PPP1R21	1.58588347	0.00048075
ENSG00000109436	TBC1D9	1.58441502	0.00019192
ENSG00000101974	ATP11C	1.5839431	8.4055E-05
ENSG00000186111	PIP5K1C	1.5818681	0.00026203
ENSG00000161955	TNFSF13	1.58119351	0.00020436
ENSG00000158467	AHCYL2	1.57606659	0.00011131
ENSG00000186063	AIDA	1.57409347	0.00046529
ENSG00000178537	SLC25A20	1.57353802	0.00081597
ENSG00000111667	USP5	1.57295741	0.00031694
ENSG00000131844	MCCC2	1.57282243	0.0002418
ENSG00000143119	CD53	1.57109935	0.00021484
ENSG00000160211	G6PD	1.56978257	0.000601
ENSG00000143382	ADAMTSL4	1.56948514	0.00264256
ENSG00000070814	TCOF1	1.56868052	0.00047455
ENSG00000173193	PARP14	1.56740882	0.00445351
ENSG00000180353	HCLS1	1.56664584	0.00021343
ENSG00000115904	SOS1	1.56418694	0.0009446

ENSG00000049239	H6PD	1.56086241	0.0023701
ENSG00000166145	SPINT1	1.56056375	0.00019592
ENSG00000175662	TOM1L2	1.56043927	0.00019192
ENSG00000108679	LGALS3BP	1.56029735	0.01089084
ENSG00000107736	CDH23	1.5591635	0.00195481
ENSG00000058866	DGKG	1.55747451	0.00087231
ENSG00000138814	PPP3CA	1.55720597	0.00079219
ENSG00000134851	TMEM165	1.55709643	3.69E-05
ENSG00000137509	PRCP	1.55445473	5.2936E-05
ENSG00000164023	SGMS2	1.54486665	0.00023497
ENSG00000070190	DAPP1	1.54042513	0.00052724
ENSG00000136305	CIDEB	1.53982943	0.00035831
ENSG00000162736	NCSTN	1.53913214	0.00014247
ENSG00000160883	HK3	1.53712863	0.00139367
ENSG00000146070	PLA2G7	1.53676805	0.0003987
ENSG00000100030	MAPK1	1.53661952	0.00038097
ENSG00000143933	CALM2	1.53556489	0.00061541
ENSG00000173786	CNP	1.53534972	0.00189816
ENSG00000117228	GBP1	1.53441652	0.02972753
ENSG00000172269	DPAGT1	1.53404302	0.00017204
ENSG00000151151	IPMK	1.53347652	0.01739805
ENSG00000077380	DYNC1I2	1.53322432	0.00032015
ENSG00000100201	DDX17	1.53199949	0.00146578
ENSG00000164068	RNF123	1.52989919	0.01662566
ENSG00000130429	ARPC1B	1.52804571	0.00173207
ENSG00000135905	DOCK10	1.52795493	0.00066095
ENSG00000138639	ARHGAP24	1.52793953	0.00030893
ENSG00000111540	RAB5B	1.52701183	0.00026203
ENSG00000163840	DTX3L	1.52401629	0.00057399
ENSG00000164125	FAM198B	1.51862512	0.00842223
ENSG00000238227	TMEM250	1.51500264	0.00056375
ENSG00000196684	HSH2D	1.51431852	5.6748E-05
ENSG00000175857	GAPT	1.5109292	0.00103798
ENSG00000160310	PRMT2	1.51058035	0.00063722
ENSG00000148484	RSU1	1.50881568	0.00104815
ENSG00000131018	SYNE1	1.50740295	0.00289354
ENSG00000163154	TNFAIP8L2	1.5067246	0.00059557
ENSG00000007923	DNAJC11	1.50596034	0.00047624
ENSG00000147471	PLPBP	1.50432034	8.2108E-05
ENSG00000146376	ARHGAP18	1.5025789	0.00011131
ENSG00000067704	IARS2	1.50016081	0.0001848
ENSG00000169429	CXCL8	-5.6337326	0.00464143

ENSG00000123689	G0S2	-5.3324527	0.01637209
ENSG00000137801	THBS1	-5.2549864	0.01454249
ENSG00000148344	PTGES	-4.7753901	0.00509201
ENSG00000162496	DHRS3	-4.498196	0.00892293
ENSG00000112137	PHACTR1	-4.4357644	0.02363157
ENSG00000184205	TSPYL2	-4.2134831	0.00390017
ENSG00000081041	CXCL2	-4.1267357	0.01287257
ENSG00000102760	RGCC	-4.0251224	0.020498
ENSG00000176597	B3GNT5	-3.9922784	0.00753884
ENSG00000118971	CCND2	-3.9763733	0.02016236
ENSG00000178726	THBD	-3.9694314	0.03090869
ENSG00000139112	GABARAPL1	-3.9174867	0.00344943
ENSG00000163376	KBTBD8	-3.7481036	0.01002672
ENSG00000160789	LMNA	-3.59249	0.03524042
ENSG00000100644	HIF1A	-3.5458608	0.00057401
ENSG00000153207	AHCTF1	-3.5254676	0.0084494
ENSG00000008083	JARID2	-3.3847124	0.00115696
ENSG00000182782	HCAR2	-3.3290246	0.02444392
ENSG00000188042	ARL4C	-3.3023161	0.03434752
ENSG00000125740	FOSB	-3.2798454	0.01611739
ENSG00000126351	THRA	-3.2650285	0.00891857
ENSG00000124145	SDC4	-3.261413	0.00214841
ENSG00000118985	ELL2	-3.2453856	0.03585554
ENSG00000113369	ARRDC3	-3.2098649	0.0063002
ENSG00000171604	CXXC5	-3.2020789	0.0162197
ENSG00000113448	PDE4D	-3.1957399	0.02085715
ENSG00000169508	GPR183	-3.1681931	0.00722174
ENSG00000277117	FP565260.3	-3.1326512	0.00510494
ENSG00000166920	C15orf48	-3.1238849	0.01391301
ENSG00000153234	NR4A2	-3.1148471	0.00604553
ENSG00000078804	TP53INP2	-3.1113741	0.00575271
ENSG00000168036	CTNNB1	-3.1044079	0.00037088
ENSG00000130340	SNX9	-3.0836264	0.00821045
ENSG00000110046	ATG2A	-3.082018	0.00672133
ENSG00000255398	HCAR3	-3.0667303	0.00655629
ENSG00000088826	SMOX	-3.0513615	0.00168846
ENSG00000008056	SYN1	-3.017608	0.00202534
ENSG00000151014	NOCT	-3.0104672	0.0001848
ENSG00000196878	LAMB3	-2.9950369	0.03525671
ENSG00000120063	GNA13	-2.9950306	0.00263842
ENSG00000165029	ABCA1	-2.9948132	0.00923629
ENSG00000231721	LINC-PINT	-2.9796939	0.00123023

ENSG00000112715	VEGFA	-2.9746894	0.00734878
ENSG00000173575	CHD2	-2.9705471	0.0001277
ENSG00000113070	HBEGF	-2.9627795	0.00106234
ENSG00000169155	ZBTB43	-2.9596239	0.00175002
ENSG00000137507	LRRC32	-2.9517331	0.00049759
ENSG00000106089	STX1A	-2.9492251	0.01038173
ENSG00000104312	RIPK2	-2.9480134	5.2951E-05
ENSG00000075426	FOSL2	-2.9416515	0.0194375
ENSG00000171174	RBKS	-2.9359671	0.0252909
ENSG00000271614	ATP2B1-AS1	-2.9328893	0.0007034
ENSG00000173281	PPP1R3B	-2.9287612	0.03212843
ENSG00000185477	GPRIN3	-2.9252603	0.0011287
ENSG00000185022	MAFF	-2.9154679	0.03122127
ENSG00000128271	ADORA2A	-2.9125523	0.00079208
ENSG00000155252	PI4K2A	-2.9113472	0.00361432
ENSG00000070961	ATP2B1	-2.8902578	0.03972003
ENSG00000113742	CPEB4	-2.8816276	0.01257684
ENSG00000160223	ICOSLG	-2.8795385	0.00645006
ENSG00000184588	PDE4B	-2.8711138	0.00128813
ENSG00000244242	IFITM10	-2.870418	0.00188837
ENSG00000180530	NRIP1	-2.8643287	0.00170163
ENSG00000166949	SMAD3	-2.8640214	0.00335679
ENSG00000134070	IRAK2	-2.8624185	0.01250651
ENSG00000099985	OSM	-2.8597775	0.00029425
ENSG00000144476	ACKR3	-2.8573954	0.00741895
ENSG00000086062	B4GALT1	-2.8457697	0.00096056
ENSG00000160271	RALGDS	-2.8352271	0.00840216
ENSG00000161921	CXCL16	-2.8324701	0.00334081
ENSG00000108828	VAT1	-2.8268836	0.00453696
ENSG00000134531	EMP1	-2.7956852	0.0093593
ENSG00000112149	CD83	-2.7844651	0.00630094
ENSG00000132819	RBM38	-2.7792212	0.00700209
ENSG00000108179	PPIF	-2.7523932	0.01740293
ENSG00000133657	ATP13A3	-2.7405907	0.00591523
ENSG00000128342	LIF	-2.725365	0.02156661
ENSG00000183696	UPP1	-2.7206894	3.816E-06
ENSG00000011422	PLAUR	-2.7006523	0.01328449
ENSG00000198742	SMURF1	-2.6925219	0.00011131
ENSG00000115548	KDM3A	-2.6723105	0.00034172
ENSG00000122644	ARL4A	-2.667149	0.00045749
ENSG00000116604	MEF2D	-2.6511897	0.01193626
ENSG00000143622	RIT1	-2.6418557	0.01017276

ENSG00000133639	BTG1	-2.6096684	0.00691552
ENSG00000057657	PRDM1	-2.5991526	0.00025626
ENSG00000105835	NAMPT	-2.5885866	0.01033927
ENSG00000161835	GRASP	-2.5845808	0.0444488
ENSG00000139572	GPR84	-2.5804233	0.03542708
ENSG00000060558	GNA15	-2.5766184	0.014623
ENSG00000059804	SLC2A3	-2.5444028	0.00359548
ENSG00000140332	TLE3	-2.5403499	0.00424621
ENSG00000137309	HMGA1	-2.5322661	0.00031626
ENSG00000234290	AC116366.1	-2.5197718	0.03965652
ENSG00000055483	USP36	-2.5173927	0.00376868
ENSG00000095794	CREM	-2.5126091	0.02652157
ENSG00000162711	NLRP3	-2.5091521	0.00029457
ENSG00000025156	HSF2	-2.4950352	0.00038666
ENSG00000170525	PFKFB3	-2.4903444	4.0167E-05
ENSG00000132952	USPL1	-2.4856584	0.00113974
ENSG00000189067	LITAF	-2.4772791	0.02578617
ENSG00000153094	BCL2L11	-2.4749415	0.00351515
ENSG00000162889	MAPKAPK2	-2.4565655	0.00581231
ENSG00000130202	NECTIN2	-2.4542862	0.03099256
ENSG00000153922	CHD1	-2.4384453	0.000185
ENSG00000162772	ATF3	-2.4341496	0.00106477
ENSG00000137409	MTCH1	-2.4266198	0.00096657
ENSG00000004660	CAMKK1	-2.4208654	0.00010145
ENSG00000211445	GPX3	-2.4189819	0.02016236
ENSG00000239305	RNF103	-2.4170593	1.2095E-05
ENSG00000121966	CXCR4	-2.4156745	0.00711902
ENSG00000141503	MINK1	-2.4153969	0.00443835
ENSG00000177426	TGIF1	-2.4146208	0.00170844
ENSG00000085117	CD82	-2.4142602	0.00082171
ENSG00000120705	ETF1	-2.4137603	0.00115407
ENSG00000143862	ARL8A	-2.4017085	0.00690308
ENSG00000154640	BTG3	-2.3777685	0.00037088
ENSG00000110721	CHKA	-2.3690688	0.00280401
ENSG00000175352	NRIP3	-2.3658043	0.00702715
ENSG00000104419	NDRG1	-2.3649559	0.00786721
ENSG00000166579	NDEL1	-2.3578224	0.00828844
ENSG00000105851	PIK3CG	-2.3529044	0.00109512
ENSG00000144597	EAF1	-2.3476008	0.00638678
ENSG00000158470	B4GALT5	-2.3460849	0.00018367
ENSG00000153201	RANBP2	-2.3460562	0.00176715
ENSG00000116574	RHOU	-2.3456583	0.00530599

ENSG00000143153	ATP1B1	-2.3436801	0.00077849
ENSG00000136603	SKIL	-2.3399632	0.01964359
ENSG00000112511	PHF1	-2.3395331	0.00017634
ENSG00000177374	HIC1	-2.3365351	0.04264038
ENSG00000130024	PHF10	-2.3349711	0.00376857
ENSG00000179361	ARID3B	-2.3322307	0.00836551
ENSG00000143851	PTPN7	-2.3311657	0.00019919
ENSG00000272888	LINC01578	-2.3298954	0.00039024
ENSG00000146457	WTAP	-2.329158	0.00024688
ENSG00000204178	TMEM57	-2.3207445	0.00024688
ENSG00000182831	C16orf72	-2.3189323	0.00687232
ENSG00000165233	CARD19	-2.3086297	0.00512586
ENSG00000070808	CAMK2A	-2.3083899	0.02065956
ENSG00000166747	AP1G1	-2.3035702	3.2365E-05
ENSG00000158882	TOMM40L	-2.3034862	0.00333586
ENSG00000173812	EIF1	-2.2977993	5.2951E-05
ENSG00000173334	TRIB1	-2.289118	0.00037874
ENSG00000161714	PLCD3	-2.2762185	0.00120954
ENSG00000110042	DTX4	-2.2758733	0.01231379
ENSG00000103966	EHD4	-2.2706792	0.00220767
ENSG00000256235	SMIM3	-2.2670807	0.00633024
ENSG00000137331	IER3	-2.2625199	9.7018E-05
ENSG00000186594	MIR22HG	-2.2617682	0.01271882
ENSG00000147872	PLIN2	-2.2604177	0.01529729
ENSG00000135094	SDS	-2.2587265	0.00393322
ENSG00000154710	RABGEF1	-2.2579161	0.00573577
ENSG00000147454	SLC25A37	-2.2524847	0.00808829
ENSG00000054967	RELT	-2.2509272	0.00401152
ENSG00000131669	NINJ1	-2.2499543	0.00217054
ENSG00000182149	IST1	-2.2486295	0.00016228
ENSG00000015568	RGPD5	-2.2473691	0.02185054
ENSG00000136717	BIN1	-2.2422262	0.00011131
ENSG00000213923	CSNK1E	-2.2412193	0.00076375
ENSG00000005379	TSPOAP1	-2.2345228	0.02347435
ENSG00000164211	STARD4	-2.2336266	0.01070554
ENSG00000165804	ZNF219	-2.2328169	0.00045548
ENSG00000175606	TMEM70	-2.2287584	0.04537919
ENSG00000182957	SPATA13	-2.2211987	0.00342977
ENSG00000164056	SPRY1	-2.2195278	0.01833939
ENSG00000164715	LMTK2	-2.2135726	0.00079208
ENSG00000090339	ICAM1	-2.2057344	0.00461745
ENSG00000174437	ATP2A2	-2.2038848	0.00111639

ENSG00000068878	PSME4	-2.2033136	0.00114568
ENSG00000109066	TMEM104	-2.1988191	0.00428046
ENSG00000141506	PIK3R5	-2.1952702	0.00129345
ENSG00000121671	CRY2	-2.1947757	0.03182714
ENSG00000165006	UBAP1	-2.1913766	4.6415E-05
ENSG00000086666	ZFAND6	-2.1889773	0.00018529
ENSG00000136732	GYPC	-2.1856152	0.00024683
ENSG00000213639	PPP1CB	-2.1835705	0.00348599
ENSG00000152102	FAM168B	-2.1803401	0.00888977
ENSG00000116679	IVNS1ABP	-2.1759616	0.03850762
ENSG00000170989	S1PR1	-2.1745131	0.01271455
ENSG00000106546	AHR	-2.1643124	0.00319666
ENSG00000105722	ERF	-2.1626278	0.00134062
ENSG00000124198	ARFGEF2	-2.1572501	0.00049702
ENSG00000166128	RAB8B	-2.1568523	0.00023416
ENSG00000270681	AC095055.1	-2.1564927	0.00334425
ENSG00000057704	TMCC3	-2.155854	0.03222085
ENSG00000204977	TRIM13	-2.1521171	0.00013559
ENSG00000169629	RGPD8	-2.1506083	0.02394401
ENSG00000169251	NMD3	-2.1366503	0.0000369
ENSG00000107968	MAP3K8	-2.1358555	0.01085837
ENSG00000101782	RIOK3	-2.1322235	0.00026591
ENSG00000155307	SAMSN1	-2.1298181	0.03896169
ENSG00000106635	BCL7B	-2.1270893	0.00016228
ENSG00000173848	NET1	-2.1189954	0.00079537
ENSG00000197170	PSMD12	-2.1151412	0.00032825
ENSG00000119950	MXI1	-2.1135775	0.02516512
ENSG00000114784	EIF1B	-2.1078115	0.00079091
ENSG00000110047	EHD1	-2.1013485	0.00697519
ENSG00000152484	USP12	-2.1003625	0.00089264
ENSG00000183876	ARSI	-2.099386	0.0013479
ENSG00000166839	ANKDD1A	-2.099097	0.01754524
ENSG00000117000	RLF	-2.0980237	0.00075679
ENSG00000175130	MARCKSL1	-2.0916263	0.00105513
ENSG00000103365	GGA2	-2.0901133	0.00121006
ENSG00000102034	ELF4	-2.0898317	0.00326607
ENSG00000173846	PLK3	-2.0895813	0.00113894
ENSG00000110848	CD69	-2.0733829	0.01675735
ENSG00000168264	IRF2BP2	-2.071314	0.0026279
ENSG00000122877	EGR2	-2.0695338	0.00800203
ENSG00000110367	DDX6	-2.0645479	0.00088717
ENSG00000078269	SYNJ2	-2.0629506	0.02118512

ENSG00000263020	AL662899.2	-2.0572586	0.04147729
ENSG00000102265	TIMP1	-2.0567885	0.00349848
ENSG00000100614	PPM1A	-2.0557918	8.4055E-05
ENSG00000177706	FAM20C	-2.0534868	0.028623
ENSG00000126705	AHDC1	-2.0525454	0.01052401
ENSG00000125733	TRIP10	-2.0485752	0.01502992
ENSG00000198805	PNP	-2.0454355	0.03460549
ENSG00000186431	FCAR	-2.0449644	0.03493191
ENSG00000145675	PIK3R1	-2.0436209	0.0444155
ENSG00000145632	PLK2	-2.0431337	0.03173833
ENSG00000149115	TNKS1BP1	-2.0344923	0.00977956
ENSG00000020633	RUNX3	-2.0304191	0.00303617
ENSG00000132326	PER2	-2.0278655	0.00143466
ENSG00000135404	CD63	-2.0257583	0.00303568
ENSG00000155304	HSPA13	-2.0245682	0.00124066
ENSG00000171316	CHD7	-2.0198256	0.0001798
ENSG00000116786	PLEKHM2	-2.0185337	0.00365448
ENSG00000101413	RPRD1B	-2.0152206	0.00032015
ENSG00000173744	AGFG1	-2.0128491	0.00367787
ENSG00000171867	PRNP	-2.008113	0.00149954
ENSG00000153914	SREK1	-2.0060482	0.00187327
ENSG00000185650	ZFP36L1	-2.0056671	0.01145874
ENSG00000186174	BCL9L	-2.002974	0.01942106
ENSG00000168214	RBPJ	-2.0013176	0.00293188
ENSG00000023287	RB1CC1	-1.9931023	0.00702366
ENSG00000132661	NXT1	-1.9919591	0.04292691
ENSG00000204160	ZDHHC18	-1.9917883	0.00149954
ENSG00000172071	EIF2AK3	-1.9917504	0.00253835
ENSG00000153066	TXNDC11	-1.9897945	0.00119443
ENSG00000176845	METRNL	-1.9892546	0.0068429
ENSG00000186660	ZFP91	-1.9889859	0.0000369
ENSG00000069956	MAPK6	-1.9881321	0.00203372
ENSG00000107816	LZTS2	-1.9862474	0.00036847
ENSG00000123091	RNF11	-1.9860266	2.4517E-05
ENSG00000196923	PDLIM7	-1.9855536	0.00080795
ENSG00000033327	GAB2	-1.9828257	0.00425917
ENSG00000139832	RAB20	-1.9828184	0.00775912
ENSG00000174738	NR1D2	-1.9794011	0.00432981
ENSG00000186642	PDE2A	-1.9782233	0.02931326
ENSG00000198900	TOP1	-1.9740894	0.0079006
ENSG00000145241	CENPC	-1.9740236	0.00061876
ENSG00000107554	DNMBP	-1.9723214	0.01715119

ENSG00000183735	TBK1	-1.9713113	1.8312E-05
ENSG00000100083	GGA1	-1.9684336	0.0001054
ENSG00000165030	NFIL3	-1.9683506	0.0001798
ENSG00000163877	SNIP1	-1.9677867	0.00134844
ENSG00000110713	NUP98	-1.9636771	0.00253039
ENSG00000125812	GZF1	-1.9620554	0.02372848
ENSG00000110852	CLEC2B	-1.9618504	0.00497045
ENSG00000165424	ZCCHC24	-1.9606687	0.00861902
ENSG00000118515	SGK1	-1.9587755	0.03928864
ENSG00000111641	NOP2	-1.9578295	0.030491
ENSG00000126368	NR1D1	-1.9572148	0.01145314
ENSG00000117139	KDM5B	-1.9569944	0.00217905
ENSG00000134107	BHLHE40	-1.9566723	0.01264053
ENSG00000111615	KRR1	-1.954116	0.0008326
ENSG00000280138	AC027290.2	-1.9507009	0.00039116
ENSG00000083799	CYLD	-1.950076	0.00022505
ENSG00000137166	FOXP4	-1.949993	0.00140031
ENSG00000145780	FEM1C	-1.9471062	0.00023497
ENSG00000082153	BZW1	-1.9431389	0.01711668
ENSG00000107863	ARHGAP21	-1.9402569	6.1348E-05
ENSG00000036054	TBC1D23	-1.9309444	0.00022218
ENSG00000133789	SWAP70	-1.9304165	0.00333076
ENSG00000123360	PDE1B	-1.9295376	0.00186871
ENSG00000186918	ZNF395	-1.9273649	0.00392283
ENSG00000125772	GPCPD1	-1.9239971	0.00387853
ENSG00000170638	TRABD	-1.9202134	0.00016159
ENSG00000083896	YTHDC1	-1.9190759	8.2108E-05
ENSG00000064012	CASP8	-1.912928	0.00168434
ENSG00000184216	IRAK1	-1.9121738	0.01178924
ENSG00000253276	CCDC71L	-1.911114	0.00254656
ENSG00000099381	SETD1A	-1.9071814	0.02676969
ENSG00000162924	REL	-1.9040934	3.2365E-05
ENSG00000154978	VOPP1	-1.9028077	0.01537657
ENSG00000055609	KMT2C	-1.8989444	0.02105759
ENSG00000160741	CRTC2	-1.8987936	0.0002735
ENSG00000240849	TMEM189	-1.8954654	0.01891436
ENSG00000196233	LCOR	-1.8941411	0.00006077
ENSG00000079332	SAR1A	-1.8930478	0.00037122
ENSG00000019995	ZRANB1	-1.8898784	0.00046556
ENSG00000109113	RAB34	-1.8894695	0.0012744
ENSG00000101558	VAPA	-1.8860457	0.04326385
ENSG00000101421	CHMP4B	-1.8811711	0.00179355

ENSG00000163785	RYK	-1.8750924	0.00092722
ENSG00000272886	DCP1A	-1.8731265	0.00157429
ENSG00000118689	FOXO3	-1.8706899	0.00959379
ENSG00000121749	TBC1D15	-1.8704506	0.00049264
ENSG00000196843	ARID5A	-1.8689803	0.00450279
ENSG00000211459	MT-RNR1	-1.8644215	0.00135837
ENSG00000173276	ZBTB21	-1.8617309	0.00147052
ENSG00000181045	SLC26A11	-1.8598453	0.01978584
ENSG00000112406	HECA	-1.8598173	0.00092214
ENSG00000105656	ELL	-1.8583687	0.00732956
ENSG00000112282	MED23	-1.8571229	0.00162936
ENSG00000138166	DUSP5	-1.8569247	0.00086712
ENSG00000169762	TAPT1	-1.8516965	0.0003077
ENSG00000163050	COQ8A	-1.8501052	0.00675146
ENSG00000126775	ATG14	-1.8468722	0.00024848
ENSG00000132912	DCTN4	-1.8468371	0.00010145
ENSG00000100284	TOM1	-1.846443	0.00153216
ENSG00000167996	FTH1	-1.8459891	0.00235438
ENSG00000023734	STRAP	-1.8458261	0.0000369
ENSG00000183955	KMT5A	-1.8436599	0.00498639
ENSG00000186834	HEXIM1	-1.8421121	0.00571373
ENSG00000166165	CKB	-1.8411489	0.00802101
ENSG00000065809	FAM107B	-1.8409382	0.00187327
ENSG00000198853	RUSC2	-1.8374819	0.00601804
ENSG00000160570	DEDD2	-1.8355286	0.00329172
ENSG00000181220	ZNF746	-1.8354554	0.00622101
ENSG00000008952	SEC62	-1.8310351	0.00041379
ENSG00000169967	MAP3K2	-1.8276966	0.01488026
ENSG00000258890	CEP95	-1.8171787	0.00297137
ENSG00000167491	GATAD2A	-1.8153259	0.00293188
ENSG00000122068	FYTTD1	-1.8128755	0.0002456
ENSG00000132155	RAF1	-1.8089564	0.00063722
ENSG00000168438	CDC40	-1.8080239	0.00339332
ENSG00000255112	CHMP1B	-1.8052809	0.00601804
ENSG00000140564	FURIN	-1.7987076	0.01662645
ENSG00000140941	MAP1LC3B	-1.7985835	0.00108044
ENSG00000156650	KAT6B	-1.7985556	0.00043898
ENSG00000103657	HERC1	-1.7963983	0.00025626
ENSG00000132510	KDM6B	-1.795885	0.0001275
ENSG00000176624	MEX3C	-1.7956592	0.00010911
ENSG00000104081	BMF	-1.7933229	0.0037225
ENSG00000101544	ADNP2	-1.7924296	0.00071567

ENSG00000047634	SCML1	-1.7920938	0.00018529
ENSG00000196396	PTPN1	-1.7911115	0.00848506
ENSG00000101216	GMEB2	-1.7898481	0.00651799
ENSG00000170677	SOCS6	-1.7869862	0.01133185
ENSG00000106723	SPIN1	-1.786127	0.00392897
ENSG00000138069	RAB1A	-1.7825402	0.0046341
ENSG00000141580	WDR45B	-1.7784459	0.00614734
ENSG00000139725	RHOF	-1.7777834	0.00807752
ENSG00000126524	SBDS	-1.7750369	0.00087231
ENSG00000183431	SF3A3	-1.7741834	0.0004242
ENSG00000164687	FABP5	-1.772035	0.00229042
ENSG00000162664	ZNF326	-1.7703503	0.00016166
ENSG00000164442	CITED2	-1.7700743	0.00254922
ENSG00000144655	CSRNP1	-1.7700244	0.01231379
ENSG00000160408	ST6GALNAC6	-1.7689971	0.00179958
ENSG00000109118	PHF12	-1.7661587	0.00072397
ENSG00000106346	USP42	-1.7657116	0.00231527
ENSG00000102984	ZNF821	-1.7654559	0.00205786
ENSG00000101384	JAG1	-1.7652708	0.00023497
ENSG00000132334	PTPRE	-1.7617087	0.00829848
ENSG00000100425	BRD1	-1.7605867	0.0001505
ENSG00000127838	PNKD	-1.7604756	0.03950871
ENSG00000178127	NDUFV2	-1.7603756	0.00073001
ENSG00000164823	OSGIN2	-1.753502	0.00033773
ENSG00000124762	CDKN1A	-1.7524954	0.00250115
ENSG00000197183	NOL4L	-1.749076	0.02292124
ENSG00000104885	DOT1L	-1.7490404	0.00170844
ENSG00000156860	FBRS	-1.749037	0.0011299
ENSG00000178789	CD300LB	-1.7414884	0.02436108
ENSG00000242125	SNHG3	-1.7411488	0.00077068
ENSG00000072274	TFRC	-1.7400163	0.04603645
ENSG00000027697	IFNGR1	-1.7393537	0.0136665
ENSG00000183484	GPR132	-1.7384703	0.00125237
ENSG00000146872	TLK2	-1.738412	0.00011229
ENSG00000139697	SBNO1	-1.7374031	0.00493368
ENSG00000175556	LONRF3	-1.7374001	7.6536E-05
ENSG00000131051	RBM39	-1.7358744	0.00069566
ENSG00000090924	PLEKHG2	-1.7317967	0.00258906
ENSG00000114796	KLHL24	-1.7293225	0.0001253
ENSG00000268734	AC245128.3	-1.7288244	0.03527874
ENSG00000159461	AMFR	-1.7251031	0.00459037
ENSG00000134815	DHX34	-1.7200533	0.00094463

ENSG00000106004	HOXA5	-1.7157683	0.01131822
ENSG00000174574	AKIRIN1	-1.7154807	0.00059211
ENSG00000100100	PIK3IP1	-1.7144539	0.00698448
ENSG00000119801	YPEL5	-1.7132154	0.0001146
ENSG00000138593	SECISBP2L	-1.7125599	0.00014906
ENSG00000183337	BCOR	-1.7104719	0.0451965
ENSG00000080371	RAB21	-1.7089091	0.00018222
ENSG00000008294	SPAG9	-1.7086175	0.00105353
ENSG00000076924	XAB2	-1.7069058	0.00044978
ENSG00000145495	38777	-1.7043489	0.0003874
ENSG00000140367	UBE2Q2	-1.7043335	0.00029716
ENSG00000177169	ULK1	-1.701854	6.3157E-05
ENSG00000164327	RICTOR	-1.7017626	0.00133432
ENSG00000163697	APBB2	-1.7010362	0.02373579
ENSG00000150593	PDCD4	-1.7007338	0.00040152
ENSG00000036257	CUL3	-1.7001997	0.00072261
ENSG00000196428	TSC22D2	-1.6986638	0.00068123
ENSG00000143217	NECTIN4	-1.698404	0.00022322
ENSG00000213722	DDAH2	-1.6968848	0.0022176
ENSG00000155926	SLA	-1.6968623	0.03257164
ENSG00000163605	PPP4R2	-1.695595	0.00032825
ENSG00000169895	SYAP1	-1.6955365	0.01716858
ENSG00000087206	UIMC1	-1.6949801	0.00035769
ENSG00000013441	CLK1	-1.694474	0.00031514
ENSG00000171988	JMJD1C	-1.6918273	0.00078546
ENSG00000075415	SLC25A3	-1.6912662	0.00151518
ENSG00000124486	USP9X	-1.6910736	0.00016587
ENSG00000259330	INAFM2	-1.6891145	0.00217509
ENSG00000188215	DCUN1D3	-1.6882633	0.00039781
ENSG00000231259	AC125232.1	-1.6869602	0.01216003
ENSG00000197818	SLC9A8	-1.6864555	0.01216003
ENSG00000114120	SLC25A36	-1.6853343	0.00396268
ENSG00000008256	CYTH3	-1.6823948	0.00671091
ENSG00000102225	CDK16	-1.68182	0.0067314
ENSG00000122862	SRGN	-1.6814337	0.00363392
ENSG00000150907	FOXO1	-1.6803792	0.0011299
ENSG00000197063	MAFG	-1.6756863	0.01274117
ENSG00000067182	TNFRSF1A	-1.6720402	1.2095E-05
ENSG00000150457	LATS2	-1.6711013	0.00114234
ENSG00000165813	CCDC186	-1.6705424	0.00225444
ENSG00000166484	MAPK7	-1.6667525	0.0052913
ENSG00000143514	TP53BP2	-1.6645397	0.00098261

ENSG00000168615	ADAM9	-1.6638168	0.0008965
ENSG00000135093	USP30	-1.6631448	0.00340911
ENSG00000072364	AFF4	-1.6623323	3.816E-06
ENSG00000102119	EMD	-1.6621668	0.00070482
ENSG00000214174	AMZ2P1	-1.6618303	0.00776543
ENSG00000188994	ZNF292	-1.6564893	0.01019047
ENSG00000126767	ELK1	-1.6562438	7.4197E-05
ENSG00000131408	NR1H2	-1.6550241	0.00025405
ENSG00000167703	SLC43A2	-1.6507063	0.01025104
ENSG00000105325	FZR1	-1.6463611	0.00941259
ENSG00000117036	ETV3	-1.6441059	0.0422585
ENSG00000130517	PGPEP1	-1.640259	0.00108282
ENSG00000159346	ADIPOR1	-1.6401029	0.00077849
ENSG00000117569	PTBP2	-1.6397044	0.00083979
ENSG00000118707	TGIF2	-1.6382022	0.00171347
ENSG00000123066	MED13L	-1.636608	0.00066794
ENSG00000176170	SPHK1	-1.6363195	0.0084539
ENSG00000130695	CEP85	-1.6359461	0.00590266
ENSG00000102908	NFAT5	-1.6359083	0.00269425
ENSG00000140044	JDP2	-1.6348555	0.01099759
ENSG00000109332	UBE2D3	-1.6330588	0.00072397
ENSG00000175197	DDIT3	-1.632968	0.033075
ENSG00000135503	ACVR1B	-1.6323848	0.0008674
ENSG00000087087	SRRT	-1.629347	0.01770152
ENSG00000150403	TMCO3	-1.6286247	0.00081798
ENSG00000178607	ERN1	-1.6281895	0.00018294
ENSG00000105856	HBP1	-1.6271893	0.00005434
ENSG00000267520	AC010733.2	-1.6271018	0.00235373
ENSG00000140379	BCL2A1	-1.6264128	0.00184798
ENSG00000197386	HTT	-1.6249569	0.03190124
ENSG00000280987	MATR3	-1.6242489	0.03360204
ENSG00000056972	TRAF3IP2	-1.6234054	0.00461753
ENSG00000111011	RSRC2	-1.6228625	0.00059557
ENSG00000135968	GCC2	-1.6219996	0.00934996
ENSG00000147526	TACC1	-1.6202835	0.0013507
ENSG00000005486	RHBDD2	-1.6201412	0.01186484
ENSG00000115165	CYTIP	-1.6199146	0.00976103
ENSG00000172766	NAA16	-1.6173609	0.00064718
ENSG00000145860	RNF145	-1.6171896	0.00100657
ENSG00000156381	ANKRD9	-1.6163393	0.02888984
ENSG00000069399	BCL3	-1.6159176	0.01288218
ENSG00000143373	ZNF687	-1.6152912	0.03419193

ENSG00000116584	ARHGEF2	-1.6134978	0.01483446
ENSG00000179051	RCC2	-1.6127476	0.01160789
ENSG00000124766	SOX4	-1.6114084	0.00223409
ENSG00000044574	HSPA5	-1.6099202	0.00492287
ENSG00000145979	TBC1D7	-1.6092439	0.00847841
ENSG00000139433	GLTP	-1.6066986	0.00417663
ENSG00000138867	GUCD1	-1.6063594	0.00169689
ENSG00000198925	ATG9A	-1.6058816	0.00784433
ENSG00000032219	ARID4A	-1.6058472	0.00016587
ENSG00000241839	PLEKHO2	-1.6046012	0.00403476
ENSG00000100221	JOSD1	-1.6035386	0.01667953
ENSG00000114098	ARMC8	-1.6017833	7.5228E-05
ENSG00000178623	GPR35	-1.6007657	0.0312656
ENSG00000136830	FAM129B	-1.5973004	0.00921876
ENSG00000005483	KMT2E	-1.5948804	0.00019371
ENSG00000136807	CDK9	-1.5938694	0.00702732
ENSG00000105655	ISYNA1	-1.5926978	0.00582601
ENSG00000138434	SSFA2	-1.5911581	0.00017899
ENSG00000115956	PLEK	-1.591103	0.0025654
ENSG00000055208	TAB2	-1.5910037	0.00017634
ENSG00000120690	ELF1	-1.589289	0.0006017
ENSG00000107937	GTPBP4	-1.5890128	0.00428685
ENSG00000023330	ALAS1	-1.5889304	0.04777731
ENSG00000115339	GALNT3	-1.587047	0.01936306
ENSG00000152409	JMY	-1.586805	0.00037088
ENSG00000270069	MIR222HG	-1.5859323	0.00096657
ENSG00000116260	QSOX1	-1.5833351	0.00220839
ENSG00000043093	DCUN1D1	-1.5832245	0.00075096
ENSG00000197122	SRC	-1.5803507	0.00058719
ENSG00000135241	PNPLA8	-1.5795546	0.0024376
ENSG00000124782	RREB1	-1.5782724	0.00106505
ENSG00000127824	TUBA4A	-1.5779967	0.00708238
ENSG00000198369	SPRED2	-1.5763408	0.0016113
ENSG00000151247	EIF4E	-1.5752877	0.0004212
ENSG00000173960	UBXN2A	-1.5744389	0.00037874
ENSG00000184428	TOP1MT	-1.5739683	0.01145314
ENSG00000240053	LY6G5B	-1.5730019	0.00483369
ENSG00000140743	CDR2	-1.5723227	0.00416262
ENSG00000168066	SF1	-1.5711265	0.00248889
ENSG00000160179	ABCG1	-1.5694762	0.00069117
ENSG00000064961	HMG20B	-1.5673559	0.00040591
ENSG00000154124	OTULIN	-1.5673035	0.00123872

ENSG00000181222	POLR2A	-1.566575	0.00159885
ENSG00000076108	BAZ2A	-1.5661032	0.00076885
ENSG00000076604	TRAF4	-1.5658535	0.01836102
ENSG00000074416	MGLL	-1.5650616	0.02306784
ENSG00000159082	SYNJ1	-1.5626197	0.00604553
ENSG00000153561	RMND5A	-1.5626103	0.00119944
ENSG00000186469	GNG2	-1.5612618	0.01606603
ENSG00000152700	SAR1B	-1.5608848	0.00029457
ENSG00000239857	GET4	-1.559559	0.0017433
ENSG00000113712	CSNK1A1	-1.5593144	0.00022731
ENSG00000260708	AL118516.1	-1.5586566	0.00633623
ENSG00000102007	PLP2	-1.5573676	0.00035647
ENSG00000112033	PPARD	-1.5559943	0.00175002
ENSG00000147439	BIN3	-1.555449	0.00188738
ENSG00000198833	UBE2J1	-1.5532614	0.00794965
ENSG00000137817	PARP6	-1.5530293	0.00011008
ENSG00000105968	H2AFV	-1.5522882	0.00025717
ENSG00000117614	SYF2	-1.5513175	8.5435E-05
ENSG00000155508	CNOT8	-1.5510955	0.001272
ENSG00000162923	WDR26	-1.5504078	0.00013674
ENSG00000116285	ERRFI1	-1.5503939	0.00773351
ENSG00000164169	PRMT9	-1.5500579	0.00051771
ENSG00000113575	PPP2CA	-1.5499641	0.00062165
ENSG00000197780	TAF13	-1.5496012	0.00091009
ENSG00000262246	CORO7	-1.5483429	0.00958444
ENSG00000166233	ARIH1	-1.5479744	0.00015176
ENSG00000244486	SCARF2	-1.5463953	0.0210235
ENSG00000128989	ARPP19	-1.5457656	0.00077102
ENSG00000164543	STK17A	-1.5455914	0.0392918
ENSG00000131759	RARA	-1.5448456	0.01590993
ENSG00000276107	AC037198.2	-1.5440499	0.00238135
ENSG00000139636	LMBR1L	-1.5439465	0.00464824
ENSG00000167604	NFKBID	-1.5436091	0.00256234
ENSG00000197405	C5AR1	-1.542184	0.00477562
ENSG00000070495	JMJD6	-1.5394051	0.0030835
ENSG00000092847	AGO1	-1.5393011	0.00526619
ENSG00000105821	DNAJC2	-1.5391475	0.00675342
ENSG00000119048	UBE2B	-1.5368156	0.00043714
ENSG00000156671	SAMD8	-1.5360163	0.00349533
ENSG00000139505	MTMR6	-1.5350177	0.00115962
ENSG00000164548	TRA2A	-1.5338747	0.00011163
ENSG00000029993	HMGB3	-1.5316349	0.01029999

ENSG00000185947	ZNF267	-1.5298207	0.00454279
ENSG00000273356	LINC02019	-1.5292778	0.00119944
ENSG00000141985	SH3GL1	-1.5280713	0.00588359
ENSG00000128272	ATF4	-1.5280042	5.2951E-05
ENSG00000172216	CEBPB	-1.527772	0.02014986
ENSG00000158669	GPAT4	-1.5268625	0.00166041
ENSG00000188070	C11orf95	-1.5266466	0.02506593
ENSG00000111252	SH2B3	-1.5263865	0.00860229
ENSG00000065357	DGKA	-1.5260897	0.0002973
ENSG00000167671	UBXN6	-1.5259656	0.00109223
ENSG00000169905	TOR1AIP2	-1.5249436	0.00597502
ENSG00000147119	CHST7	-1.5236811	0.00789381
ENSG00000167034	NKX3-1	-1.5236105	0.00062255
ENSG00000161638	ITGA5	-1.5223195	4.6944E-05
ENSG00000154359	LONRF1	-1.5210642	0.00063116
ENSG00000143067	ZNF697	-1.5168708	7.6536E-05
ENSG00000230551	AC021078.1	-1.5167031	0.00229423
ENSG00000166974	MAPRE2	-1.5133908	0.00017899
ENSG00000179119	SPTY2D1	-1.512944	0.00042489
ENSG00000067596	DHX8	-1.512099	0.00033588
ENSG00000137876	RSL24D1	-1.5114195	0.00105092
ENSG00000137947	GTF2B	-1.510406	0.00011985
ENSG00000108669	CYTH1	-1.505954	0.00110115
ENSG00000149782	PLCB3	-1.5047599	0.00626591
ENSG00000144711	IQSEC1	-1.50364	0.0020674
ENSG00000133794	ARNTL	-1.5018533	0.00050819
ENSG00000067900	ROCK1	-1.5006315	0.00019992

Appendix Table S5. Reagents and Tools Table

Reagent/Resource	Reference or Source	Identifier or Catalog Number
**Experimental Models**		
C57BL/6 Tyk2-/- mice	Dr. M. Müller, Viena, Austria	
C57BL/6 mice	Jackson Lab.	
K562-GFP line cell	NIH Reagent Program	116799
**Recombinant DNA**		
**Antibodies**		
Anti-human CD3	Biolegend	300327
Anti-human CD19	Biolegend	363015
Anti-human CD20	Biolegend	302325
Anti-human CD56	Biolegend	362505
Anti-human CD14	BD Bioscience	557831
Anti-human CD16	Biolegend	302021
Anti-human CD56	Biolegend	362524
Anti-human HLA-DR	Biolegend	307623
Anti-human CD11c	Biolegend	301625
Anti-human MICA/B	Biolegend	320912
Anti-human CD40	Biolegend	334306
Anti-human CD86	Biolegend	374210
Anti-human IFNγ	Biolegend	554551
Anti-human CD141	Biolegend	344105
Anti-human TNFa	Biolegend	502923
Anti-human CD107a	Biolegend	328620
Anti-human CD1c	Biolegend	331515
Anti-human CD64	Biolegend	305131
Anti-human ULBP1	RyD systems	FAB1380P
Anti-human CD1c	BD Bioscience	564900
Anti-human PCNA	Biolegend	307904
Streptavidin	BD Bioscience	561419
Anti-human HLADR	Biolegend	307618
Anti-human CD3	Biolegend	300317
Anti-human CD19	Biolegend	302217
Anti-human NKG2A	RyD Systems	FAB1059C-025
Anti-human NKG2D	Biolegend	320819

Anti-human NKp30	Biolegend	325213
Anti-human PD-L1	Biolegend	329721
Anti-human CD14	Biolegend	367113
Anti-human CD123	BD Bioscience	560087
Anti-human CD86	Biolegend	305427
Anti-human CD16	Biolegend	302045
Ghost DyeTM	Tonbo Biosciences	13-0865-T100
Yellow fluorescent reactive dye	Invitrogen	L34959 A
Anti-mouse MHCII	Biolegend	107652
Anti-mouse CD3	Tonbo Biosciences	35-0031
Anti-mouse CD11b	Tonbo Biosciences	75-0112
Anti-mouse CD27	Biolegend	124216
Anti-mouse NK1.1	Tonbo Biosciences	50-5941
Anti-mouse CD45.2	eBioscience	17-0454-81
Anti-mouse CD64	BD Bioscience	558539
Anti-mouse MHCII	BD Bioscience	553623
Anti-mouse CD11c	BD Bioscience	565452
Anti-mouse CD11b	eBioscience	25-0112-81
Anti-mouse XCR1	Biolegend	148224
Anti-mouse Ly6C	BD Bioscience	560525
Anti-mouse CD3	BD Bioscience	563123
Anti-mouse CD107a	Biolegend	121610
Anti-mouse IFNγ	eBioscience	11-7311-82
Anti-mouse CD45.2	BD Bioscience	563686
Anti-mouse NKG2D	ThermoFisher	46-5882-82
Anti-mouse RAE-1	Biolegend	748218
Goat anti-human MICa/b	RyD Systems	MAB13001
Human IL-15 Antibody	RyD - Biotechne	MAB2471-100
IgG2b Isotype Control	BioLegend	402202
IgG1 Isotype Control	BioLegend	401402
Violet Cell Proliferation Tracker	ThermoFisher	C34557
Mouse anti-human CD56	Invitrogen	MA1-06801
Goat anti-human CD56	RyD Systems	AF2408
Mouse anti-human CD1c	abcam	Ab156708
Rabbit anti-human HLA Class II	abcam	Ab133578
Rat anti-human CD19	Invitrogen	14-0194-80
Goat anti-human IL-17a	RyD Systems	AF-317-SP
Goat anti-human MICa/b	RyD Systems	MAB13001

Granzyme B antibody	Thermo Fisher	14-8889-80
Donkey anti-rabbit AF488	Thermo Fisher	R37118
Donkey anti-goat AF488	Thermo Fisher	A-11055
Donkey anti-rat AF594	Jackson ImmunoResearch	712-586-150
Donkey anti-goat AF568	Thermo Fisher	A-11057
Donkey anti-goat AF647	Thermo Fisher	A-21447
Donkey anti-mouse AF647	Thermo Fisher	A-31571
Rabbit anti-mouse PAX5 antibody	Abcam	Ab109443
**Oligonucleotides and other sequence-based reagents**		
Human DDX60	This study	Methods
Human DDX58 (RIG-I)	This study	Methods
Human IFIT1	This study	Methods
Human STAT1	This study	Methods
Human IL-15	This study	Methods
Human DDX60 siRNA SMART pool	Dharmacon	L-017664-01-0005
Human DDX58 (RIG-I) *siRNA SMART pool*	Dharmacon	L-012511-00-0005
Human IFIT1 *siRNA SMART pool*	Dharmacon	L-019616-00-0005
Human STAT1 *siRNA SMART pool*	Dharmacon	L-003543-00-0005
**Chemicals, Enzymes and other reagents**		
Polyinosinic-polycytidylic acid sodium salt (poly I:C)	Merck- Sigma	P1530
Brefeldin A	Sigma-Aldrich (Merck)	B7651
Monensin	Sigma-Aldrich (Merck)	
Liberase TL	Roche	5401020001
DNAase I	Roche	11284932001
Pilocarpine hydrochloride	Sigma-Aldrich (Merck)	54-71-7
Ketamina 100 mg/ml	Pfizer	47034
Xilacina 20mg/ml	CALIER	Ref. 20100-005
Anti-PE MicroBeads	Miltenyi Biotec	130-048-801
Easy step human NK cell Isolation Kit	Stemcell	17955
Easy step™ human myeloid DC enrichment kit	Stemcell	19061
CD11c MicroBeads UltraPure, mouse	Miltenyi Biotec	130-108-338
PMA	Sigma Aldrich (Merck)	P1585
Ionomicin	Sigma Aldrich (Merck)	I0634-1MG
**Software**		
QuPath v0.4.3		
FlowJo v10		
Image J		
Gradpad Prism 9.0		

**Other**		
Illumina NextSeq	San Diego, CA	
Zeiss microscopy		
Leica TCS SP5 confocal	Leica	
