# Supplementary material for: MICa/b‐dependent activation of natural killer cells by CD64 + inflammatory type 2 dendritic cells contributes to autoimmunity
Source: EMBO J. 2023 Nov 2;42(23):e113714. doi: 10.15252/embj.2023113714 (PMC10690448; doi:10.15252/embj.2023113714)
Supplement: Supplementary file 7 [file EMBJ-42-e113714-s003.pdf]

THE EMBO JOURNAL

MICa/b-dependent Activation of Natural Killer Cells by CD64+ inflammatory type 2 dendritic cells Contributes to Autoimmunity

Ildefonso Sánchez-Cerrillo, Diego Calzada-Fraile, Ana Triguero-Martínez, Marta Calvet-Mirabent, Olga Popova, Cristina Delgado-Arévalo, Mariel Valdivia-Mazeyra, Marta Ramirez-Huesca, Enrique Vázquez, Alberto Benguría, Teresa Aceña Gonzalo, Roberto Moreno-Vellisca, Magdalena Adrados de Llano, Hortensia de la Fuente, Ilya Tsukalov, Pablo Delgado Wicke, Elena Fernández Ruiz, Emilia Roy-Vallejo, Reyes Tejedor Lázaro, Almudena Ramiro, Salvador Iborra, Francisco Sánchez-Madrid, Ana Dopazo, Isidoro González-Álvaro, Santos Castañeda, and Enrique Martin-Gayo
DOI: 10.15252/embj.2023113714

Corresponding author(s): Enrique Martin-Gayo (enrique.martin@uam.es)

Review Timeline:

Submission Date:	8th Feb 23
Editorial Decision:	22nd Mar 23
Revision Received:	7th Jul 23
Editorial Decision:	6th Sep 23
Revision Received:	27th Sep 23
Accepted:	5th Oct 23

Editor: Karin Dumstrei

Transaction Report:

(Note: With the exception of the correction of typographical or spelling errors that could be a source of ambiguity, letters and reports are not edited. Depending on transfer agreements, referee reports obtained elsewhere may or may not be included in this compilation. Referee reports are anonymous unless the Referee chooses to sign their reports.)

1st Editorial Decision **22nd Mar 2023**

Dear Enrique,

Thank you for submitting your manuscript to The EMBO Journal. Your manuscript was transferred to The EMBO Journal with referee reports from another journal. I involved an arbitrating advisor to look at the manuscript, original referee comments and your point-by-point response.

I am sorry for the delay in getting back to you with a decision but have now received the needed input to take a decision on the manuscript. This also involved additional consultations with the advisor.

Both the advisor and I see the potential of the study, but further analysis is also needed for consideration here. See specific comments below.

As you have data on hand, or able to address many of the raised concerns, I would be open to consider a revised version.

What we suggest is to focus the manuscript on the dataset from pSS patients and remove the poly I:C mouse data as it is not a bonafide Sjogren's Syndrome model. All the referees that have evaluated the paper comments on this aspect and I find that this dataset is almost a distraction from the key parts of the paper.

Please address the original referees' concerns and points raised below eg how DC and NK cells interact and the role of cytokine production and IL-15 in this process. Do you have any further support/insight into how cytolytic NK cells contribute to disease pathology?

Also, can you stratify of your patient data set and see if cDC2 and NK cells correlate with disease severity, treatment (referee #1 and 3)? Just to get some insight into the clinical relevance of the key findings.

If you re-structure the paper in this way, then I would also suggest that you start out with a broader description of the pSS patient dataset. Did you look at other immune cells etc.

When preparing your letter of response to the referees' comments, please bear in mind that this will form part of the Review Process File, and will therefore be available online to the community. For more details on our Transparent Editorial Process, please visit our website: https://www.embopress.org/page/journal/14602075/authorguide#transparentprocess

I hope that you find these comments helpful. Happy to discuss them further!

with best wishes

Karin

Karin Dumstrei, PhD
Senior Editor
The EMBO Journal

Instructions for preparing your revised manuscript:

Please make sure you upload a letter of response to the referees' comments together with the revised manuscript.

Please also check that the title and abstract of the manuscript are brief, yet explicit, even to non-specialists.

When assembling figures, please refer to our figure preparation guideline in order to ensure proper formatting and readability in print as well as on screen:
https://bit.ly/EMBOPressFigurePreparationGuideline
See also guidelines for figure legends: https://www.embopress.org/page/journal/14602075/authorguide#figureformat

At EMBO Press we ask authors to provide source data for the main manuscript figures. Our source data coordinator will contact you to discuss which figure panels we would need source data for and will also provide you with helpful tips on how to upload and organize the files.

IMPORTANT: When you send the revision we will require
- a point-by-point response to the referees' comments, with a detailed description of the changes made (as a word file).
- a word file of the manuscript text.
- individual production quality figure files (one file per figure)

- a complete author checklist, which you can download from our author guidelines (https://www.embopress.org/page/journal/14602075/authorguide).
- Expanded View files (replacing Supplementary Information)
Please see out instructions to authors
https://www.embopress.org/page/journal/14602075/authorguide#expandedview

Please remember: Digital image enhancement is acceptable practice, as long as it accurately represents the original data and conforms to community standards. If a figure has been subjected to significant electronic manipulation, this must be noted in the figure legend or in the 'Materials and Methods' section. The editors reserve the right to request original versions of figures and the original images that were used to assemble the figure.

Further information is available in our Guide For Authors: https://www.embopress.org/page/journal/14602075/authorguide

We realize that it is difficult to revise to a specific deadline. In the interest of protecting the conceptual advance provided by the work, we recommend a revision within 3 months (20th Jun 2023). Please discuss the revision progress ahead of this time with the editor if you require more time to complete the revisions as we can extend the revision time.

As a matter of policy, competing manuscripts published during this period will not negatively impact on our assessment of the conceptual advance presented by your study. However, we request that you contact the editor as soon as possible upon publication of any related work, to discuss how to proceed.

Use the link below to submit your revision:

https://emboj.msubmit.net/cgi-bin/main.plex

Referee #1:

I reviewed the manuscript, the provided referee reports, and the authors response. My assessment of the manuscript including the authors' rebuttal is provided below. Please note that I have focused on key concerns.

The major concerns relate to the lack of mechanistic data and the use of a murine model that is not appropriate as it does not represent a model of autoimmune Sjogren's Syndrome.

Each of the original reviewers raise several major concerns broadly relating to the points noted above. The manuscript submitted to The EMBO Journal does not appear to address these concerns. The response to the referees' reports unfortunately does not provide any additional data despite the authors indicating in several of their responses that such data are already available. It would have been helpful for the manuscript to be revised to include the available data and the response to previous reviews; then the authors could have simply discussed what additional new data would be generated to address any outstanding key concerns. Given that the manuscript does not address previous concerns, and additional available data referred to is not provided, it is difficult to determine whether the additional information would address the concerns raised previously or indeed the main concerns noted above.

All previous reviews noted an absence of mechanistic data. They also included questions on the role of cytokines, especially IL-15, in the postulated NKG2D-mediated activation of NK cells through cDC2s (R1-Q6, R2-Q7, R3-paragraph 8). The authors are clear in noting that "we focus on the relevance of NK and DC interaction in Sjogren's Syndrome". In keeping with this, an understanding of how these interactions occur is important and should not be considered, as stated by the authors, outside the scope of the current study.

All the previous reviews noted that the use of poly I:C treated mice as an in vivo model of Sjogren's Syndrome is not appropriate (R1-Q5, R2-Q8, R3-Significance and paragraph 9). The authors somewhat acknowledge the limitations of their chosen model but remain convinced that it is appropriate as is allows them to investigate glandular hypofunction and immune infiltration. NK cell responses and activation of DCs and monocytes can be examined in a poly I:C treatment model, and in fact some of the responses observed would be expected. However, the model cannot be used to examine mechanisms relevant to Sjogren's Syndrome, a pathology that is autoimmune in nature. Nandula et al (PMID: 22672212, Ref 32) noted that hyposalivation in poly I:C treated mice is transient and likely the result of type I IFN and IL-6 interference with Ca(2+) mobilization directly within acinar cells - thus not autoimmune in nature. Therefore, this system is not suited to in vivo modelling of the autoreactive immune responses that drive glandular pathology in Sjogren's Syndrome.

Several additional concerns remain to be addressed, including of note how activated NK cells affect cDC2 fate; the authors indicate they have data to address this, but do not provide it.

In sum, this manuscript reports on interesting observations in samples from patients with Sjogren's Syndrome but does not provide evidence of how they relate to disease. The manuscript does not demonstrate that activated cytotoxic NK cells contribute to autoimmune pathology. How interactions between NK cells and cDC2s (via NKG2D) result in enhanced formation of autoreactive T and B cells remains unclear.

The concerns outlined above lead me to conclude that as submitted, the manuscript is not suited for publication in The EMBO Journal.

POINT BY POINT RESPONSE TO REVIEWER

EMBO J REVIEWER:

I reviewed the manuscript, the provided referee reports, and the authors response. My assessment of the manuscript including the authors' rebuttal is provided below. Please note that I have focused on key concerns.

The major concerns relate to the lack of mechanistic data and the use of a murine model that is not appropriate as it does not represent a model of autoimmune Sjogren's Syndrome.

We have now addressed the mechanistic concerns previously raised and further justified the use of the *in vivo* data as a model of innate RNA sensing/IFN activation useful to study NK and cDC2 crosstalk and subsequent activation of pathogenic adaptive immune cells. Below we have answered in detail each of the points made by the reviewer.

Each of the original reviewers raise several major concerns broadly relating to the points noted above. The manuscript submitted to The EMBO Journal does not appear to address these concerns. The response to the referees' reports unfortunately does not provide any additional data despite the authors indicating in several of their responses that such data are already available. It would have been helpful for the manuscript to be revised to include the available data and the response to previous reviews; then the authors could have simply discussed what additional new data would be generated to address any outstanding key concerns. Given that the manuscript does not address previous concerns, and additional available data referred to is not provided, it is difficult to determine whether the additional information would address the concerns raised previously or indeed the main concerns noted above.

Since we have now performed all requested experimental work, we are providing the additional data for the previously raised concerns and we have modified figures accordingly; in addition, we have updated and further justified arguments made in response to previous reviewers.

All previous reviews noted an absence of mechanistic data. They also included questions on the role of cytokines, especially IL-15, in the postulated NKG2D-mediated activation of NK cells through cDC2s (R1-Q6, R2-Q7, R3-paragraph 8). The authors are clear in noting that "we focus on the relevance of NK and DC interaction in Sjogren's Syndrome". In keeping with this, an understanding of how these interactions occur is important and should not be considered, as stated by the authors, outside the scope of the current study.

We thank the reviewer for pointing out this issue. We have now provided more detailed mechanistic data concerning the following major analyses:

1-We have further studied the role of signaling downstream the RIG-I/DDX60 RNA sensors such as IFN receptor and ISGs as well as the dependence of other cell

types in the regulation of the expression of MICa/b in cDC2 after exposure to Poly I:C. Specifically, we have analyzed the role of STAT1 as a molecule required for IFN I and IFN II receptors signaling, and further studied IFN I receptor using mice deficient for Tyk2, an adaptor specific for this receptor. In addition, we have confirmed in a larger number of patients that the ISG IFIT1 downstream is also involved in the regulation of this ligand. Finally, we have confirmed that pre-isolated cDC2 can upregulate MICab expression upon exposure to Poly I:C, suggesting that the presence of other cells is not strictly required for the process (see new figure 4). Additional contribution of other cell types to IFN environment in pSS has also been further discussed.

2-We have directly addressed the potential implication of IL-15 in the crosstalk between cDC2 and NK cells following two different approaches: a) by analyzing the transcriptional levels of IL-15 in cDC cultured in media or in Poly I:C (see new Supplemental Figure 7D, b) by comparing the impact of anti-IL-15 neutralizing Ab with anti-MICa/b Ab on maturation of NK in co-cultures with Poly I:C primed cDC2 (see new Supplemental Figure 7E).

3- We have corroborated with a larger number of samples that bidirectional crosstalk between cDC2-NK cells increases activation of Th17 cells *in vitro*, in contrast to individual cDC2 or NK cells (see new figure 6C).

4-We now provide new data showing that interaction of cDC2 primed with Poly I:C with autologous NK cells leads to increased secretion of IL-6 and TNFα, which have been involved in Th17 priming. Therefore, the new data support that bidirectional communication between the two innate immune cell types modifies functional properties of cDC2 (see new Figure 6D).

All the previous reviews noted that the use of poly I:C treated mice as an in vivo model of Sjogren's Syndrome is not appropriate (R1-Q5, R2-Q8, R3-Significance and paragraph 9). The authors somewhat acknowledge the limitations of their chosen model but remain convinced that it is appropriate as is allows them to investigate glandular hypofunction and immune infiltration. NK cell responses and activation of DCs and monocytes can be examined in a poly I:C treatment model, and in fact some of the responses observed would be expected.

We would like to thank the reviewer for acknowledging that the Poly I:C injection *in vivo* model can be useful to study dynamics of innate immune activation and their impact on cDC and NK cells. We agree that based on our previous RNA-seq data and current knowledge, some of the findings such as increased activation or recruitment of cDC and NK cells in the SG in these mice can be expected. However, we would like to highlight that the model has been useful to validate some biomarkers that we have previously identified in pSS patients such as increased expression of NKG2D ligands, CD64 on cDC2 and increased transitional NK cells expressing higher levels of NKG2D. To our knowledge, our study is the first to describe these phenotypical changes in the mentioned innate immune populations in this *in vivo* model.

However, the model cannot be used to examine mechanisms relevant to Sjogren's Syndrome, a pathology that is autoimmune in nature. Nandula et al (PMID:

22672212, Ref 32) noted that hyposalivation in poly I:C treated mice is transient and likely the result of type I IFN and IL-6 interference with Ca(2+) mobilization directly within acinar cells - thus not autoimmune in nature. Therefore, this system is not suited to in vivo modelling of the autoreactive immune responses that drive glandular pathology in Sjogren's Syndrome.
Although we acknowledge that the *in vivo* model is not based in the induction of specific adaptive immune responses directed against salivary gland/self antigens and has been described to induce transient inflammation, which we have confirmed occurs during times of Poly I:C injections. However, in accordance to original publications in this model, we also have observed that once Poly I:C is not administered and acute inflammation has declined, we continue to observe glandular hypofunction at 3 weeks post injection. Moreover, we have shown that both at 3 and even at 8 weeks after poly I:C injection we can detect immune aggregates in the gland that include accumulations of B cells, and also higher frequencies of Th17 cells, which are typically present in SG from pSS patients. Moreover, B cells present in the infiltrates display a memory B cell phenotype, more susceptible to induce autoantibodies. Therefore, this *in vivo* system allows to recapitulate some key aspects of pSS pathology although it does not completely mimic the disorder present in the patient, as commonly happens in other alternative mice models (PMID 25777752). A potential explanation may consist in that although the poly I:C injection *in vivo* model is not based on the immunization of a salivary gland antigen, the inflammation induced by Poly I:C may lead to the release of damage associated molecular patterns and may allow to certain degree to temporarily disrupt peripheral tolerance in these mice. This possibility has been further discussed in our revised manuscript.

Several additional concerns remain to be addressed, including of note how activated NK cells affect cDC2 fate; the authors indicate they have data to address this, but do not provide it.

As detailed above, we have included in our revised manuscript several additional sets of new data providing relevant new information on the mechanisms involved in the interactions between cDC2 and NK cells and the functional impact on cDC2 or the priming of Th17 responses in vitro (new figure 6, new Supplemental figures 7 and 11).

In sum, this manuscript reports on interesting observations in samples from patients with Sjogren's Syndrome but does not provide evidence of how they relate to disease. The manuscript does not demonstrate that activated cytotoxic NK cells contribute to autoimmune pathology.

Our data demonstrates that the presence of NK cells is required for the induction of pathogenic Th17 cells in the SG in the Poly I:C *in vivo* model. In addition, we have shown that the presence of NK cells alone is not sufficient to induce pathogenic Th17 cells, but rather the synergistic interaction between NK and cDC2 may contribute to this process.

How interactions between NK cells and cDC2s (via NKG2D) result in enhanced formation of autoreactive T and B cells remains unclear.

We have shown that NKG2D-MICab interactions, but not IL-15 dependent mechanisms are accounting for increased activation of NK cells. Also, we have shown that bidirectional crosstalk between NK and cDC2, associates with higher activation of Th17 cells. Moreover, we have shown that cDC2 exposed to NK increased expression of IL-6, TNFα, which may facilitate the priming of Th17 and differentiation of B cells.

The concerns outlined above lead me to conclude that as submitted, the manuscript is not suited for publication in The EMBO Journal.
We hope that the reviewer recognizes that our revised manuscript addressing most of both the previous and most recent concerns and that it represents a significantly improved version of our study, and therefore, is now suited for publication in EMBO Journal.

Dear Enrique,

Thank you for submitting you revised manuscript to The EMBO Journal. Your study has now been seen by the original referee whose comments are provided below. As you can see, the referee appreciates that the analysis has been strengthened. I agree with the suggestion from the referee to remove some of the less developed datasets and focus the study on the key parts and would like to ask you to respond to the remaining concerns.

When you resubmit the revised version please also take care of the following editorial points:

- Please add full author names in manuscript (first name, last name)

- Funding info is missing in the online submission system for Fondo Europeo de Desarrollo Regional (FEDER), The European Regional Development Fund (ERDF) "A way to make Europe"

- We need 3-5 keywords

- Reference format needs to be alphabetical, et al should be used after 10 author names

- Please make sure that GSE194263 is publicly available

- COI should be renamed to "Disclosure statement and competing interests"

- Please remove the Authors Contributions from the manuscript. The 'Author Contributions' section is replaced by the CRediT contributor roles taxonomy to specify the contributions of each author in the journal submission system. Please use the free text box in the 'author information' section of the manuscript submisssion system to provide more detailed descriptions (e.g., 'X provided intracellular Ca++ measurements in fig Y')

- The individual figure panels for figure 3 need to be called out sequentially - e.g. we can't have Figure 3F called out before Figure 3D-E

- The supplemental file should be labelled as Appendix with a ToC and files renamed to Appendix Figure S1, etc. with callouts in the MS updated accordingly. Please also add the figure legends to this file. The Supplemental tables should also be inserted in the Appendix.

- We still need source data. Please see email from Hannah Sonntag (h.sonntag@source-data.org) on the 29th of Marcah

- Please upload a synopsis text. We need a summary statement plus 3-5 bullet points describing the key findings of the MS.

- We also need a synopsis image => 550 wide by [200-400]

- Our publisher has also done their pre-publication check on your manuscript. When you log into the manuscript submission system you will see the file "Data Edited Manuscript file". Please look at the word file and the comments regarding the figure legends and respond to the issues.

- Is the FACS blot used in supplemental figure 9C (NK1.1) the same as the one shown in supplemental figure 10B? If so, then please mention this in the figure legend.

- Data Availability section should go before Acknowl. and after Materials and Methods

That should be all. Let me know if you have any further questions.

Best Karin

Karin Dumstrei, PhD
Senior Editor
The EMBO Journal

Instructions for preparing your revised manuscript:

Guide For Authors: https://www.embopress.org/page/journal/14602075/authorguide

Use the link below to submit your revision:

https://emboj.msubmit.net/cgi-bin/main.plex

Referee #1:

The revised manuscript provides new and valuable information about the molecular pathways of innate RNA sensing and IFNI activation that lead to changes in NK cells and cDC2 in patients with primary Sjogren's Syndrome.

The mouse model is helpful in part as it supports the concept that the changes observed in NK cells and cDC observed in patients can be phenocopied by activating an innate RNA sensing pathway via delivery of poly-IC.

I remain unconvinced by the use of the mouse model and the data provided to support the notion that NK and cDC2 crosstalk results in activation of pathogenic adaptive immune responses, in particular induction of pathogenic Th17 T cells as claimed by the authors. In my opinion these data weaken the central message of the paper which is that RNA sensing induces cDC2 activation and affects NK cell subsets, these being key events in the generation of autoimmunity. I suggest that mouse data should be confined solely to analyses of NK cells and DCs.

Specific points

1. Remove the term transitional NK cells and refer to the population as CD16+ CD56hi. Also ensure that in text and figures the use of hi or + is consistent (see figure 1B).
2. Please show actual data for the CD56-CD16+ subset as Table 2 does not accurately represent what appears to be a major difference as shown in figure 1A.
3. Figure 1E-F: the data show more granzyme B+ NK cells not higher granzyme B levels as stated on pages 9-10.
4. Figure 1G does not show NK cells "in close proximity to IL17+ and B cells", none of the CD19 signal is close to the CD56 signal and most of the IL17+ cells are not close to CD56+ ones.
5. Page 10 "proportions of inflammatory NC Mo were significantly increased in PB of pSS patients" - no data are shown to support this statement.
6. Figure 2, fix to show CD14+ cells first as per text.
7. Page 11 "although they were not restored in the OT group" - change to not affected.
8. Page 11 "cDC1 were also depleted" - change to cDC1 were also lower.
9. Figure 2D, HLA-DR stain: the signal is poor and does not appear to colocalize with MICab. The background seems too high for CD1c. Please check.
10. Mouse salivary glands have resident populations of ILC1/NK cells that are also NK1.1+CD3-, please note and discuss.
11. For mouse data both percentages and numbers of NK cells and DCs must be presented.
12. Remove the mouse data relating to B cells, T cells etc. There is insufficient evidence and too much speculation. Intermixing human data (co-cultures) to support mechanisms in the mouse is not helpful. Mouse studies can be the subject of future work.
13. In the context of point 11 above, much of the study focuses on showing that NK cells have the potential to be cytotoxic in pSS, but on page 22 the authors speculate that "NK cells may modify functional properties of poly-IC activated cDC2". These are inconsistent statements.
14. Reference 10 is used incorrectly as this is a mouse study.
15. Please check spelling in Figures; see figure 3B "cannonical" is incorrect.

POINT BY POINT RESPONSE TO REVIEWER

The revised manuscript provides new and valuable information about the molecular pathways of innate RNA sensing and IFNI activation that lead to changes in NK cells and cDC2 in patients with primary Sjogren's Syndrome.
We would like to thank the reviewer for highlighting the improvement of the revised manuscript

The mouse model is helpful in part as it supports the concept that the changes observed in NK cells and cDC observed in patients can be phenocopied by activating an innate RNA sensing pathway via delivery of poly-IC.
We would like to thank the reviewer for acknowledging the value of the *in vivo* data

I remain unconvinced by the use of the mouse model and the data provided to support the notion that NK and cDC2 crosstalk results in activation of pathogenic adaptive immune responses, in particular induction of pathogenic Th17 T cells as claimed by the authors. In my opinion these data weaken the central message of the paper which is that RNA sensing induces cDC2 activation and affects NK cell subsets, these being key events in the generation of autoimmunity. I suggest that mouse data should be confined solely to analyses of NK cells and DCs.
We agree the Th17 data may distract from the main message of the manuscript and we have followed the reviewer´s recommendation and focused on the cDC2 and NK cell data from the in vivo model.

Specific points

1. Remove the term transitional NK cells and refer to the population as CD16+ CD56hi. Also ensure that in text and figures the use of hi or + is consistent (see figure 1B).
The "transitional" term has been removed and substituted for "CD16+ CD56hi" to refer to this NK subset in the manuscript, legends and figures as requested by the reviewer.

2. Please show actual data for the CD56-CD16+ subset as Table 2 does not accurately represent what appears to be a major difference as shown in figure 1A.
To address this point we have added two new more representative dot plots from the two study pSS and control cohorts in which proportions of each population (including the CD56-CD16+ NK cell subset) are representative from the data shown in Appendix Table S2.

3. Figure 1E-F: the data show more granzyme B+ NK cells not higher granzyme B levels as stated on pages 9-10.

To satisfy the reviewer´s request and avoid confusion, statement has been changed in the main text from the manuscript to ensure that the data shown refers to detection of cells co-expressing Granzyme B and CD56 in the figure 1 E-F.

4. Figure 1G does not show NK cells "in close proximity to IL17+ and B cells", none of the CD19 signal is close to the CD56 signal and most of the IL17+ cells are not close to CD56+ ones.
To address the reviewer comment, we have changed the sentence to “NK cells were detected in highly infiltrated areas from SG containing IL-17+ and B cells ”

5. Page 10 "proportions of inflammatory NC Mo were significantly increased in PB of pSS patients" - no data are shown to support this statement.
Proportions of inflammatory NC Mo from pSS patients was indeed shown in the right area from panel A of the previous Figure 2. We have now highlighted that these data are now shown in the left area of Figure 2A before the DC is described, to help the reader follow the order in which results are described in the text.

6. Figure 2, fix to show CD14+ cells first as per text.
As mentioned, we have followed the reviewer´s request and changed the panel in which CD16+ CD14lo Mo data is shown to match the mention order in the text.

7. Page 11 "although they were not restored in the OT group" - change to not affected.
Thanks for the suggestion, the sentence has been changed in the main text accordingly.

8. Page 11 "cDC1 were also depleted" - change to cDC1 were also lower.
Sentence has been changed in the main text as requested.

9. Figure 2D, HLA-DR stain: the signal is poor and does not appear to colocalize with MICab. The background seems too high for CD1c. Please check.
To address this concern, we have now included a new image with more evident co-staining of MICab on cells expressing high levels of CD1c and HLA-DR. We have also included the quantification of proportions of CD1c+ HLA-DR+ cells co-expressing MICab in these tissue sections. Finally, we have also included a representative image of a control salivary gland in the supplemental material from our manuscript (New Appendix figure S4).

10. Mouse salivary glands have resident populations of ILC1/NK cells that are also NK1.1+CD3-, please note and discuss.
The presence of ILC1/NK cells in the salivary gland and their potential implication in pSS has been discussed in the revised manuscript.

11. For mouse data both percentages and numbers of NK cells and DCs must be presented.

Absolute numbers of cDC and NK has now been included in the supplemental material and mentioned in the text of the revised manuscript.

12. Remove the mouse data relating to B cells, T cells etc. There is insufficient evidence and too much speculation. Intermixing human data (co-cultures) to support mechanisms in the mouse is not helpful. Mouse studies can be the subject of future work.
We acknowledge that the Th17 data is not essential for the main message of the manuscript and we will use it for subsequent studies.

13. In the context of point 11 above, much of the study focuses on showing that NK cells have the potential to be cytotoxic in pSS, but on page 22 the authors speculate that "NK cells may modify functional properties of poly-IC activated cDC2". These are inconsistent statements.
The statement has been removed since it was intended to provide a potential explanation for the increased Th17 response by cDC after interaction with NK cells.

14. Reference 10 is used incorrectly as this is a mouse study.
We apologize for the mistake. The reference has been removed and substituted by a new citation.

15. Please check spelling in Figures; see figure 3B "cannonical" is incorrect.
We apologize for the mistake. The spelling error has been corrected

RESPONSE TO EDITORIAL QUESTION

The individual figure panels for figure 3 need to be called out sequentially - e.g. we can't have Figure 3F called out before Figure 3D-E

We have reviewed the mention of the different figure panels throughout the manuscript and ensured that it is sequential. We have confirmed that Figure 3F is not mentioned before figure 3D-E.

Dear Enrique,

Thank you for submitting your revised manuscript to The EMBO Journal. I have now had a chance to take a look at it and I appreciate the introduced changes.

I am therefore very pleased to accept the manuscript for publication here.

Congratulations on a nice study!

best Karin

Karin Dumstrei, PhD
Senior Editor
The EMBO Journal

Please note that it is EMBO Journal policy for the transcript of the editorial process (containing referee reports and your response letter) to be published as an online supplement to each paper. If you do NOT want this, you will need to inform the Editorial Office via email immediately. More information is available here:
https://www.embopress.org/page/journal/14602075/authorguide#transparentprocess

Your manuscript will be processed for publication in the journal by EMBO Press. Manuscripts in the PDF and electronic editions of The EMBO Journal will be copy edited, and you will be provided with page proofs prior to publication. Please note that supplementary information is not included in the proofs.

You will be contacted by Wiley Author Services to complete licensing and payment information. The required 'Page Charges Authorization Form' is available here: https://www.embopress.org/pb-assets/embo-site/tej_apc.pdf - please download and complete the form and return to embopressproduction@wiley.com

EMBO Press participates in many Publish and Read agreements that allow authors to publish Open Access with reduced/no publication charges. Check your eligibility: https://authorservices.wiley.com/author-resources/Journal-Authors/open-access/affiliation-policies-payments/index.html

Should you be planning a Press Release on your article, please get in contact with embojournal@wiley.com as early as possible, in order to coordinate publication and release dates.

If you have any questions, please do not hesitate to call or email the Editorial Office. Thank you for your contribution to The EMBO Journal.

** Click here to be directed to your login page: https://emboj.msubmit.net

EMBO Press Author Checklist

Corresponding Author Name: ENRIQUE MARTIN GAYO	
Journal Submitted to: THE EMBO JOURNAL	
Manuscript Number: EMBOJ-2023-113714R	

USEFUL LINKS FOR COMPLETING THIS FORM
The EMBO Journal - Author Guidelines
EMBO Reports - Author Guidelines
Molecular Systems Biology - Author Guidelines
EMBO Molecular Medicine - Author Guidelines

Reporting Checklist for Life Science Articles (updated January
This checklist is adapted from Materials Design Analysis Reporting (MDAR) Checklist for Authors. MDAR establishes a minimum set of requirements in transparent reporting in the life sciences (see Statement of Task: 10.31222/osf.io/9sm4x). Please follow the journal's guidelines in preparing your
Please note that a copy of this checklist will be published alongside your article.

Abridged guidelines for figures
1. Data
The data shown in figures should satisfy the following conditions:
- the data were obtained and processed according to the field's best practice and are presented to reflect the results of the experiments in an accurate and unbiased manner.
- ideally, figure panels should include only measurements that are directly comparable to each other and obtained with the same assay.
- plots include clearly labeled error bars for independent experiments and sample sizes. Unless justified, error bars should not be shown for technical
- if n<5, the individual data points from each experiment should be plotted. Any statistical test employed should be justified.
- Source Data should be included to report the data underlying figures according to the guidelines set out in the authorship guidelines on Data

2. Captions
Each figure caption should contain the following information, for each panel where they are relevant:
- a specification of the experimental system investigated (eg cell line, species name).
- the assay(s) and method(s) used to carry out the reported observations and measurements.
- an explicit mention of the biological and chemical entity(ies) that are being measured.
- an explicit mention of the biological and chemical entity(ies) that are altered/varied/perturbed in a controlled manner.
- the exact sample size (n) for each experimental group/condition, given as a number, not a range;
- a description of the sample collection allowing the reader to understand whether the samples represent technical or biological replicates (including how many animals, litters, cultures, etc.).
- a statement of how many times the experiment shown was independently replicated in the laboratory.
- definitions of statistical methods and measures:
  - common tests, such as t-test (please specify whether paired vs. unpaired), simple $\chi 2$ tests, Wilcoxon and Mann-Whitney tests, can be unambiguously identified by name only, but more complex techniques should be described in the methods section;
  - are tests one-sided or two-sided?
  - are there adjustments for multiple comparisons?
  - exact statistical test results, e.g., P values = x but not P values < x;
  - definition of 'center values' as median or average;
  - definition of error bars as s.d. or s.e.m.

Please complete ALL of the questions below.
Select "Not Applicable" only when the requested information is not relevant for your study.

Materials

Newly Created Materials	Information included in the manuscript?	In which section is the information available? (Reagents and Tools Table, Materials and Methods, Figures, Data Availability Section)
New materials and reagents need to be available; do any restrictions apply?	Not Applicable	

Antibodies	Information included in the manuscript?	In which section is the information available? (Reagents and Tools Table, Materials and Methods, Figures, Data Availability Section)
For **antibodies** provide the following information:<br>- Commercial antibodies: RRID (if possible) or supplier name, catalogue number and or/clone number<br>- Non-commercial: RRID or citation	Yes	Supplemental Table 5

DNA and RNA sequences	Information included in the manuscript?	In which section is the information available? (Reagents and Tools Table, Materials and Methods, Figures, Data Availability Section)
**Short novel DNA or RNA including primers, probes**: provide the sequences.	Yes	Supplemental Table 5

Cell materials	Information included in the manuscript?	In which section is the information available? (Reagents and Tools Table, Materials and Methods, Figures, Data Availability Section)
**Cell lines:** Provide species information, strain. Provide accession number in repository **OR** supplier name, catalog number, clone number, and/**OR** RRID.	Yes	Supplemental Table 5
**Primary cultures:** Provide species, strain, sex of origin, genetic modification status.	Yes	Supplemental Table 5
Report if the cell lines were recently **authenticated** (e.g., by STR profiling) and tested for mycoplasma contamination.	Not Applicable	

Experimental animals	Information included in the manuscript?	In which section is the information available? (Reagents and Tools Table, Materials and Methods, Figures, Data Availability Section)
**Laboratory animals or Model organisms:** Provide species, strain, sex, age, genetic modification status. Provide accession number in repository **OR** supplier name, catalog number, clone number, **OR** RRID.	Yes	Supplemental Table 5. Methods in in vivo model section. Figures 5 and Supplemental Figures 9 and 10
**Animal observed in or captured from the field:** Provide species, sex, and age where possible.	Not Applicable	
Please detail **housing and husbandry conditions**.	Yes	Supplemental Table 5. Methods in in vivo model section and functional DCs assays.

Plants and microbes	Information included in the manuscript?	In which section is the information available? (Reagents and Tools Table, Materials and Methods, Figures, Data Availability Section)
**Plants:** provide species and strain, ecotype and cultivar where relevant, unique accession number if available, and source (including location for collected wild specimens).	Not Applicable	
**Microbes:** provide species and strain, unique accession number if available, and source.	Not Applicable	

Human research participants	Information included in the manuscript?	In which section is the information available? (Reagents and Tools Table, Materials and Methods, Figures, Data Availability Section)
If collected and within the bounds of privacy constraints report on age, sex and gender or ethnicity for all study participants.	Yes	Supplemental Table 1. Methods in the "Study partipants" section

Core facilities	Information included in the manuscript?	In which section is the information available? (Reagents and Tools Table, Materials and Methods, Figures, Data Availability Section)
If your work benefited from core facilities, was their service mentioned in the acknowledgments section?	Yes	ACKNOWLEDGMENTS section

Design

Study protocol	Information included in the manuscript?	In which section is the information available? (Reagents and Tools Table, Materials and Methods, Figures, Data Availability Section)
If study protocol has been **pre-registered, provide DOI in the manuscript**. For clinical trials, provide the trial registration number **OR** cite DOI.	Yes	doi.org/10.1101/2022.03.13.484063
Report the **clinical trial registration number** (at ClinicalTrials.gov or equivalent), where applicable.	Not Applicable	

Laboratory protocol	Information included in the manuscript?	In which section is the information available? (Reagents and Tools Table, Materials and Methods, Figures, Data Availability Section)
Provide DOI OR other citation details if **external detailed step-by-step protocols** are available.	Not Applicable	

Experimental study design and statistics	Information included in the manuscript?	In which section is the information available? (Reagents and Tools Table, Materials and Methods, Figures, Data Availability Section)
Include a statement about **sample size** estimate even if no statistical methods were used.	Yes	Material and Methods in "Study participants" and "statistics" sections
Were any steps taken to minimize the effects of subjective bias when allocating animals/samples to treatment (e.g. **randomization procedure**)? If yes, have they been described?	Yes	Supplemental Table 5. Methods in in vivo model section and functional DCs assays.
Include a statement about **blinding** even if no blinding was done.	Yes	Blinding statement has been included in the Methods section
Describe **inclusion/exclusion criteria** if samples or animals were excluded from the analysis. Were the criteria pre-established?<br><br>If sample or data points were omitted from analysis, report if this was due to attrition or intentional exclusion and provide justification.	Yes	An statement has been inclued in the corresponding Methods section
For every figure, are **statistical tests** justified as appropriate? Do the data meet the assumptions of the tests (e.g., normal distribution)? Describe any methods used to assess it. Is there an estimate of variation within each group of data? Is the variance similar between the groups that are being statistically compared?	Yes	Statistical test used for every figure has been detailed in legends throughout the manuscript. An statement of the rationale of each analysis is also included in the statistics methods section.

Sample definition and in-laboratory replication	Information included in the manuscript?	In which section is the information available? (Reagents and Tools Table, Materials and Methods, Figures, Data Availability Section)
In the figure legends: state number of times the experiment was **replicated** in laboratory.	Yes	Number of replicates from each experiment are detailed in all figure legends
In the figure legends: define whether data describe **technical or biological replicates**.	Yes	The figure legends include information regarding the technical or biological replicate nature for each experiment.

Ethics

Ethics	Information included in the manuscript?	In which section is the information available? (Reagents and Tools Table, Materials and Methods, Figures, Data Availability Section)
Studies involving **human participants**: State details of **authority granting ethics approval** (IRB or equivalent committee(s), provide reference number for approval.	Yes	*Material and Methods in "Study participants" and "Ethic statement" sections*
Studies involving **human participants**: Include a statement confirming that **informed consent** was obtained from all subjects and that the experiments conformed to the principles set out in the WMA Declaration of Helsinki and the Department of Health and Human Services Belmont Report.	Yes	Material and Methods in "Study participants" and "Ethic statement" sections
Studies involving **human participants:** For publication of **patient photos**, include a statement confirming that consent to publish was obtained.	Not Applicable	
Studies involving experimental **animals**: State details of **authority granting ethics approval** (IRB or equivalent committee(s), provide reference number for approval. Include a statement of compliance with ethical regulations.	Yes	Material and Methods in "Ethic statement" section
Studies involving **specimen and field samples:** State if relevant **permits** obtained, provide details of authority approving study; if none were required, explain why.	Not Applicable	

Dual Use Research of Concern (DURC)	Information included in the manuscript?	In which section is the information available? (Reagents and Tools Table, Materials and Methods, Figures, Data Availability Section)
Could your study fall under dual use research restrictions? Please check biosecurity documents and list of **select agents and toxins** (CDC): https://www.selectagents.gov/sat/list.htm	Not Applicable	
If you used a select agent, is the security level of the lab appropriate and reported in the manuscript?	Not Applicable	
If a study is subject to dual use research of concern regulations, is the name of the **authority granting approval and reference number** for the regulatory approval provided in the manuscript?	Not Applicable	

Reporting

The MDAR framework recommends adoption of discipline-specific guidelines, established and endorsed through community initiatives. Journals have their own policy about requiring specific guidelines and recommendations to complement MDAR.

Adherence to community standards	Information included in the manuscript?	In which section is the information available? (Reagents and Tools Table, Materials and Methods, Figures, Data Availability Section)
State if relevant guidelines or checklists (e.g., **ICMJE, MIBBI, ARRIVE, PRISMA**) have been followed or provided.	Not Applicable	
For **tumor marker prognostic studies,** we recommend that you follow the **REMARK** reporting guidelines (see link list at top right). See author guidelines, under 'Reporting Guidelines'. Please confirm you have followed these guidelines.	Not Applicable	
For **phase II and III randomized controlled trials**, please refer to the **CONSORT** flow diagram (see link list at top right) and submit the CONSORT checklist (see link list at top right) with your submission. See author guidelines, under 'Reporting Guidelines'. Please confirm you have submitted this list.	Not Applicable	

Data Availability

Data availability	Information included in the manuscript?	In which section is the information available? (Reagents and Tools Table, Materials and Methods, Figures, Data Availability Section)
Have **primary datasets** been deposited according to the journal's guidelines (see 'Data Deposition' section) and the respective accession numbers provided in the Data Availability Section?	Yes	Material and Methods in "Data availability" section
Were **human clinical and genomic datasets** deposited in a public access-controlled repository in accordance to ethical obligations to the patients and to the applicable consent agreement?	Yes	RNAseq data has been deposited in a public repository as detailed in the Methods section
Are **computational models** that are central and integral to a study available without restrictions in a machine-readable form? Were the relevant accession numbers or links provided?	Yes	Accesion number of transcriptional data is provided
If publicly available data were reused, provide the respective **data citations in the reference list**.	Not Applicable	
